# Development of Synthetic Routes to 2′‐*O*,4′‐*C*‐Spirocyclopentylene‐Bridged Nucleic Acids: Thymidine, Guanosine, and Adenosine

**DOI:** 10.1002/chem.202502995

**Published:** 2025-11-07

**Authors:** Riku Kumagai, Riko Yamada, Takao Yamaguchi, Satoshi Obika

**Affiliations:** ^1^ School of Pharmaceutical Sciences The University of Osaka 1–6 Yamadaoka Suita Osaka 565‐0871 Japan; ^2^ Graduate School of Pharmaceutical Sciences The University of Osaka 1–6 Yamadaoka Suita Osaka 565‐0871 Japan

**Keywords:** iodocyclization, modified nucleic acid, nucleoside synthesis, oligonucleotide therapeutics, transglycosylation

## Abstract

Modified nucleic acids are crucial for improving the efficacy and safety of antisense oligonucleotides (ASOs). We previously synthesized and evaluated 2′‐*O*,4′‐*C*‐spirocyclopentylene‐bridged nucleic acid (scpBNA2) bearing the pyrimidine nucleobases thymine (T) and 5‐methylcytosine (^m^C). Oligonucleotides incorporating scpBNA2 exhibited strong binding affinity toward complementary single‐stranded RNA (ssRNA) and high resistance to nuclease degradation. These favorable properties enabled ASOs containing scpBNA2‐T and scpBNA2‐^m^C in the wing regions to induce potent target RNA knockdown both *in vitro* and *in vivo*. Furthermore, replacing 2′‐*O*,4′‐*C*‐methylene‐bridged nucleic acid/locked nucleic acid (2′,4′‐BNA/LNA) with scpBNA2 significantly reduced hepatotoxicity, underscoring its potential as a promising nucleic acid analog for ASO design. In this study, we established efficient synthetic routes to scpBNA2 nucleosides bearing the purine nucleobases guanine (G) and adenine (A), in addition to the previously reported scpBNA2‐T. The scpBNA2‐T nucleoside was synthesized *via* 2′,4′‐lactonization followed by formation of a 2,2′‐anhydropyrimidine intermediate. For scpBNA2‐G and scpBNA2‐A, we devised a robust strategy that combines late‐stage transglycosylation—converting the thymine nucleobase into purines—with an iodocyclization reaction to construct the 2′,4′‐bridged framework. This concise approach provides access to scpBNA2 nucleosides and supports their broader applications in ASO development.

## Graphical abstract

A novel synthetic route to 2′‐*O*,4′‐*C*‐spirocyclopentylene‐bridged nucleic acid (scpBNA2) monomers bearing thymine, guanine, and adenine nucleobases. The strategy integrates 2′,4′‐lactonization, transglycosylation, and intramolecular iodocyclization, thereby bypassing the 2′‐OH inversion step and providing scpBNA2 nucleoside building blocks for antisense oligonucleotides (ASOs).

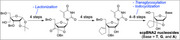

## 1 Introduction

**Figure 1:**
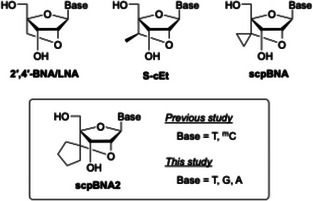
Chemical structures of 2′,4′‐BNA/LNA, *S*‐cEt, scpBNA, and scpBNA2 nucleosides. The term “Base” denotes the nucleobase.

Antisense oligonucleotides (ASOs) hybridize with complementary mRNA to form a heteroduplex that triggers RNase H1‐dependent cleavage, thereby suppressing protein translation.^[^ ^1^, ^2^ ^]^ For efficient knockdown activity, ASOs must exhibit an appropriate duplex‐forming ability toward the target mRNA as well as strong resistance to nuclease degradation. To improve these properties, numerous chemical modifications have been introduced into ASOs.^[^ ^3^, ^4^ ^]^ Among the modified nucleic acids reported, 2′‐*O*,4′‐*C*‐methylene‐bridged nucleic acid, also known as locked nucleic acid (2′,4′‐BNA/LNA; Figure 1), has demonstrated excellent duplex‐forming ability toward single‐stranded RNA (ssRNA) and moderate nuclease resistance. The enhanced duplex‐forming ability is largely attributed to the enforced N‐type (C3′‐endo) sugar pucker, which preorganizes the backbone into an RNA‐like A‐form geometry.^[^ ^5^, ^6^, ^7^, ^8^ ^]^ Since its discovery, many derivatives based on the 2′,4′‐BNA/LNA framework have been synthesized and evaluated.^[^ ^3^, ^9^ ^]^ Structural variations at the bridge position—including substitution or expansion of the bridge ring size—have been shown to further enhance nuclease stability by sterically hindering enzymatic cleavage.^[^ ^9^, ^10^, ^11^, ^12^ ^]^ Building on these insights, researchers at Ionis Pharmaceuticals developed the (*S*)‐constrained ethyl (*S*‐cEt) analog (Figure 1),^[^ ^13^, ^14^, ^15^ ^]^ which introduces a methyl substituent into the bridge and has been successfully applied in clinical drug candidates.^[^ ^16^, ^17^, ^18^, ^19^ ^]^

We previously designed and synthesized 2′‐*O*,4′‐*C*‐spirocyclopropylene‐bridged nucleic acid (scpBNA; Figure 1),^[^ ^20^, ^21^ ^]^ which incorporates a cyclopropane ring to impart conformational rigidity and steric bulkiness. Unlike *S*‐cEt, scpBNA can be synthesized without diastereomer separation, while retaining strong RNA affinity and high nuclease resistance. To further expand this class, we recently developed 2′‐*O*,4′‐*C*‐spirocyclopentylene‐bridged nucleic acid (scpBNA2; Figure 1), in which the 2′,4′‐bridge contains a cyclopentane ring.^[^ ^22^ ^]^ The enlarged ring further improved nuclease stability, likely by increasing steric shielding of the phosphodiester backbone. ASOs incorporating scpBNA or scpBNA2 exhibited potent antisense activity both *in vitro* and *in vivo*, while showing markedly reduced hepatotoxicity compared with ASOs containing 2′,4′‐BNA/LNA.^[^ ^22^ ^]^ This reduction in hepatotoxicity is thought to result from diminished interactions between the modified ASOs and hepatotoxicity‐related proteins, conferred by the cyclopropane or cyclopentane moieties.

To date, however, investigations of scpBNA2 have been limited to pyrimidine analogs, specifically thymine (T) and 5‐methylcytosine (^m^C).^[^ ^22^ ^]^ To fully explore the therapeutic potential of the scpBNA2, synthesis of the corresponding purine nucleosides is essential.

In this study, we first synthesized a novel 2′,4′‐lactone‐bridged thymidine intermediate, which enabled efficient preparation of the scpBNA2‐T nucleoside. We then developed a new synthetic approach to construct the 2′,4′‐bridge that circumvents the low‐yielding inversion of the 2′‐hydroxy group—a major limitation previously encountered in scpBNA‐purine derivatives.^[^ ^21^ ^]^ Using this strategy, we successfully synthesized both scpBNA2‐guanine (scpBNA2‐G) and scpBNA2‐adenine (scpBNA2‐A) nucleosides, thereby completing the full set of scpBNA2 monomers for ASO design.

## 2 Results and Discussion

### 2.1 Synthesis of scpBNA2‐T Nucleoside *via* a 2′,4′‐Ester‐Bridged Thymidine Analog

**Figure 2:**
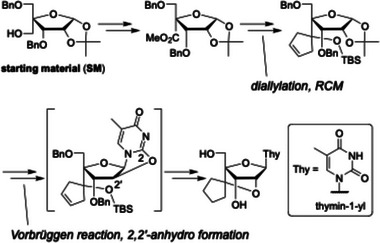
Conventional synthetic route to the scpBNA2‐T nucleoside.

Previously, the scpBNA2‐T nucleoside was synthesized from 3,5‐di‐*O*‐benzyl‐4‐*C*‐hydroxymethyl‐1,2‐*O*‐isopropylidene‐α‐D‐ribofuranose (starting material; SM) through a route involving methyl ester formation, diallylation, ring‐closing metathesis (RCM), and a Vorbrüggen reaction to introduce the thymine nucleobase (Figure 2).^[^ ^20^, ^22^ ^]^ In this strategy, the 2‐carbonyl group of thymine base was subsequently exploited to construct the 2′,4′‐bridge by forming a 2,2′‐anhydro intermediate.

To establish a more concise and streamlined synthesis of scpBNA2 nucleosides, we hypothesized that direct formation of a 2′,4′‐ester bridge *via* the 2′‐oxygen atom—followed by *gem*‐bisallylation without disrupting the bridged structure—would enable efficient access to the desired scaffold.

**Scheme 1:**
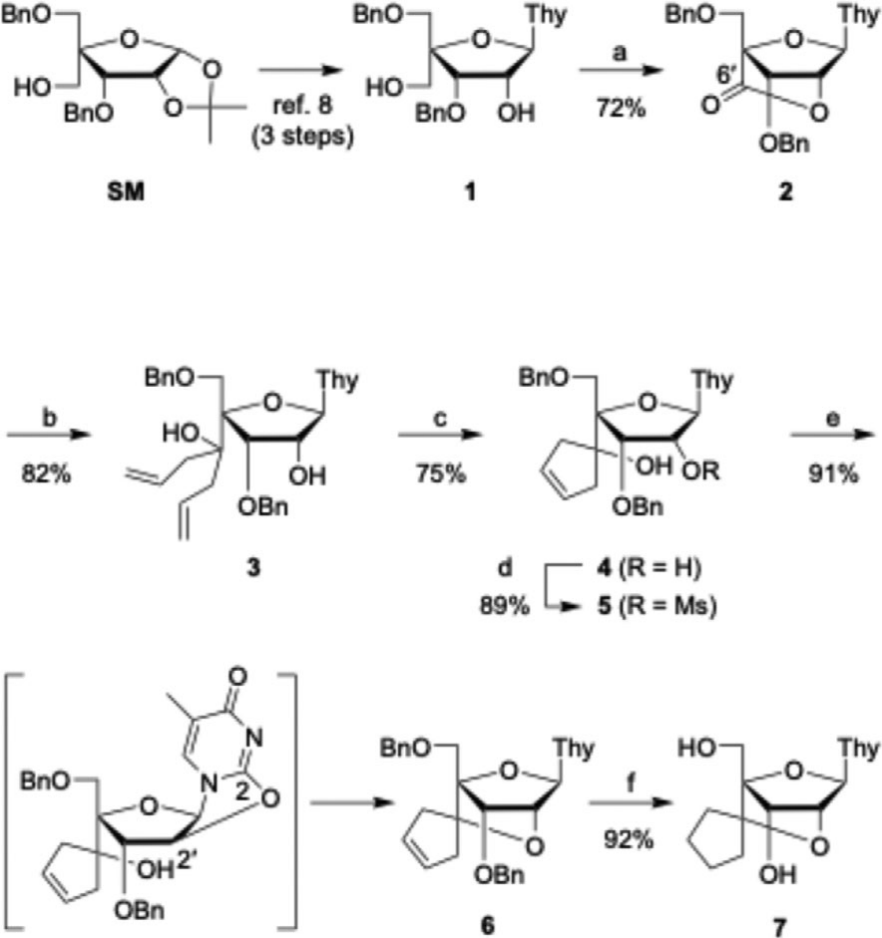
Novel synthetic route to scpBNA2‐T nucleoside **7**. *Reagents and conditions*: a) TEMPO, PhI(OAc)_2_, CH_2_Cl_2_, rt, 18 hours, 72%; b) allylMgBr, THF, –35 °C, 1 hour, 82%; c) Grubbs 2^nd^ generation cat., CH_2_Cl_2_, reflux, 8 hours, 75%; d) MsCl, pyridine, CH_2_Cl_2_, rt, 2 hours, 89%; e) K_2_CO_3_, DMF, 90 °C, 6 hours, 91%; f) H_2_, Pd(OH)_2_/C, AcOEt, rt, 50 minutes, 92%.

The synthesis began with compound **1** ^[^ ^8^ ^]^ (Scheme 1). Compound **1** was prepared in three steps (88% overall yield) from SM by slightly modifying the previously reported procedure (Scheme S1). TEMPO oxidation of **1** furnished compound **2**, which contains a 2′,4′‐ester bridge, in a single step. To the best of our knowledge, this is the first reported example of a nucleoside bearing a 2′,4′‐ester‐bridged structure. The IR spectrum of **2** displayed a strong carbonyl absorption at 1810 cm^−1^, consistent with the lactone formation. In addition, the ^1^H NMR spectrum showed characteristic singlet signals for the 1′, 2′, and 3′ protons, further confirming the bridged structure.

We next investigated *gem*‐bisallylation at the 6′ position, which was originally envisioned as a key transformation toward scpBNA2‐type nucleosides. Although a variety of conditions were tested (Table S1), direct *gem*‐bisallylation^[^ ^23^ ^]^ of compound **2** proved unsuccessful, and instead diallyl alcohol **3** was obtained in 82% yield. On the basis of this outcome, we chose to reconstruct the 2′,4′‐bridge at a later stage of the synthesis. Thus, compound **3** was subjected to RCM with 10 mol% of Grubbs’ 2^nd^‐generation catalyst to afford cyclopentenol **4**. Mesylation of the secondary hydroxy group provided **5**, which was treated with potassium carbonate in DMF to deliver the bridged compound **6** through formation of a 2,2′‐anhydropyrimidine intermediate. Finally, hydrogenation of the olefin and hydrogenolysis of the benzyl groups furnished the target scpBNA2‐T nucleoside **7**.

In total, scpBNA2‐T nucleoside **7** was synthesized in nine steps with an overall yield of 29%. By comparison, the previously reported synthesis from the same starting material required 13 steps and gave a 36% yield.^[^ ^20^, ^22^ ^]^ By eliminating TBS protection/deprotection steps and providing direct access to the key lactone intermediate **2**, we were able to shorten the sequence, albeit with a modest decrease in overall yield. As previously described,^[^ ^22^ ^]^ compound **7** can also serve as a precursor for the corresponding scpBNA2 phosphoramidite bearing a 5‐methylcytosine (^m^C) nucleobase.

### 2.2 Novel Synthetic Approach for scpBNA2‐T Nucleoside

**Figure 3:**
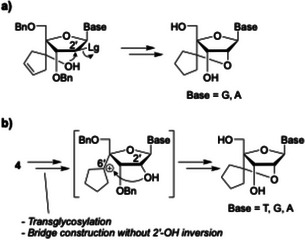
Synthetic strategies for scpBNA2‐T, ‐G, and ‐A nucleosides. a) Conventional bridge construction approach for the synthesis of scpBNA2‐G and ‐A. b) Novel strategy enabling the synthesis of scpBNA2‐T, ‐G, and ‐A *via* bridge construction without requiring stereoinversion at the 2′‐position.

In our current and previous studies,^[^ ^22^ ^]^ scpBNA2‐T was synthesized *via* a 2,2′‐anhydropyrimidine intermediate; however, this route is not applicable to scpBNA2‐purine derivatives.  As an alternative, we attempted the synthesis of scpBNA2‐G through an arabino‐type intermediate (Figure 3a), following the reported procedure for scpBNA‐G.^[^ ^21^ ^]^ The overall yield, however, was low, primarily due to the inefficiency of the 2′‐OH inversion step involving an oxidation–reduction sequence (Scheme S2).

To overcome this limitation, we devised a new strategy that circumvents the 2′‐OH inversion. Specifically, we envisioned constructing the 2′,4′‐bridge through intramolecular nucleophilic attack of the 2′‐hydroxy group on a tertiary carbocation generated at the 6′‐position (Figure 3b). As a model system, we again selected a thymine derivative because of its synthetic accessibility and the absence of a requirement for nucleobase protection.

**Scheme 2:**
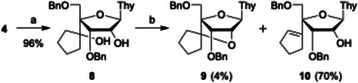
Acid‐promoted synthesis of the target S_N_1 product **9** and competing E1 elimination product **10**. *Reagents and conditions*: a) H_2_, Wilkinson's cat., THF, rt, 20 hours, 96%; b) TsOH·H_2_O, MeCN, 50 °C, 13 hours, 4% for **9** and 70% for **10**.

We first explored 2′,4′‐bridge formation *via* an acid‐promoted S_N_1 reaction (Scheme 2). Hydrogenation of the olefin moiety in cyclopentenol **4** with 10 mol% Wilkinson's catalyst afforded compound **8**. Treatment of **8** with *p*‐TsOH in acetonitrile at 50 °C yielded the desired S_N_1 product **9** in only 4%, along with the elimination product **10** as the major product (70%). Systematic screening of acid‐catalyzed conditions (Table S2) did not improve the substitution selectivity; elimination remained dominant. The predominance of elimination is probably attributable to the conformational bias of the sugar in compound **8** toward an S‐type conformation (Figure S1), which favors elimination over substitution.^[^ ^24^ ^]^

**Scheme 3:**
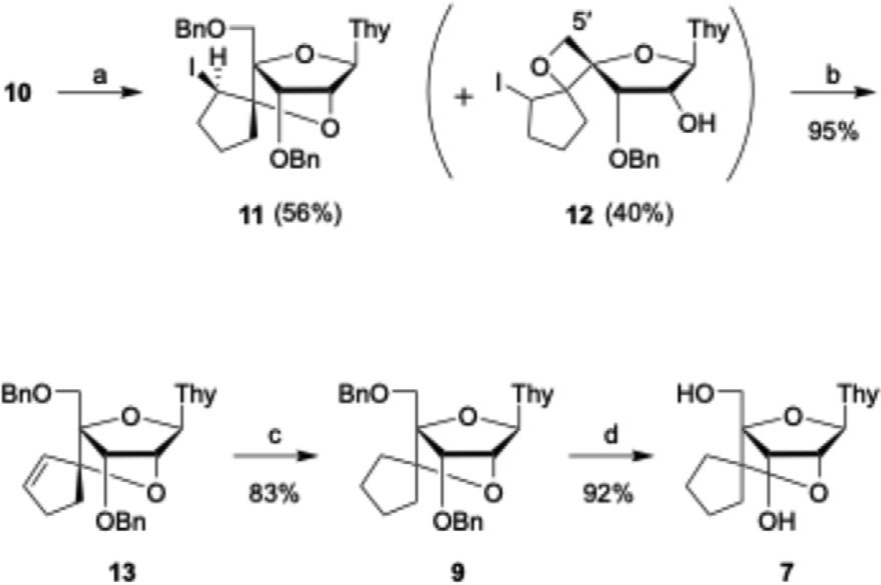
Iodocyclization‐based synthesis of scpBNA2‐T nucleoside **7**. *Reagents and conditions*: a) I_2_, NaHCO_3_, THF, 50 °C, 7 hours, 56% for **11** and 40% for **12**; b) DBU, THF, reflux, 16 hours, 95%; c) H_2_, Wilkinson's cat., THF, rt, 18 hours, 83%; d) H_2_, Pd(OH)_2_/C, AcOEt, rt, 0.5 hour, 92%.

We therefore turned to an alternative approach in which the 2′‐hydroxy group could react intramolecularly with the olefin of elimination product **10** (Scheme 3). We reasoned that electrophilic activation with iodine would generate a reactive center at the 6′‐position, enabling efficient bridge formation. Indeed, treatment of **10** with iodine and NaHCO_3_ afforded the desired bridged compound **11** as a single stereoisomer in 56% yield, along with a minor byproduct, compound **12**, also obtained as a single stereoisomer. The stereochemistry of **11** was confirmed by ^1^H NMR and NOESY spectroscopy: strong geminal coupling of the two 5′‐protons indicated magnetic nonequivalence, and a clear NOE correlation between the 1′‐proton and the proton adjacent to the iodine‐bearing carbon supported the assigned structure. Compound **12** was characterized as an oxetane derivative. Its structure was confirmed by the disappearance of the 5′‐*O*‐benzyl group, the presence of a 2′‐OH group and the absence of a 5′‐OH group, together with identification of iodine by mass spectrometry. In the ^1^H NMR spectrum, two 5′‐protons appeared as AB‐type doublets (*J* = 10 Hz), consistent with oxetane formation. The reaction likely proceeds through activation of the olefin by iodine to generate a 6′‐cation, which is intramolecularly captured by the lone pair on the 5′‐oxygen; concomitant benzyl deprotection may occur through attack of an iodide ion.

We next investigated removal of the iodine atom. E2 elimination of **11** with DBU smoothly produced **13** in 95% yield. Catalytic hydrogenation of **13** with 11 mol% Wilkinson's catalyst gave compound **9** in 83% yield. Radical reduction of **11** also provided **9** in 72% yield but generated tin‐containing byproducts (e.g., *t*‐Bu_3_SnI) that proved difficult to separate. Other methods, including catalytic hydrogenation or Zn/acid treatment, were ineffective (Table S3). Direct conversion of compound **13** into the nucleoside **7** *via* one‐step catalytic hydrogenation was also proved unsatisfactory, affording poor yields (Table S4). Ultimately, sequential hydrogenation of the olefin in **13** followed by benzyl deprotection furnished the desired nucleoside **7**.

This new synthetic route, featuring iodocyclization as a key step, eliminates the need for 2′‐OH inversion and provides a practical approach for scpBNA2 nucleoside synthesis, despite involving multiple steps with moderate yields in some transformations.

### 2.3 Extension of the Iodocyclization‐Based Strategy to Purine Derivatives

The iodocyclization‐based synthetic method, initially developed using thymine derivatives, was next applied to purine derivatives. To enable efficient access to scpBNA2‐G and scpBNA2‐A, compound **10**—positioned immediately prior to 2′,4′‐bridge formation—was selected as a common intermediate for transglycosylation.^[^ ^25^, ^26^ ^]^

**Scheme 4:**
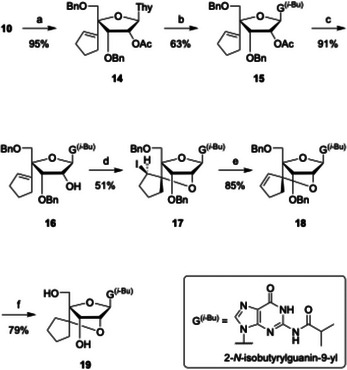
Synthesis of scpBNA2‐G nucleoside **19** *via* transglycosylation and iodocyclization*. Reagents and conditions*: a) Ac_2_O, pyridine, rt, 3 hours, 95%; b) *N* ^2^‐isobutyrylguanine, BSA, TMSOTf, MeCN, reflux, 30 hours, 63%; c) K_2_CO_3_, MeOH, –5 °C, 3 hours, 91%; d) I_2_, NaHCO_3_, THF, 50 °C, 5 hours, 51%; e) DBU, toluene, 80 °C, 20 hours, 85%; f) H_2_, Pd(OH)_2_/C, AcOEt/MeOH = 1:1, rt, 20 hours, 79%.

Acetylation of **10** afforded compound **14**, where the 2′‐acetoxy group promotes β‐selective glycosylation (Scheme 4). Heating **14** with *N* ^2^‐isobutyrylguanine, BSA, and TMSOTf for 30 hours gave compound **15** in 63% yield. Subsequent removal of the acetyl group enabled iodocyclization to furnish compound **17** as a single stereoisomer, whose configuration was confirmed by ^1^H NMR and NOESY analysis. E2 elimination of iodine from **17** produced compound **18**, which was then subjected to catalytic hydrogenation of the olefin and benzyl deprotection to afford scpBNA2‐G nucleoside **19** in 79% yield.  Notably, in contrast to the synthesis of scpBNA2‐T (Scheme 3), stepwise reduction and hydrogenolysis were not required for the preparation of **19**. Overall, scpBNA2‐G nucleoside **19** was synthesized in 14 steps with an overall yield of 4.9%, slightly higher than the 4.4% yield achieved by the 2′‐OH inversion‐based route, without an increase in step count.

**Scheme 5:**
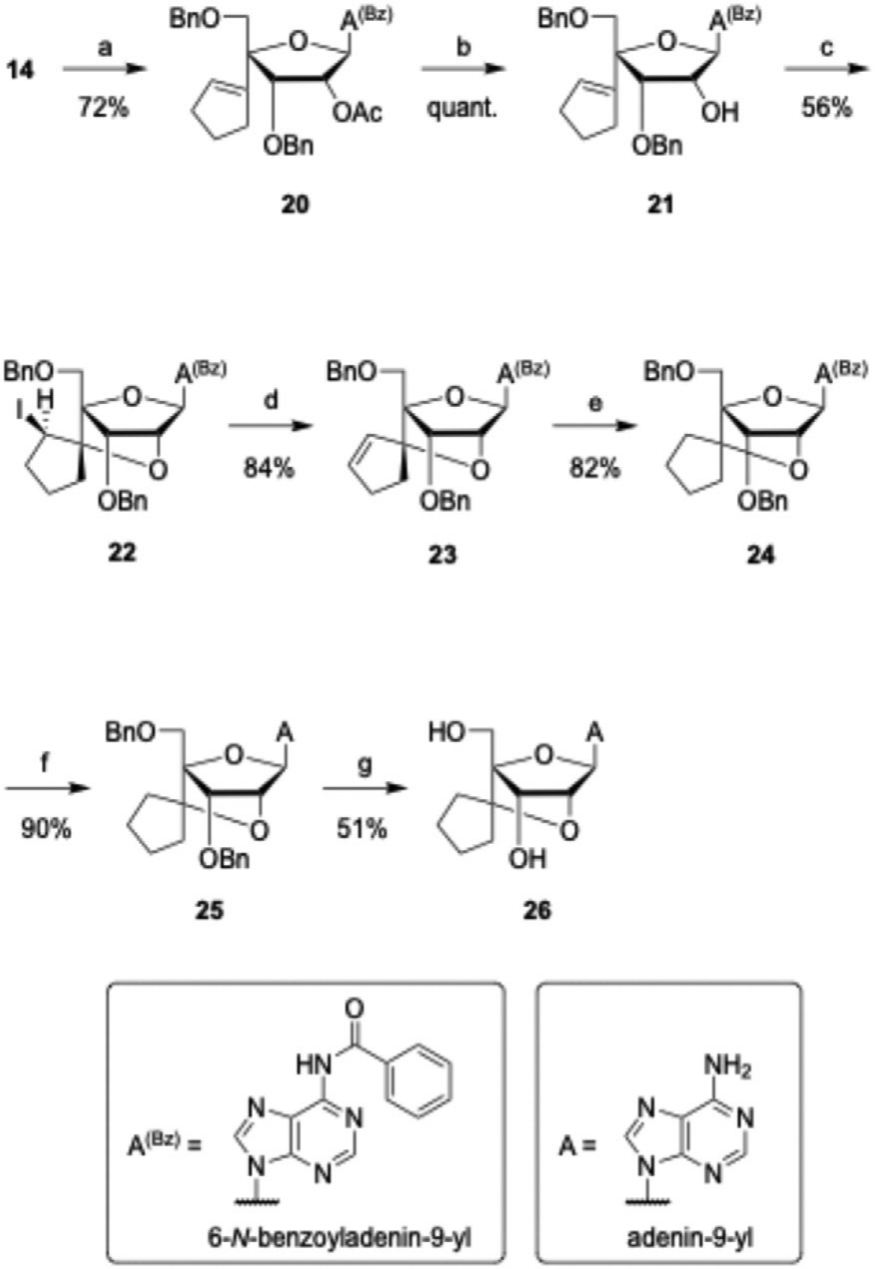
Synthesis of scpBNA2‐A nucleoside **26** *via* transglycosylation and iodocyclization. *Reagents and conditions*: a) *N* ^6^‐benzoyladenine, BSA, TMSOTf, MeCN, reflux, 14 hours, 72%; b) K_2_CO_3_, MeOH, 0 °C, 50 minutes, quant.; c) I_2_, NaHCO_3_, THF, 60 °C, 22 hours, 56%; d) DBU, THF, 60 °C, 24 hours, 84%; e) H_2_, Pd(OH)_2_/C, AcOEt, rt, 4 hours, 82%; f) MeNH_2_ aq., THF, rt, 40 minutes, 90%; g) Pd(OH)_2_/C, HCO_2_NH_4_, EtOH/AcOH = 10:1, reflux, 12 hours, 51%.

Next, the synthesis of scpBNA2‐A nucleoside **26** was pursued (Scheme 5). Using the same strategy as for the guanine analog, the 2′,4′‐bridged intermediate **23** was prepared from **14**, and subsequent olefin reduction gave compound **24**. However, benzyl deprotection by hydrogenation proved inefficient due to the known catalytic poisoning effect of *N*⁶‐benzoyladenine.^[^ ^19^, ^25^ ^]^ Attempts to remove the benzyl groups with BCl_3_ resulted in undesired depurination *via* 2′,4′‐bridge cleavage. To circumvent this, the benzoyl group of **24** was first removed with aqueous methylamine, followed by catalytic hydrogenolysis under mildly acidic conditions. Solvent composition proved critical: ethanol/acetic acid (10:1, *v*/*v*) afforded nucleoside **26** in good yield, whereas a 1:1 mixture led to complete depurination. The synthesis of scpBNA2‐A nucleoside **26** was completed in 16 steps with an overall yield of 3.2%.

Taken together, these results demonstrate that the iodocyclization‐based strategy enables the 2′‐OH inversion‐free synthesis of scpBNA2‐G and scpBNA2‐A nucleosides *via* transglycosylation.

## 3 Conclusion

Efficient access to all four nucleosides is essential for the broader application of scpBNA2 in ASO drug development. In this study, we established practical synthetic routes to scpBNA2‐T, ‐G, and ‐A nucleosides. The scpBNA2‐T nucleoside was prepared in nine steps *via* 2′,4′‐lactonization followed by 2,2′‐anhydropyrimidine formation. In addition, we developed an alternative synthetic route featuring iodocyclization as a key step, which eliminates the need for 2′‐OH inversion. Notably, scpBNA2‐T nucleoside also serves as an intermediate for the preparation of scpBNA2‐^m^C nucleoside. For purine nucleosides, the iodocyclization‐based approach, combined with late‐stage transglycosylation, enabled efficient synthesis of scpBNA2‐G and scpBNA2‐A nucleosides.

## 4 Experimental Section

### General experimental procedure

Reagents and solvents were purchased from commercial suppliers and used without further purification. All experiments involving air‐ and/or moisture‐sensitive reagents were conducted under a nitrogen or argon atmosphere. Reaction progress was monitored by analytical thin‐layer chromatography (Merck Kieselgel 60 F254; Merck). Column chromatography was performed on silica gel (PSQ‐100B or PSQ‐60B; Fuji Silysia Chemical) or on amino‐modified silica gel (CHROMATOREX NH‐DM2035; Fuji Silysia Chemical). Automated flash column chromatography was carried out using an EPCLC‐W‐Prep 2XY system (Yamazen). LC–MS analyses were performed on a Waters ACQUITY UPLC H‐Class PLUS System equipped with a TUV Detector and an ACQUITY RDa Detector. NMR spectra were recorded on JEOL JNM‐AL300, JNM‐ECS400, JNM‐ECS500, and BRUKER Ascend 400 spectrometers in CDCl_3_, DMSO‐*d*_6_, or MeOH‐*d*_4_, with tetramethylsilane (0.00 ppm) as an internal reference. IR spectra were recorded on a FT/IR‐4200 spectrophotometer (JASCO). MALDI‐TOF mass spectra of new compounds were acquired using a JEOL SpiralTOF JMS‐S3000 instrument.

### Compound **2**

To a solution of compound **1** (1.00 g, 2.13 mmol) in dry dichloromethane (16 mL) were added iodobenzene diacetate (3.63 g, 11.3 mmol) and 2,2,6,6‐tetramethylpiperidine 1‐oxyl free radical (108 mg, 691 µmol) at 0 °C, and the reaction mixture was stirred at room temperature for 18 hours under N_2_ atmosphere. After the reaction was completed, saturated aqueous Na_2_S_2_O_3_ was added, and the mixture was extracted with Et_2_O. The combined organic layer was washed with saturated aqueous NaHCO_3_ and brine, dried over Na_2_SO_4_, and concentrated. The crude product was purified by column chromatography (SiO_2_, hexane/AcOEt = 1:1) to afford **2** (709 mg, 72%) as a white foam. ^1^H NMR (500 MHz, CDCl_3_) *δ* 1.60 (s, 3H), 3.97, 4.10 (AB, *J* = 11.5 Hz, 2H), 4.29 (s, 1H), 4.61, 4.69 (AB, *J* = 12.0 Hz, 2H), 4.62, 4.66 (AB, *J* = 11.5 Hz, 2H), 5.24 (s, 1H), 5.68 (s, 1H), 7.24–7.38 (m, 10H), 7.48 (s, 1H), 9.92 (brs, 1H); ^13^C NMR (125.7 MHz, CDCl_3_) *δ* 12.3, 62.4, 72.6, 74.1, 78.9, 83.7, 84.8, 111.0, 127.7, 127.8, 128.2, 128.4, 128.5, 128.6, 134.5, 135.9, 137.1, 150.0, 164.0, 169.7; IR (KBr): 3157, 3033, 2929, 2877, 2830, 1810, 1698, 1497, 1457, 1389, 1365, 1334, 1275, 1192, 1154, 1115, 1052, 1025, 1000, 947, 934, 916, 876, 835, 791, 777, 762, 739, 721, 697, 607, 580 cm^−1^; HRMS (MALDI) Calcd. for C_25_H_24_N_2_O_7_Na [M + Na]^+^ 487.1476, found 487.1472.

### Compound **3**

To a solution of **2** (3.10 g, 6.67 mmol) in dry THF (50 mL) was added 1.0 M allylmagnesium bromide in Et_2_O (15.0 mL, 15.0 mmol) at –35 °C, and the mixture was stirred at the same temperature for 1 hour under N_2_ atmosphere. After the completion of the reaction, saturated aqueous NH_4_Cl was added, and the product was extracted with AcOEt. The organic layer was washed with brine and dried over Na_2_SO_4_. The organic layer was concentrated under reduced pressure and purified by column chromatography (SiO_2_, hexane/AcOEt = 11:9) to afford **3** (3.00 g, 82%) as a white form. ^1^H NMR (500 MHz, CDCl_3_) *δ* 1.55 (s, 3H), 2.31–2.35 (m, 1H), 2.38–2.43 (m, 1H), 2.60–2.66 (m, 2H), 3.41 (brs, 1H), 3.68 (d, *J* = 10.5 Hz, 1H), 3.71 (d, *J* = 11.0 Hz, 1H), 3.98 (brs, 1H), 4.37–4.44 (m, 2H), 4.48–4.54 (m, 2H), 4.54–4.56 (m, 1H), 4.93–5.08 (m, 5H), 5.79–5.92 (m, 2H), 6.11 (d, *J* = 5.0 Hz, 1H), 7.25–7.38 (m, 11H), 8.77 (brs, 1H); ^13^C NMR (75.6 MHz, CDCl_3_) *δ* 12.3, 40.3, 40.9, 72.2, 73.9, 75.0, 75.8, 77.8, 81.2, 89.6, 91.2, 111.3, 118.2, 118.3, 127.7, 128.2, 128.4, 128.6, 128.7, 128.8, 134.0, 134.2, 136.0, 136.9, 137.3, 151.2, 164.1; HRMS (MALDI) Calcd. for C_31_H_36_N_2_O_7_Na [M + Na]^+^ 571.2415, found 571.2404.

### Compound **4**

To a solution of **3** (760 mg, 1.39 mmol) in dry and deoxygenated CH_2_Cl_2_ (35 mL) was added Grubbs 2^nd^ generation catalyst (117.6 mg, 138.6 µmol, 0.10 eq.) at room temperature, and the reaction mixture was refluxed for 8 hours under N_2_ atmosphere. After the completion of the reaction, the mixture was concentrated. The crude product was purified by column chromatography (SiO_2_, hexane/AcOEt = 1:1→9:11) to afford **4** (540 mg, 75%) as a gray form. ^1^H NMR (300 MHz, CDCl_3_) *δ* 1.57 (d, *J* = 1.2 Hz, 3H), 2.31–2.48 (m, 2H), 2.67–2.82 (m, 2H), 2.94 (brs, 1H, OH), 3.58 (d, *J* = 9.3 Hz, 1H, OH), 3.67 (d, *J* = 10.2 Hz, 1H), 3.75 (d, *J* = 9.9 Hz, 1H), 4.39–4.47 (m, 2H), 4.54 (d, *J* = 11.7 Hz, 1H), 5.58 (d, *J* = 11.4 Hz, 1H), 4.67 (d, *J* = 11.1 Hz, 1H), 4.91 (d, *J* = 10.5 Hz, 1H), 5.58–5.65 (m, 2H), 6.12 (d, *J* = 6.6 Hz, 1H), 7.30–7.42 (m, 11H), 8.75 (brs, 1H); ^13^C NMR (75.6 MHz, CDCl_3_) *δ* 12.2, 44.9, 73.2, 73.9, 75.3, 75.9, 80.6, 84.3, 88.8, 90.7, 111.4, 127.4, 127.6, 128.1, 128.4, 128.5, 128.6, 128.7, 128.8, 136.0, 137.1, 137.4, 151.3, 164.1; HRMS (MALDI) Calcd. for C_29_H_32_N_2_O_7_Na [M + Na]^+^ 543.2102, found 543.2094.

### Compound **5**

To a solution of a mixture of **4** (90.0 mg, 173 µmol) in dry CH_2_Cl_2_ (2 mL) were added dry pyridine (140 µL, 1.74 mmol) and methanesulfonyl chloride (16.1 µL, 208 µmol) at 0 °C, and the reaction mixture was stirred at room temperature for 2 hours under N_2_ atmosphere. After the completion of the reaction, saturated aqueous NaHCO_3_ was added, and the product was extracted with AcOEt. The organic layer was washed with brine and dried over Na_2_SO_4_ and concentrated under reduced pressure. The residue was purified by column chromatography (SiO_2_, hexane/AcOEt = 3:2→1:1) to afford **5** (92.1 mg, 89%) as a white form. ^1^H NMR (400 MHz, CDCl_3_) *δ* 1.54 (s, 3H), 2.27–2.31 (m, 1H), 2.39–2.44 (m, 1H), 2.62–2.67 (m, 1H), 2.73–2.78 (m, 2H), 3.02 (s, 3H), 3.69 (d, *J* = 10.0 Hz, 1H), 3.72 (d, *J* = 10.0 Hz, 1H), 5.54 (d, *J* = 5.6 Hz, 1H), 4.55 (d, *J* = 12.0 Hz, 1H), 4.57 (d, *J* = 11.2 Hz, 1H), 4.60 (d, *J* = 11.6 Hz, 1H), 4.96 (d, *J* = 11.2 Hz, 1H), 5.42 (dd, *J* = 6.4, 6.8 Hz, 1H), 5.56–5.63 (m, 2H), 6.40 (d, *J* = 7.2 Hz, 1H), 7.33–7.43 (m, 11H), 9.16 (brs, 1H); ^13^C NMR (100.6 MHz, CDCl_3_) *δ* 12.2, 38.6, 44.9, 72.6, 74.0, 75.5, 78.2, 79.6, 83.9, 85.9, 91.3, 112.2, 127.2, 127.8, 128.5, 128.5, 128.7, 128.8, 128.9, 129.0, 135.2, 136.4, 137.0, 150.9, 163.7; HRMS (MALDI) Calcd. for C_30_H_34_N_2_O_9_NaS [M + Na]^+^ 621.1877, found 525.1987. Since this compound was highly unstable, it is likely that the 2,2′‐anhydro compound was formed during the preparation of the sample (Matrix: CHCA or DCTB).

### Compound **6**

To a solution of **5** (10.1 mg, 16.9 µmol) in dry DMF (1.5 mL) was added potassium carbonate (7.0 mg, 51 µmol) at room temperature, and the reaction mixture was stirred at 90 °C for 6 hours. After the completion of the reaction, saturated aqueous NaHCO_3_ was added, and the product was extracted with hexane/AcOEt (3:1). The organic layer was washed with brine and dried over Na_2_SO_4_. The organic layer was concentrated under reduced pressure and purified by column chromatography (SiO_2_, hexane/AcOEt = 3:2) to afford **6** (7.7 mg, 91%) as a white form. The NMR spectral data of compound **6** was identical to those reported in the literature.^[^ ^22^ ^]^

### Compound **7**

To a solution of **6** (22.9 mg, 45.6 µmol) in AcOEt (2 mL) was added palladium hydroxide 20% on carbon (6.9 mg, 30wt%), and the mixture was stirred at room temperature for 50 minutes under H_2_ atmosphere. After completion of the reaction, the mixture was filtered through Celite and the Celite pad was washed with AcOEt and MeOH. The filtrate was concentrated, and the crude product was purified by column chromatography (SiO_2_, CHCl_3_/MeOH = 20:1→10:1) to afford **7** (13.6 mg, 92%) as a white solid. The NMR spectral data of compound **7** was identical to those reported in the literature.^[^ ^22^ ^]^

### Compound **8**

To a solution of **4** (6.30 g, 12.1 mmol) in dry THF (200 mL) was added Wilkinson's catalyst (chlorotris(triphenylphosphine)rhodium(I), 1.12 g, 1.21 mmol, 0.10 eq.) at room temperature, and the reaction mixture was stirred at the same temperature for 20 hours under H_2_ atmosphere. After the completion of the reaction, the mixture was concentrated. The crude product was purified by column chromatography (SiO_2_, hexane/AcOEt = 1:1) to afford **8** (6.06 g, 96%) as a light‐yellow form. ^1^H NMR (300 MHz, CDCl_3_) *δ* 1.47–2.11 (m, 8H), 1.56 (d, *J* = 1.2 Hz, 3H), 2.77 (brs, 1H), 3.68 (d, *J* = 9.9 Hz, 1H), 3.81 (d, *J* = 9.6 Hz, 1H), 4.23 (d, *J* = 9.6 Hz, 1H), 4.38–4.47 (m, 1H), 4.40 (brs, 1H), 4.56 (s, 2H), 4.59 (d, *J* = 11.4 Hz, 1H), 5.07 (d, *J* = 11.4 Hz, 1H), 6.18 (d, *J* = 6.6 Hz, 1H), 7.30–7.42 (m, 11H), 9.67 (brs, 1H); ^13^C NMR (75.6 MHz, CDCl_3_) *δ* 12.3, 23.6, 24.1, 36.5, 37.4, 73.3, 74.0, 75.3, 75.9, 80.7, 85.4, 89.1, 90.4, 111.5, 127.7, 128.2, 128.5, 128.5, 128.8, 128.9, 136.0, 137.2, 137.5, 151.4, 164.1; HRMS (MALDI) Calcd. for C_29_H_34_N_2_O_7_Na [M + Na]^+^ 545.2258, found 545.2250.

### Compounds **9** and **10**

To a solution of **8** (650 mg, 1.24 mmol) in acetonitrile (20 mL) was added *p*‐toluenesulfonic acid monohydrate (119 mg, 626 µmol), and the mixture was stirred at 50 °C for 13 hours under N_2_ atmosphere. After the completion of the reaction, saturated aqueous NaHCO_3_ was added, and the product was extracted with AcOEt. The organic layer was washed with brine and dried over Na_2_SO_4_. The organic layer was concentrated under reduced pressure and purified by column chromatography (SiO_2_, hexane/AcOEt = 11:9) to afford **9** (22.9 mg, 4%) as a white form and **10** (441 mg, 70%) as a white form. **9**: ^1^H NMR (500 MHz, CDCl_3_) *δ* 1.39–1.47 (m, 1H), 1.54–1.61 (m, 1H), 1.57 (d, *J* = 1.0 Hz, 3H), 1.68–1.73 (m, 1H), 1.77–1.89 (m, 3H), 1.92–2.00 (m, 2H), 3.83 (d, *J* = 10.5 Hz, 1H), 3.90 (d, *J* = 11.0 Hz, 1H), 4.00 (s, 1H), 4.45 (s, 1H), 4.50 (d, *J* = 11.5 Hz, 1H), 4.60 (d, *J* = 11.5 Hz, 1H), 4.63 (d, *J* = 10.5 Hz, 1H), 4.64 (d, *J* = 11.5 Hz, 1H), 5.55 (s, 1H), 7.27–7.38 (m, 10H), 7.53 (d, *J* = 1.0 Hz, 1H), 8.44 (brs, 1H); ^13^C NMR (125.8 MHz, CDCl_3_) δ 12.4, 23.6, 25.7, 35.7, 35.8, 65.0, 72.3, 74.1, 78.0, 86.6, 89.0, 94.6, 110.1, 127.8, 127.9, 128.1, 128.2, 128.6, 128.7, 135.3, 137.1, 137.7, 149.8, 163.7; HRMS (MALDI) Calcd. for C_29_H_32_N_2_O_6_Na [M + Na]^+^ 527.2153, found 527.2150. **10**: ^1^H NMR (500 MHz, CDCl_3_) *δ* 1.54 (s, 3H), 1.78–1.89 (m, 3H), 2.35–2.52 (m, 4H), 3.02 (s, 1H), 3.64 (d, *J* = 10.0 Hz, 1H), 3.81 (d, *J* = 10.0 Hz, 1H), 4.19 (d, *J* = 6.0 Hz, 1H), 4.38–4.42 (m, 1H), 4.56 (d, *J* = 11.0 Hz, 1H), 4.60 (d, *J* = 11.0 Hz, 1H), 4.63 (s, 2H), 5.72–5.72 (m, 1H), 6.07 (d, *J* = 6.5 Hz, 1H), 7.28–7.38 (m, 10H), 7.54 (d, *J* = 1.5 Hz, 1H), 8.65 (brs, 1H); ^13^C NMR (75.6 MHz, CDCl_3_) *δ* 12.1, 23.3, 32.7, 33.4, 73.8, 74.1, 74.7, 74.9, 81.3, 88.0, 88.5, 111.2, 127.3, 127.7, 127.9, 128.2, 128.3, 128.5, 128.6, 128.8, 135.9, 137.3, 142.6, 151.1, 163.8; HRMS (MALDI) Calcd. for C_29_H_32_N_2_O_6_Na [M + Na]^+^ 527.2153, found 527.2149.

### Compounds **11** and **12**

To a solution of **10** (360 mg, 713 µmol) in THF (10 mL) were added sodium hydrogen carbonate (153 mg, 1.82 mmol) and iodine (360 mg, 1.42 mmol) at room temperature, and the mixture was stirred at 50 °C for 7 hours under N_2_ atmosphere. After the completion of the reaction, saturated aqueous Na_2_S_2_O_3_ was added, and the product was extracted with AcOEt. The organic layer was washed with brine and dried over Na_2_SO_4_. The organic layer was concentrated under reduced pressure and purified by column chromatography (SiO_2_, hexane/AcOEt = 3:2) to afford **11** (254 mg, 56%) as a white form and **12** (155 mg, 40%) as a white form. **11**: ^1^H NMR (500 MHz, CDCl_3_) *δ* 1.53 (d, *J* = 1.0 Hz, 3H), 1.86–1.93 (m, 1H), 1.98–2.07 (m, 1H), 2.09–2.20 (m, 2H), 2.22–2.27 (m, 1H), 2.52–2.59 (m, 1H), 3.90 (d, *J* = 10.5 Hz, 1H), 4.16 (s, 1H), 4.29–4.30 (m, 1H), 4.37 (s, 1H), 4.54 (d, *J* = 12.0 Hz, 1H), 4.62 (d, *J* = 3.0 Hz, 1H), 4.64 (d, *J* = 3.5 Hz, 1H), 4.68 (d, *J* = 10.5 Hz, 1H), 4.89 (d, *J* = 10.5 Hz, 1H), 5.61 (s, 1H), 7.24–7.38 (m, 10H), 7.56 (d, *J* = 1.0 Hz, 1H), 8.40 (brs, 1H); ^13^C NMR (125.8 MHz, CDCl_3_) *δ* 12.3, 21.7, 29.9, 34.8, 36.3, 66.1, 72.6, 74.0, 76.9, 77.1, 79.0, 86.4, 89.7, 97.4, 110.3, 127.8, 127.9, 128.2, 128.2, 128.6, 128.7, 135.2, 136.9, 137.7, 150.0, 164.0; HRMS (MALDI) Calcd. for C_29_H_31_N_2_O_6_NaI [M + Na]^+^ 653.1119, found 653.1119. **12**: ^1^H NMR (500 MHz, CDCl_3_) *δ* 1.50 (s, 3H), 1.93–2.14 (m, 5H), 2.24–2.29 (m, 1H), 2.98 (brs, 1H→signal disappeared by a D_2_O drop), 3.79 (d, *J* = 10.0 Hz, 1H), 4.01 (d, *J* = 10.5 Hz, 1H), 4.31–4.34 (m, 1H→dd, *J* = 4.5, 8.0 Hz, 1H by a D_2_O drop), 4.61 (d, *J* = 4.5 Hz, 1H), 4.63 (d, *J* = 11.0 Hz, 1H), 4.66 (d, *J* = 11.5 Hz, 1H), 4.71 (d, *J* = 4.5 Hz, 1H), 6.00 (d, *J* = 8.0 Hz, 1H), 7.32–7.40 (m, 5H), 7.54 (d, *J* = 1.0 Hz, 1H), 8.39 (brs, 1H→signal disappeared by a D_2_O drop); ^13^C NMR (75.6 MHz, CDCl_3_) *δ* 12.0, 24.3, 28.6, 40.8, 56.5, 71.6, 74.0, 77.4, 80.2, 87.5, 93.1, 94.7, 111.9, 127.5, 128.4, 128.9, 135.6, 136.9, 151.4, 163.8; HRMS (MALDI) Calcd. for C_22_H_25_N_2_O_6_NaI [M + Na]^+^ 563.0650, found 563.0648.

### Compound **13**

To a solution of **11** (50.0 mg, 79.3 µmol) in dry THF (1 mL) was added DBU (23.6 µL, 159 µmol) at room temperature, and the mixture was refluxed for 16 hours under N_2_ atmosphere. After the completion of the reaction, water was added, and the product was extracted with AcOEt. The organic layer was washed with brine and dried over Na_2_SO_4_. The organic layer was concentrated under reduced pressure and purified by column chromatography (SiO_2_, hexane/AcOEt = 3:2→1:1) to afford **13** (37.8 mg, 95%) as a white form. ^1^H NMR (500 MHz, CDCl_3_) *δ* 1.56 (d, *J* = 1.5 Hz, 3H), 2.00–2.06 (m, 1H), 2.17–2.24 (m, 1H), 2.32–2.37 (m, 1H), 2.47–2.54 (m, 1H), 3.71 (d, *J* = 11.0 Hz, 1H), 3.90 (d, *J* = 11.5 Hz, 1H), 4.07 (s, 1H), 4.53 (d, *J* = 11.0 Hz, 1H), 4.56 (s, 1H), 4.58 (d, *J* = 11.5 Hz, 1H), 4.61 (d, *J* = 11.5 Hz, 1H), 4.67 (d, *J* = 12.0 Hz, 1H), 5.61 (s, 1H), 5.81–5.83 (m, 1H), 6.12–6.14 (m, 1H), 7.28–7.37 (m, 10H), 7.57 (d, *J* = 1.0 Hz, 1H), 9.05 (brs, 1H); ^13^C NMR (125.8 MHz, CDCl_3_) *δ* 12.3, 31.7, 33.0, 64.8, 72.4, 74.1, 77.3, 77.6, 86.7, 89.0, 98.1, 110.1, 127.8, 127.9, 128.1, 128.2, 128.6, 128.7, 131.0, 135.3, 137.1, 137.6, 137.7, 150.0, 164.1; HRMS (MALDI) Calcd. for C_29_H_30_N_2_O_6_Na [M + Na]^+^ 525.1996, found 525.1997.

### Compound **9** from compound **13**

To a solution of **13** (17.5 mg, 34.8 µmol) in dry THF (1 mL) was added Wilkinson's catalyst (chlorotris(triphenylphosphine)rhodium(I), 3.4 mg, 3.7 µmol, 0.11 eq.) at room temperature, and the reaction mixture was stirred at the same temperature for 18 hours under H_2_ atmosphere. After the completion of the reaction, the mixture was concentrated. The crude product was purified by column chromatography (SiO_2_, hexane/AcOEt = 6:4→9:11) to afford **9** (14.5 mg, 83%) as a light‐yellow form.

### Compound **7** from compound **9**

To a solution of **9** (21.0 mg, 41.6 µmol) in AcOEt (1 mL) was added palladium hydroxide 20% on carbon (9.4 mg, 45wt%), and the mixture was stirred at room temperature for 30 minutes under H_2_ atmosphere. After completion of the reaction, the mixture was filtered through Celite and the Celite pad was washed with AcOEt and MeOH. The filtrate was concentrated, and the crude product was purified by column chromatography (SiO_2_, CHCl_3_/MeOH = 20:1→10:1) to afford **7** (12.4 mg, 92%) as a white solid.

### Compound **14**

To a solution of **10** (408 mg, 809 µmol) in dry pyridine (8 mL) was added acetic anhydride (620 µL, 6.56 mmol) at 0 °C, and the mixture was stirred at room temperature for 3 hours under N_2_ atmosphere. After the completion of the reaction, water was added, and the product was extracted with CHCl_3_. The organic layer was washed with brine and dried over Na_2_SO_4_. The organic layer was concentrated under reduced pressure and purified by column chromatography (SiO_2_, hexane/AcOEt = 1:1) to afford **14** (420 mg, 95%) as a white form. ^1^H NMR (300 MHz, CDCl_3_) *δ* 1.51 (d, *J* = 0.9 Hz, 3H), 1.76–1.86 (m, 2H), 2.04 (s, 3H), 2.31–2.47 (m, 4H), 3.64 (d, *J* = 10.5 Hz, 1H), 3.75 (d, *J* = 9.9 Hz, 1H), 4.41 (d, *J* = 5.4 Hz, 1H), 4.47 (d, *J* = 11.7 Hz, 1H), 4.54 (d, *J* = 11.1 Hz, 1H), 4.57 (d, *J* = 11.1 Hz, 1H), 4.59 (d, *J* = 11.4 Hz, 1H), 5.39 (dd, *J* = 5.4, 6.3 Hz, 1H), 5.70–5.71 (m, 1H), 6.35 (d, *J* = 6.3 Hz, 1H), 7.25–7.39 (m, 10H), 7.59 (d, *J* = 1.2 Hz, 1H), 8.78 (brs, 1H); ^13^C NMR (75.6 MHz, CDCl_3_) *δ* 12.1, 20.8, 23.6, 32.6, 33.4, 73.6, 73.8, 74.6, 75.2, 79.8, 85.8, 88.5, 111.5, 127.5, 127.7, 127.7, 128.0, 128.3, 128.5, 128.8, 135.8, 137.3, 137.8, 142.1, 150.7, 163.8, 170.4; HRMS (MALDI) Calcd. for C_31_H_34_N_2_O_7_Na [M + Na]^+^ 569.2258, found 569.2260.

### Compound **15**

To a solution of **14** (570 mg, 1.04 mmol) in dry acetonitrile (20 mL) were added *N* ^2^‐isobutyrylguanine (700 mg, 3.16 mmol) and *N*,*O*‐bis(trimethylsilyl)acetamide (1.53 mL, 6.26 mmol) at 0 °C under N_2_ atmosphere. The suspension was refluxed until all the substrates were dissolved, and then the resulting solution was cooled to 0 °C. TMSOTf (283 µL, 1.57 mmol) was added, and the reaction mixture was refluxed for 30 hours. After the reaction was completed, saturated aqueous NaHCO_3_ was added, and the resulting mixture was extracted with AcOEt. The combined organic layer was washed with water and brine, dried over Na_2_SO_4_, and concentrated. To the crude product was added a small amount of AcOEt, and the mixture was stirred at room temperature for 3 minutes. Then, the mixture was filtered through Celite and the Celite pad was washed with AcOEt. The filtrate was concentrated, and the crude product was purified first by column chromatography (SiO_2_, hexane/AcOEt = 1:1→1:5) and second by column chromatography (SiO_2_, CHCl_3_/MeOH = 99:1) twice to afford **15** (420 mg, 63%) as a white foam. ^1^H NMR (500 MHz, CDCl_3_) *δ* 1.22 (d, *J* = 7.0 Hz, 3H), 1.22 (d, *J* = 7.0 Hz, 3H), 1.78–1.85 (m, 2H), 1.98 (s, 3H), 2.32–2.48 (m, 4H), 2.53–2.58 (m, 1H), 3.56 (d, *J* = 10.0 Hz, 1H), 3.73 (d, *J* = 10.0 Hz, 1H), 4.47 (d, *J* = 11.5 Hz, 1H), 4.50 (d, *J* = 11.5 Hz, 1H), 4.53 (d, *J* = 11.5 Hz, 1H), 4.55 (d, *J* = 6.0 Hz, 1H), 4.58 (d, *J* = 12.0 Hz, 1H), 5.71 (dd, *J* = 5.5, 5.5 Hz, 1H), 5.71–5.72 (m, 1H), 6.14 (d, *J* = 6.0 Hz), 7.23–7.25 (m, 2H), 7.28–7.37 (m, 8H), 7.96 (s, 1H), 8.43 (brs, 1H), 11.97 (brs, 1H); ^13^C NMR (125.8 MHz, CDCl_3_) *δ* 19.0, 19.1, 20.7, 23.6, 32.6, 33.4, 36.6, 73.2, 73.8, 74.5, 76.0, 79.9, 85.2, 89.2, 121.4, 127.5, 127.8, 127.9, 128.0, 128.2, 128.5, 128.8, 137.3, 137.5, 137.8, 142.2, 147.5, 148.4, 155.6, 170.0, 178.3; HRMS (MALDI) Calcd. for C_35_H_39_N_5_O_7_Na [M + Na]^+^ 664.2742, found 664.2750.

### Compound **16**

To a solution of **15** (410 mg, 639 µmol) in methanol (10 mL) was added potassium carbonate (96.0 mg, 695 µmol) at –5 °C, and the reaction mixture was stirred at the same temperature for 3 hours. After the completion of the reaction, saturated aqueous NaHCO_3_ was added, and the product was extracted with AcOEt. The organic layer was washed with brine and dried over Na_2_SO_4_. The organic layer was concentrated under reduced pressure and purified first by column chromatography (SiO_2_, hexane/AcOEt = 1:5) and second by column chromatography (SiO_2_, CHCl_3_/MeOH = 50:1) to afford **16** (349 mg, 91%) as a white form. ^1^H NMR (300 MHz, CDCl_3_) *δ* 1.26 (d, *J* = 6.9 Hz, 3H), 1.26 (d, *J* = 6.9 Hz, 3H), 1.84–1.93 (m, 2H), 2.37–2.59 (m, 5H), 3.05 (d, *J* = 8.7 Hz, 1H, OH), 3.58 (d, *J* = 10.2 Hz, 1H), 3.76 (d, *J* = 10.2 Hz, 1H), 4.34 (d, *J* = 5.4 Hz, 1H), 4.52 (d, *J* = 12.0 Hz, 1H), 4.59 (d, *J* = 12.0 Hz, 1H), 4.57–4.66 (m, 3H), 5.79–5.80 (m, 1H), 5.94 (d, *J* = 5.4 Hz, 1H), 7.27–7.41 (m, 10H), 8.00 (s, 1H), 8.13 (brs, 1H), 11.89 (brs, 1H); ^13^C NMR (75.6 MHz, CDCl_3_) δ 19.0, 19.0, 23.5, 32.6, 33.4, 36.3, 73.6, 73.7, 74.2, 76.0, 81.0, 89.1, 89.3, 120.9, 127.5, 127.7, 127.8, 127.9, 128.0, 128.4, 128.6, 137.4, 137.7, 137.7, 142.6, 147.7, 148.5, 155.9, 179.1; HRMS (MALDI) Calcd. for C_33_H_37_N_5_O_6_Na [M + Na]^+^ 622.2636, found 622.2636.

### Compound **17**

To a solution of **16** (10.0 mg, 16.7 µmol) in THF (50 µL) were added sodium hydrogen carbonate (3.7 mg, 44.0 mmol) and iodine (8.3 mg, 33 µmol) in THF (150 µL) at room temperature, and the mixture was stirred at 50 °C for 5 hours under N_2_ atmosphere. After the completion of the reaction, saturated aqueous Na_2_S_2_O_3_ was added, and the product was extracted with AcOEt. The organic layer was washed with brine and dried over Na_2_SO_4_. The organic layer was concentrated under reduced pressure and purified by column chromatography (SiO_2_, hexane/AcOEt = 1:1) to afford **17** (6.2 mg, 51%) as a white form. ^1^H NMR (300 MHz, CDCl_3_) *δ* 1.22 (d, *J* = 6.9 Hz, 3H), 1.24 (d, *J* = 6.9 Hz, 3H), 1.81–1.89 (m, 1H), 2.00–2.28 (m, 4H), 2.46–2.55 (m, 1H), 2.69–2.79 (m, 1H), 3.84 (d, *J* = 11.4 Hz, 1H), 4.23 (d, *J* = 4.5 Hz, 1H), 4.28 (s, 1H), 4.44 (s, 1H), 4.51 (s, 2H), 4.57 (d, *J* = 12.0 Hz, 1H), 4.69 (d, *J* = 12.3 Hz, 1H), 4.73 (d, *J* = 12.9 Hz, 1H), 5.77 (s, 1H), 7.15–7.34 (m, 10H), 7.90 (s, 1H), 9.50 (brs, 1H), 12.15 (brs, 1H); ^13^C NMR (75.6 MHz, CDCl_3_) *δ* 19.1, 21.7, 29.9, 34.3, 36.3, 36.4, 65.6, 72.7, 73.8, 77.6, 80.0, 85.5, 89.4, 97.8, 121.5, 127.6, 127.8, 128.0, 128.0, 128.5, 128.7, 136.3, 137.0, 137.6, 147.2, 147.9, 155.7, 179.1; HRMS (MALDI) Calcd. for C_33_H_36_N_5_O_6_NaI [M + Na]^+^ 748.1602, found 748.1603.

### Compound **18**

To a solution of **17** (190 mg, 262 µmol) in dry toluene (2 mL) was added DBU (78.3 µL, 525 µmol) at the room temperature, and the mixture was stirred at 80 °C for 20 hours under N_2_ atmosphere. After completion of the reaction, water was added, and the product was extracted with AcOEt. The organic layer was washed with brine and dried over Na_2_SO_4_. The organic layer was concentrated under reduced pressure and purified by column chromatography (SiO_2_, hexane/AcOEt/MeOH = 15:10:1) to afford **18** (133 mg, 85%) as a white form. ^1^H NMR (500 MHz, CDCl_3_) *δ* 1.29 (d, *J* = 7.0 Hz, 3H), 1.29 (d, *J* = 6.5 Hz, 3H), 1.98–2.06 (m, 1H), 2.19–2.25 (m, 1H), 2.35–2.40 (m, 1H), 2.47–2.53 (m, 1H), 2.63–2.69 (m, 1H), 3.69 (d, *J* = 11.0 Hz, 1H), 3.85 (d, *J* = 11.5 Hz, 1H), 4.34 (s, 1H), 4.48 (s, 1H), 4.52 (d, *J* = 11.0 Hz, 1H), 4.56 (d, *J* = 10.5 Hz, 1H), 4.59 (d, *J* = 12.5 Hz, 1H), 4.66 (d, *J* = 12.5 Hz, 1H), 5.76 (s, 1H), 5.82–5.84 (m, 1H), 6.12–6.14 (m, 1H), 7.24–7.38 (m, 10H), 7.92 (s, 1H), 8.62 (brs, 1H), 12.03 (brs, 1H); ^13^C NMR (125.8 MHz, CDCl_3_) *δ* 19.1, 31.7, 33.2, 36.7, 64.4, 72.7, 73.9, 77.9, 78.9, 85.8, 88.7, 98.6, 121.9, 127.6, 127.7, 128.1, 128.1, 128.6, 128.7, 131.1, 136.4, 137.2, 137.6, 137.7, 147.1, 147.6, 155.6, 178.4; HRMS (MALDI) Calcd. for C_33_H_35_N_5_O_6_Na [M + Na]^+^ 620.2480, found 620.2480.

### Compound **19**

To a solution of **18** (30.0 mg, 50.2 µmol) in AcOEt/MeOH (1 mL, 1:1) was added palladium hydroxide 20% on carbon (15.0 mg, 50wt%), and the mixture was stirred at room temperature for 20 hours under H_2_ atmosphere. After completion of the reaction, the mixture was filtered through Celite and the Celite pad was washed with AcOEt and MeOH. The filtrate was concentrated, and the crude product was purified by column chromatography (SiO_2_, CHCl_3_/MeOH = 10:1→5:1) and column chromatography (SiO_2_‐NH_2_, CHCl_3_/MeOH = 15:1→10:1) to afford **19** (16.0 mg, 79%) as a white solid. ^1^H NMR (300 MHz, CD_3_OD) *δ* 1.22 (d, *J* = 6.9 Hz, 3H), 1.22 (d, *J* = 6.9 Hz, 3H), 1.48–2.02 (m, 7H), 2.09–2.20 (m, 1H), 3.35 (s, 1H), 3.92 (d, *J* = 12.9 Hz, 1H), 3.99 (d, *J* = 12.6 Hz, 1H), 4.38 (s, 1H), 4.43 (s, 1H), 5.80 (s, 1H), 8.11 (s, 1H); ^13^C NMR (125.8 MHz, CD_3_OD) *δ* 19.3, 19.3, 24.4, 26.5, 36.4, 37.0, 37.0, 57.8, 73.3, 81.6, 86.9, 91.1, 96.0, 121.5, 138.4, 149.4, 149.9, 157.4, 181.7; HRMS (MALDI) calcd. for C_19_H_25_N_5_O_6_Na [M + Na]^+^ 442.1697, found 442.1695.

### Compound **20**

To a solution of **14** (1.51 g, 2.76 mmol) in dry acetonitrile (20 mL) were added *N* ^6^‐benzoyladenine (3.01 g, 12.6 mmol) and *N*,*O*‐bis(trimethylsilyl)acetamide (4.20 mL, 17.2 mmol) at 0 °C under N_2_ atmosphere. The suspension was refluxed until all the substrates were dissolved, and then the resulting solution was cooled to 0 °C. TMSOTf (1.49 mL, 8.25 mmol) was added, and the reaction mixture was refluxed for 14 hours. After the reaction was completed, saturated aqueous NaHCO_3_ was added, and the resulting mixture was extracted with AcOEt. The combined organic layer was washed with water and brine, dried over Na_2_SO_4_, and concentrated. To the crude product was added a small amount of AcOEt, and the mixture was stirred at the room temperature for 3 minutes. Then, the mixture was filtered through Celite and the Celite pad was washed with AcOEt. The filtrate was concentrated, and the crude product was purified by column chromatography (SiO_2_, hexane/AcOEt = 2:1→3:2) to afford **20** (1.31 g, 72%) as a white foam. ^1^H NMR (300 MHz, CDCl_3_) *δ* 1.79–1.88 (m, 2H), 2.01 (s, 3H), 2.33–2.49 (m, 4H), 3.62 (d, *J* = 10.2 Hz, 1H), 3.74 (d, *J* = 10.2 Hz, 1H), 4.50 (d, *J* = 11.1 Hz, 1H), 4.53–4.63 (m, 4H), 5.75–5.77 (m, 1H), 5.81 (dd, *J* = 5.4, 5.4 Hz, 1H), 6.51 (d, *J* = 5.7 Hz, 1H), 7.28–7.40 (m, 10H), 7.51–7.57 (m, 2H), 7.59–7.65 (m, 1H), 8.01–8.04 (m, 2H), 8.41 (s, 1H), 8.80 (s, 1H), 8.93 (brs, 1H); ^13^C NMR (125.8 MHz, CDCl_3_) *δ* 20.6, 23.4, 32.5, 33.3, 72.9, 73.5, 74.3, 76.1, 79.8, 85.5, 89.2, 123.3, 127.5, 127.6, 127.7, 127.8, 127.9, 128.0, 128.3, 128.6, 128.6, 132.6, 133.6, 137.1, 137.5, 141.7, 141.9, 149.5, 152.0, 152.6, 165.0, 170.0; HRMS (MALDI) Calcd. for C_38_H_37_N_5_O_6_Na [M + Na]^+^ 682.2636, found 682.2648.

### Compound **21**

To a solution of **20** (435 mg, 659 µmol) in methanol (10 mL) was added potassium carbonate (180 mg, 1.30 mmol) at 0 °C, and the reaction mixture was stirred at the same temperature for 50 minutes. After the completion of the reaction, saturated aqueous NaHCO_3_ was added, and the product was extracted with AcOEt. The organic layer was washed with brine and dried over Na_2_SO_4_. The organic layer was concentrated under reduced pressure and purified by column chromatography (SiO_2_, CHCl_3_/MeOH = 99:1) twice to afford **21** (407 mg, quant.) as a white form. ^1^H NMR (500 MHz, CDCl_3_) *δ* 1.81–1.91 (m, 2H), 2.36–2.39 (m, 2H), 2.41–2.54 (m, 2H), 3.60 (d, *J* = 10.5 Hz, 1H), 3.75 (d, *J* = 10.0 Hz, 1H), 4.04 (d, *J* = 8.0 Hz, 1H, OH), 4.37 (d, *J* = 5.0 Hz, 1H), 4.51 (d, *J* = 12.5 Hz, 1H), 4.54 (d, *J* = 12.0 Hz, 1H), 4.64 (d, *J* = 11.5 Hz, 1H), 4.69 (d, *J* = 11.5 Hz, 1H), 4.89 (ddd, *J* = 6.0, 6.0, 7.5 Hz, 1H), 5,79–5.80 (m, 1H), 6.23 (d, *J* = 5.5 Hz, 1H), 7.25–7.36 (m, 10H), 7.42–7.45 (m, 2H), 7.51–7.54 (m, 1H), 7.98–7.99 (m, 2H), 8.27 (s, 1H), 8.67 (s, 1H), 9.46 (brs, 1H); ^13^C NMR (125.8 MHz, CDCl_3_) *δ* 23.3, 32.6, 33.4, 73.5, 73.7, 74.4, 75.9, 81.2, 88.9, 122.9, 127.6, 127.8, 127.8, 127.9, 128.0, 128.1, 128.5, 128.6, 128.7, 132.6, 133.6, 137.2, 137.2, 141.6, 142.5, 149.3, 151.8, 152.5, 164.8; HRMS (MALDI) Calcd. for C_36_H_35_N_5_O_5_Na [M + Na]^+^ 640.2530, found 640.2527.

### Compound **22**

To a solution of **21** (390 mg, 631 µmol) in THF (15 mL) were added sodium hydrogen carbonate (310 mg, 3.69 mmol) and iodine (480 mg, 1.89 mmol) at room temperature, and the mixture was stirred at 60 °C for 22 hours under N_2_ atmosphere. After the completion of the reaction, saturated aqueous Na_2_S_2_O_3_ was added, and the product was extracted with AcOEt. The organic layer was washed with brine and dried over Na_2_SO_4_. The organic layer was concentrated under reduced pressure and purified by column chromatography (SiO_2_, hexane/AcOEt = 1:1) to afford **22** (261 mg, 56%) as a white form. ^1^H NMR (500 MHz, CDCl_3_) *δ* 1.84–1.92 (m, 1H), 1.99–2.06 (m, 1H), 2.09–2.20 (m, 2H), 2.23–2.29 (m, 1H), 2.52–2.60 (m, 1H), 3.88 (d, *J* = 11.5 Hz, 1H), 4.36 (d, *J* = 4.0 Hz, 1H), 4.51 (s, 3H), 4.54 (s, 1H), 4.63 (d, *J* = 12.0 Hz, 1H), 4.74 (d, *J* = 12.0 Hz, 1H), 4.80 (d, *J* = 10.5 Hz, 1H), 6.09 (s, 1H), 7.17–7.23 (m, 5H), 7.27–7.34 (m, 5H), 7.45–7.48 (m, 2H), 7.54–7.57 (m, 1H), 8.00–8.02 (m, 1H), 8.39 (s, 1H), 8.67 (s, 1H), 9.49 (brs, 1H); ^13^C NMR (125.8 MHz, CDCl_3_) *δ* 21.6, 29.9, 34.5, 36.2, 65.4, 72.7, 73.7, 80.2, 85.7, 89.5, 97.7, 123.7, 127.6, 127.6, 127.9, 128.0, 128.4, 128.6, 128.8, 132.8, 133.4, 136.9, 137.5, 140.8, 149.6, 150.7, 152.5, 164.9; HRMS (MALDI) Calcd. for C_36_H_34_N_5_O_5_NaI [M + Na]^+^ 766.1497, found 766.1505.

### Compound **23**

To a solution of **22** (204 mg, 274 µmol) in dry THF (2.7 mL) was added DBU (82.0 µL, 549 µmol) at room temperature, and the mixture was stirred at 60 °C for 24 hours under N_2_ atmosphere. After the completion of the reaction, water was added, and the product was extracted with AcOEt. The organic layer was washed with brine and dried over Na_2_SO_4_. The organic layer was concentrated under reduced pressure and purified by column chromatography (SiO_2_, hexane/AcOEt = 3:2→2:3) to afford **23** (142 mg, 84%) as a white form. ^1^H NMR (300 MHz, CDCl_3_) *δ* 2.00–2.10 (m, 1H), 2.16–2.26 (m, 1H), 2.33–2.42 (m, 1H), 2.47–2.55 (m, 1H), 3.71 (d, *J* = 11.1 Hz, 1H), 3.87 (d, *J* = 11.1 Hz, 1H), 4.39 (s, 1H), 4.49–4.69 (m, 4H), 4.78 (s, 1H), 5.89–5.91 (m, 1H), 6.08 (s, 1H), 6.13–6.15 (m, 1H), 7.22–7.38 (m, 10H), 7.51–7.55 (m, 2H), 7.59–7.62 (m, 1H), 8.02–8.04 (m, 2H), 8.35 (s, 1H), 8.76 (s, 1H), 9.02 (brs, 1H); ^13^C NMR (75.6 MHz, CDCl_3_) *δ* 31.7, 33.2, 64.5, 72.6, 73.9, 79.2, 86.2, 88.9, 98.7, 123.7, 127.6, 127.7, 128.0, 128.0, 128.1, 128.5, 128.7, 129.0, 131.1, 133.0, 133.7, 137.2, 137.7, 141.1, 149.5, 151.0, 152.8, 164.7; HRMS (MALDI) Calcd. for C_36_H_33_N_5_O_5_Na [M + Na]^+^ 638.2374, found 638.2373.

### Compound **24**

To a solution of **23** (83.0 mg, 135 µmol) in AcOEt (2 mL) was added palladium hydroxide 20% on carbon (32.9 mg, 40wt%), and the mixture was stirred at room temperature for 4 hours under H_2_ atmosphere. After completion of the reaction, the mixture was filtered Celite and the Celite pad was washed with AcOEt. The filtrate was concentrated, and the crude product was purified by column chromatography (SiO_2_, hexane/AcOEt/MeOH = 30:20:1) twice to afford **24** (68.0 mg, 82%) as a white form. ^1^H NMR (300 MHz, CDCl_3_) *δ* 1.44–2.18 (m, 8H), 3.85 (d, *J* = 11.4 Hz, 1H), 3.90 (d, *J* = 11.1 Hz, 1H), 4.33 (s, 1H), 4.49 (d, *J* = 11.7 Hz, 1H), 4.58 (d, *J* = 11.7 Hz, 1H), 4.61–4.71 (m, 2H), 4.71 (s, 1H), 6.05 (s, 1H), 7.22–7.37 (m, 10H), 7.49–7.54 (m, 2H), 7.58–7.63 (m, 1H), 8.02–8.05 (m, 2H), 8.32 (s, 1H), 8.74 (s, 1H), 9.19 (brs, 1H); ^13^C NMR (75.6 MHz, CDCl_3_) *δ* 23.6, 25.6, 35.8, 36.0, 65.0, 72.5, 73.9, 79.8, 86.1, 88.8, 95.2, 123.8, 127.6, 128.0, 128.5, 128.7, 128.9, 132.9, 133.6, 137.2, 137.6, 141.0, 149.5, 151.0, 152.7, 164.8; HRMS (MALDI) Calcd. for C_36_H_35_N_5_O_5_Na [M + Na]^+^ 640.2530, found 640.2528.

### Compound **25**

To a solution of **24** (445 mg, 771 µmol) in THF (7 mL) was added aqueous methylamine (40 wt%, 1.20 mL, 11.8 mmol) at 0 °C, and the reaction mixture was stirred at room temperature for 40 minutes. After the completion of the reaction, the resulting mixture was concentrated and extracted with AcOEt. The organic layer was washed with brine and dried over Na_2_SO_4_. The organic layer was concentrated under reduced pressure and purified by column chromatography (SiO_2_, hexane/AcOEt = 1:2→1:5) to afford **25** (333 mg, 90%) as a white form. ^1^H NMR (300 MHz, CDCl_3_) *δ* 1.42–2.16 (m, 8H), 3.84 (d, *J* = 11.1 Hz, 1H), 3.89 (d, *J* = 10.8 Hz, 1H), 4.32 (s, 1H), 4.47 (d, *J* = 11.7 Hz, 1H), 4.58 (d, *J* = 11.7 Hz, 1H), 4.60–4.69 (m, 2H), 4.69 (s, 1H), 5.99 (s, 1H), 6.11 (brs, 2H, NH_2_), 7.20–7.39 (m, 10H), 8.04 (s, 1H), 8.32 (s, 1H); ^13^C NMR (75.6 MHz, CDCl_3_) *δ* 23.7, 25.6, 35.8, 36.1, 65.2, 72.4, 73.9, 77.8, 79.8, 86.0, 88.6, 95.1, 120.2, 127.6, 127.7, 128.0, 128.0, 128.5, 128.7, 137.3, 137.8, 138.4, 149.0, 153.2, 155.7; HRMS (MALDI) Calcd. for C_29_H_32_N_5_O_4_Na [M + Na]^+^ 514.2449, found 514.2448.

### Compound **26**

To a solution of **25** (30.0 mg, 58.4 µmol) in EtOH/AcOH (2.2 mL, 10:1) were added palladium hydroxide 20% on carbon (12.1 mg, 40wt%) and ammonium formate (221 mg, 3.50 mmol), and the mixture was refluxed for 12 hours. After completion of the reaction, the mixture was filtered Celite and the Celite pad was washed with AcOEt and boiling MeOH. The filtrate was concentrated, and the crude product was purified by column chromatography (SiO_2_, CHCl_3_/MeOH = 9:1→5:1) three times to afford **26** (9.9 mg, 51%) as a white solid. ^1^H NMR (400 MHz, DMSO‐*d_6_* with a D_2_O drop) *δ* 1.41–1.69 (m, 5H), 1.73–1.88 (m, 3H), 2.00–2.08 (m, 1H), 3.77 (d, *J* = 12.8 Hz, 1H), 3.81 (d, *J* = 12.8 Hz, 1H), 4.28 (s, 1H), 4.33 (s, 1H), 5.82 (s, 1H), 8.14 (s, 1H), 8.22 (s, 1H); ^13^C NMR (100.6 MHz, DMSO‐*d_6_*) *δ* 23.0, 25.0, 34.8, 35.6, 56.3, 71.9, 79.6, 84.9, 89.4, 93.9, 119.1, 137.8, 148.5, 152.7, 156.0; HRMS (MALDI) Calcd. for C_15_H_20_N_5_O_4_ [M + H]^+^ 334.1510, found 334.1507.

## Supporting Information

The Supporting Information contains additional experimental procedures and complete characterization data for all new compounds, together with ^1^H, ^13^C, COSY, and NOESY NMR spectra, as well as Schemes S1 and S2, Tables S1–S4, and Figure S1.

## Conflicts of Interest

T. Yamaguchi and S. Obika are inventors of the patents of scpBNA and scpBNA2, and are collaborating with Luxna Biotech Co., Ltd. (Osaka, Japan).

## Supporting information



Supporting Information

## Data Availability

The data supporting the findings of this study are contained within the Supporting Information; additional data are available from the corresponding author upon reasonable request.
