# Supplementary material for: Development of Synthetic Routes to 2′‐O,4′‐C‐Spirocyclopentylene‐Bridged Nucleic Acids: Thymidine, Guanosine, and Adenosine
Source: Chemistry. 2025 Nov 7;31(70):e02995. doi: 10.1002/chem.202502995 (PMC12712746; doi:10.1002/chem.202502995)

**Supporting Information**

# Development of Synthetic Routes to 2′-*O*,4′-*C*-Spirocyclopentylene-Bridged Nucleic Acids: Thymidine, Guanosine, and Adenosine

Riku Kumagai,[a] Riko Yamada,[a] Takao Yamaguchi,[b,*] and Satoshi Obika[b,*]

[a] School of Pharmaceutical Sciences, The University of Osaka, 1-6 Yamadaoka, Suita, Osaka 565-0871, Japan
[b] Graduate School of Pharmaceutical Sciences, The University of Osaka, 1-6 Yamadaoka, Suita, Osaka 565-0871, Japan

* Corresponding authors: yamaguchi-ta@phs.osaka-u.ac.jp (T. Yamaguchi), obika@phs.osaka-u.ac.jp (S. Obika)

## Table of Contents

1. Supporting experimental data S2–S5
2. Supplementary experimental section S6–S10
3. $^{1}H$, $^{13}C$, COSY and NOESY spectra of synthesized compounds S11–S46

## 1. Supporting experimental data

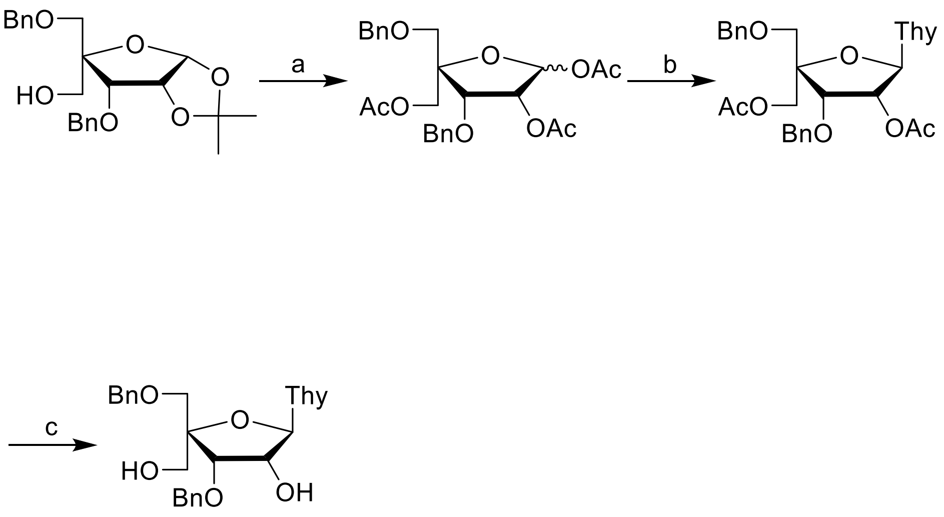


**1**

**Scheme S1.** Synthesis of the compound **1**. *Reagents and conditions*: a) AcOH, $Ac_2O$, $H_2SO_4$, rt, 2 h; b) thymine, BSA, TMSOTf, MeCN, reflux, 3.5 h, 88% (2 steps); c) $K_2CO_3$, MeOH, 0 °C, 12 h, quant.

**Table S1.** Investigation of *gem*-bisallylation[23]

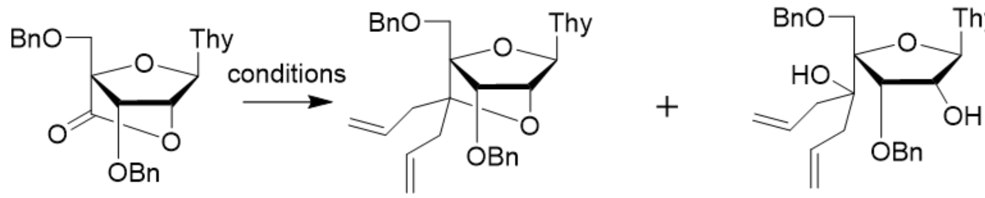


**2** **27** **3**

| entry | reagents | solvent | temp. | time | yield (%) | | |
|---|---|---|---|---|---|---|---|
| | | | | | **2** | **27** | **3** |
| 1 | Zn powder (6.2 eq.)<br>allyl bromide (4.0 eq.)<br>TMSCl (0.61 eq.) | THF | rt | 10 h | 42 | 0 | 26 |
| 2 | Zn powder (12.0 eq.)<br>allyl bromide (12.2 eq.)<br>TMSCl (0.97 eq.) | THF | 55 °C | 10 h | 0 | 0 | 42 |
| 3 | allylMgBr (2.2 eq.) | THF | −35 °C | 1 h | 0 | 0 | 82 |

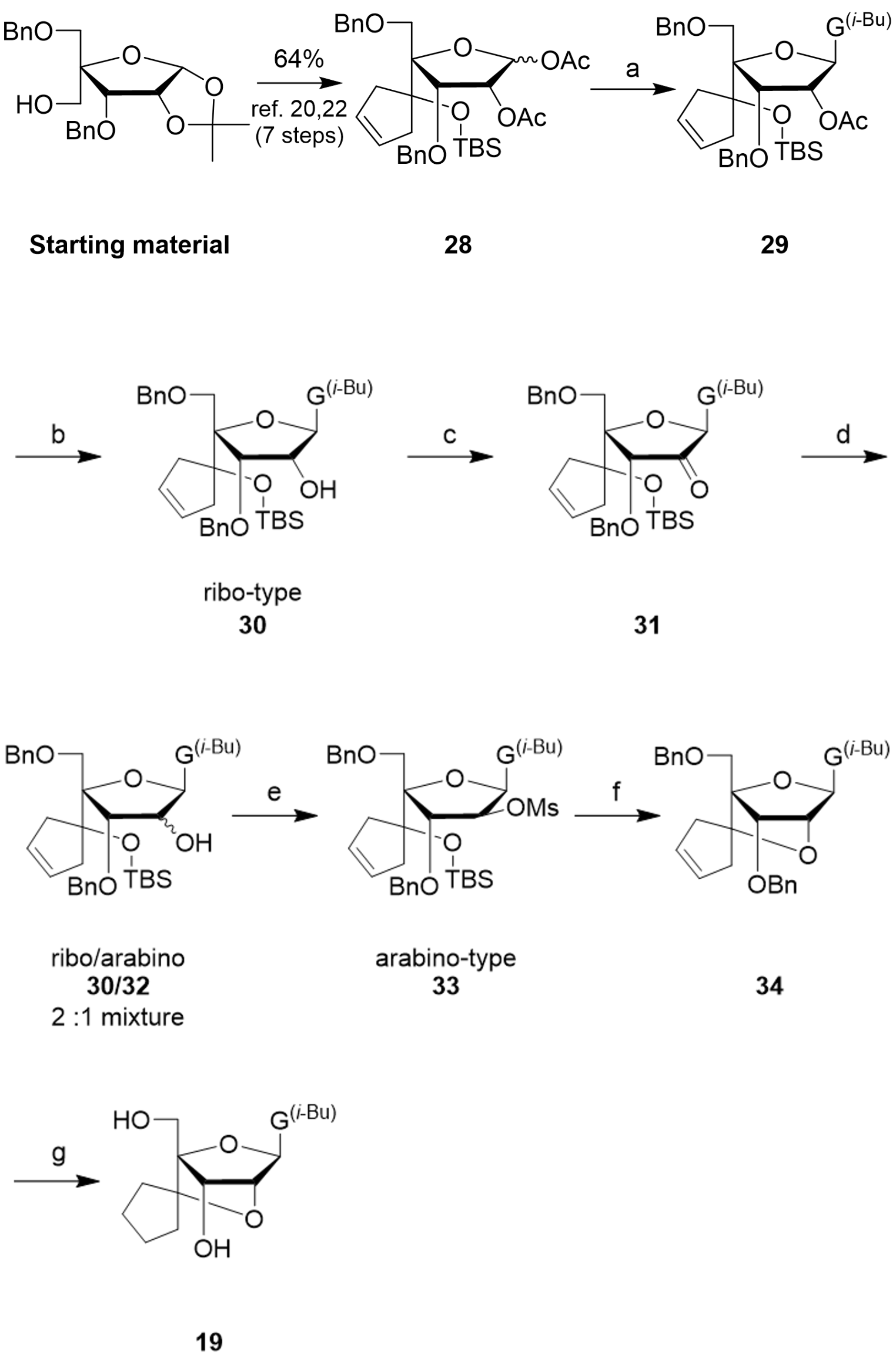


**Scheme S2.** Synthesis of the compound **19** *via* stereoinversion of the 2′-OH group. *Reagents and conditions*: (a) $N^2$-isobutyrylguanine, BSA, TMSOTf, MeCN, reflux, 13.5 h, 60%; (b) $K_2CO_3$, MeOH, 0 ºC, 1 h, 80%; (c) AZADOL, $PhI(OAc)_2$, $CH_2Cl_2$, rt, 18 h; (d) $NaBH_4$, $CH_2Cl_2$/MeOH, 0 ºC, 45 min, 88% (2 steps); (e) MsCl, pyridine, rt, 18 h, 29%; (f) TBAF, THF, reflux, 12 h, quant.; (g) $H_2$, $Pd(OH)_2$/C, AcOEt, rt, 20 h, 56%.

**Table S2.** Evaluation of acid-promoted cyclization toward 2′,4′-bridge formation

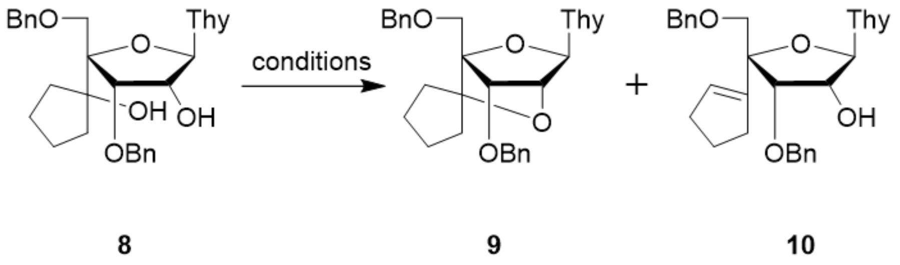


| entry | reagents | solvent | temp. | time | ratio[a] | | yield (%) | |
|---|---|---|---|---|---|---|---|---|
| | | | | | **9** | **10** | **9** | **10** |
| 1 | *p*-TsOH·$H_2O$ (0.5 eq.) | toluene | 80 ºC | 5 h | 10 | 90 | - | - |
| 2 | *p*-TsOH·$H_2O$ (0.5 eq.) | toluene | reflux | 2.5 h | 17 | 83 | 12 | 53 |
| 3 | *p*-TsOH·$H_2O$ (0.5 eq.) | MeCN | 50 ºC | 13 h | 3 | 97 | 4 | 70 |
| 4 | *p*-TsOH·$H_2O$ (0.5 eq.) | MeCN | reflux | 5 h | 7 | 93 | 6 | 46 |
| 5 | *p*-TsOH·$H_2O$ (0.5 eq.) | DMSO | reflux | 1 h | 6 | 94 | - | - |
| 6 | *p*-TsOH·$H_2O$ (0.5 eq.) | DMF | reflux | 5 h | N. R. | N. R. | N. R. | N. R. |

[a] Determined by $^1$H NMR.

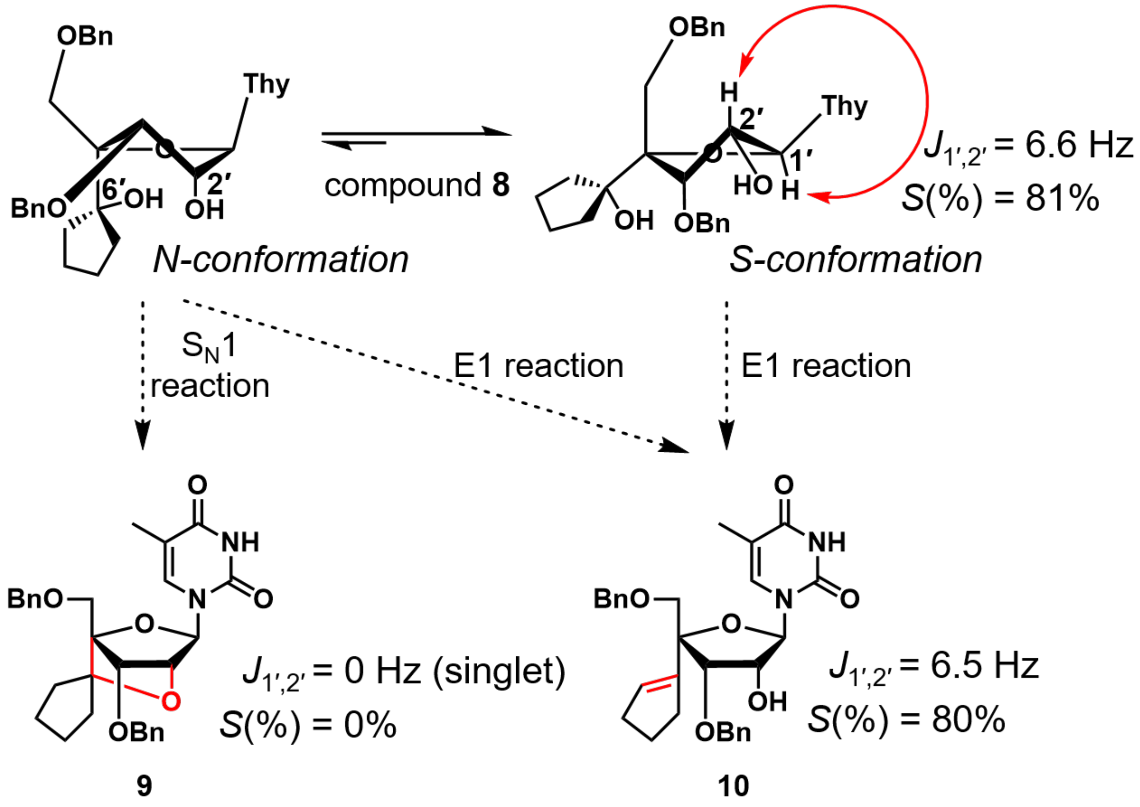


**Figure S1.** Conformationally driven competition between $S_N1$ and E1 passways. Notably, the conversion of the $S_N1$ product **9** to the E1 product **10** under acidic conditions (as examined in Table S2) proceeded only to a negligible extent. This indicates that compound **10** is not formed *via* the $S_N1$ intermediate **9**, but rather arises directly through the E1 elimination pathway.

**Table S3.** Investigation of dehalogenation

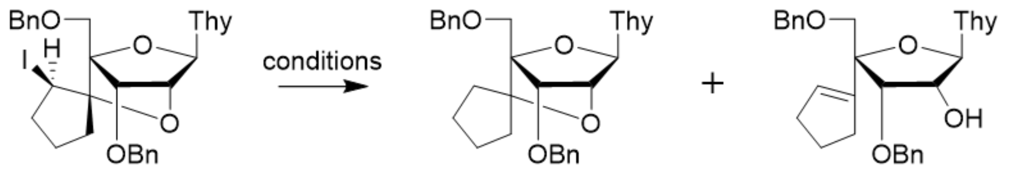

11 9 10

| entry | reagents | solvent | temp. | time | yield (%) | |
|---|---|---|---|---|---|---|
| | | | | | **9** | **10** |
| 1 | *t*-$Bu_3SnH$ (2.0 eq.)<br>AIBN (0.5 eq.) | toluene | 100 ºC | 40 min | 72 | 0 |
| 2 | $H_2$, Pd/C (40 wt%) | THF | reflux | 24 h | trace | 70 |
| 3 | $H_2$, TEA (2.0 eq.)<br>Pd/C (10 wt%) | THF | rt | 4 h | -[a] | 0 |
| 4 | Zn (5.0 eq.)<br>$NH_4Cl$ (25 eq.) | THF/$H_2O$ | rt | 15 min | 0 | quant. |
| 5 | Zn (5.0 eq.)<br>AcOH (10 eq.) | THF | rt | 10 min | 0 | quant. |

[a] The reaction was carried out with 70 mg of starting material **11** to obtain 33 mg of a mixture of **9** and **13** (**9**/**13** = 0.21:1) that was difficult to separate. The ratio is determined by $^1H$ NMR.

**Table S4.** Investigation of the catalytic hydrogenation

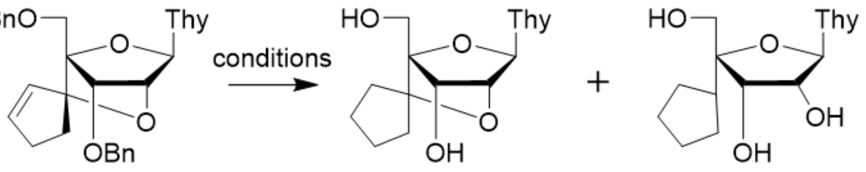

13 7 35

| entry | reagents | solvent | temp. | time | ratio[a] | | yield (%)[b] | |
|---|---|---|---|---|---|---|---|---|
| | | | | | **7** | **35** | **7** | **35** |
| 1 | $H_2$, $Pd(OH)_2/C$ (40 wt%) | MeOH | rt | 20 min | 1 | 8.2 | 8 | 73 |
| 2 | $H_2$, $Pd(OH)_2/C$ (40 wt%) | AcOEt | rt | 25 min | 1 | 2.7 | 19 | 47 |

[a] Determined by $^1H$ NMR. [b] **7** and **35** were isolated and purified by careful column chromatography using amino silica gel.

## 2. Supplementary experimental section

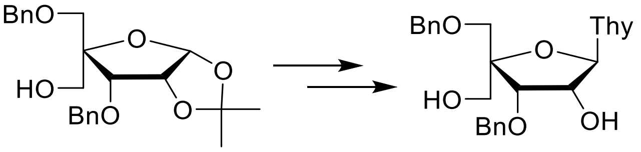


**1**

**Compound 1**

To a solution of 3,5-di-*O*-benzyl-4-*C*-hydroxymethyl-1,2-*O*-isopropylidene-α-D-ribofuranose (5.90 g, 14.7 mmol) in acetic acid (11.0 mL, 192 mmol) were added acetic anhydride (18.5 mL, 196 mmol) and sulfuric acid (83.0 µL, 1.55 mmol) at 0 ºC, and the mixture was stirred at room temperature for 2 h under $N_2$ atmosphere. After the completion of the reaction, saturated aqueous $NaHCO_3$ was added, and the product was extracted with AcOEt. The organic layer was washed with brine and dried over $Na_2SO_4$, and concentrated under reduced pressure. The crude was immediately used for the next reaction.

To a solution of the crude in dry acetonitrile (65 mL) were added thymine (4.47 g, 35.4 mmol) and *N*,*O*-bis(trimethylsilyl)acetamide (15.0 mL, 61.3 mmol) at 0 ºC under $N_2$ atmosphere. The solution was refluxed until all the substrates were dissolved, and then the resulting mixture was cooled to 0 ºC. TMSOTf (4.40 mL, 24.3 mmol) was added, and the mixture was refluxed for 3.5 h. After the reaction was completed, saturated aqueous $NaHCO_3$ was added, and the resulting mixture was extracted with AcOEt. The combined organic layer was washed with water and brine, dried over $Na_2SO_4$, and concentrated. The crude product was purified by column chromatography ($SiO_2$, hexane/AcOEt = 1:1) to afford 1-(3,5-di-*O*-benzyl-4-*C*-(hydroxymethyl)-β-D-ribofuranosyl)thymine (5.90 g, 88%) as a white foam.

To a solution of 1-(3,5-di-*O*-benzyl-4-*C*-(hydroxymethyl)-β-D-ribofuranosyl)thymine (5.90 g, 10.7 mmol) in methanol (75 mL) was added potassium carbonate (1.50 g, 10.9 mmol) at 0 ºC, and the reaction mixture was stirred at the same temperature for 12 h. After the completion of the reaction, saturated aqueous $NaHCO_3$ was added, and the product was extracted with AcOEt. The organic layer was washed with brine and dried over $Na_2SO_4$. The organic layer was concentrated under reduced pressure and purified by column chromatography ($SiO_2$, hexane/AcOEt = 1:3) to afford **1** (5.00 g, quant.) as a white form. The NMR spectral data of compound **1** was identical to those reported in the literature.[8]

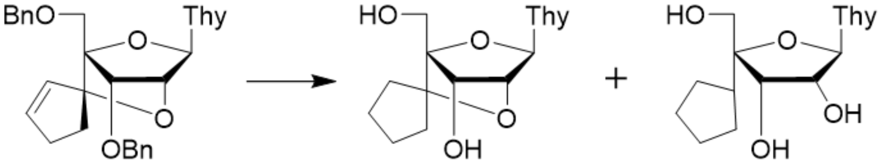


**13** **7** **35**

**Compounds 7 and 35**

To a solution of **13** (20.8 mg, 41.4 µmol) in AcOEt (1 mL) was added palladium hydroxide 20% on carbon (8.4 mg, 40wt%), and the mixture was stirred at room temperature for 25 min under $H_2$ atmosphere. After completion of the reaction, the mixture was filtered through Celite and the Celite pad was washed with AcOEt and MeOH. The filtrate was concentrated, and the crude product was purified by column chromatography ($SiO_2$–$NH_2$, $CHCl_3$/MeOH = 10:1→5:1) to afford **35** (6.4 mg, 47%) as a white solid and **7** (2.5 mg, 19%) as a white solid.

**35**: $^1$H NMR (300 MHz, $CD_3OD$) δ 1.36–1.59 (m, 6H), 1.76–1.89 (m, 2H), 1.89 (d, $J$ = 0.9 Hz, 3H), 2.33–2.44 (m, 1H), 3.65

(s, 2H), 4.17 (d, $J$ = 5.7 Hz, 1H), 4.44 (dd, $J$ = 5.7, 7.8 Hz, 1H), 5.95 ($J$ = 8.1 Hz, 1H), 7.86 ($J$ = 1.5 Hz, 1H); $^{13}$C NMR (75.6 MHz, $CD_3OD$) δ 12.4, 26.6, 26.7, 28.5, 28.9, 44.5, 65.8, 74.4, 75.3, 89.1, 92.0, 111.9, 138.7, 153.1, 166.4; HRMS (MALDI) Calcd. for $C_{15}H_{22}N_2O_6Na$ [M + Na]$^+$ 349.1370, found 349.1370.

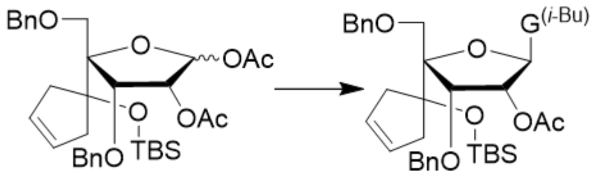


**28** **29**

**Compound 29**

To a solution of **28**[20,22] (11.3 g, 18.5 mmol) in dry acetonitrile (200 mL) were added $N^2$-isobutyrylguanine (12.2 g, 55.1 mmol) and *N*,*O*-bis(trimethylsilyl)acetamide (22.6 mL, 92.2 mmol) at 0 ºC under $N_2$ atmosphere. The suspension was refluxed until all the substrates were dissolved, and then the resulting solution was cooled to 0 ºC. TMSOTf (5.2 mL, 28.8 mmol) was added, and the reaction mixture was refluxed for 13.5 h. After the reaction was completed, saturated aqueous $NaHCO_3$ was added, and the resulting mixture was extracted with AcOEt. The combined organic layer was washed with water and brine, dried over $Na_2SO_4$, and concentrated. To the crude product was added a small amount of AcOEt, and the mixture was stirred at room temperature for 3 min. Then, the mixture was filtered. The filtrate was concentrated, and the crude product was purified by column chromatography ($SiO_2$, hexane/AcOEt/MeOH = 10:3:1) to afford **29** (8.64 g, 60%) as a white foam.

$^1$H NMR (300 MHz, $CDCl_3$) δ –0.10 (s, 3H), –0.02 (s, 3H), 0.76 (s, 9H), 1.23 (d, $J$ = 6.6 Hz, 3H), 1.24 (d, $J$ = 6.9 Hz, 3H), 1.84 (s, 3H), 2.15–2.24 (m, 1H), 2.49–2.58 (m, 1H), 2.61–2.89 (m, 3H), 3.67 (d, $J$ = 9.6 Hz, 1H), 4.03 (d, $J$ = 9.6 Hz, 1H), 4.41 (d, $J$ = 11.7 Hz, 1H), 4.52 (d, $J$ = 4.8 Hz, 1 H), 4.55 (d, $J$ = 11.1 Hz, 1H), 4.76 (d, $J$ = 11.7 Hz, 1H), 4.83 (d, $J$ = 11.4 Hz, 1H), 5.57–5.66 (m, 2H), 5.97 (dd, $J$ = 5.1, 8.7 Hz, 1H), 6.14 (d, $J$ = 8.7 Hz, 1H), 7.29–7.47 (m, 10H), 8.02 (s, 1H), 8.25 (brs, 1H), 11.9 (brs, 1H); $^{13}$C NMR (75.6 MHz, $CDCl_3$) δ −3.3, −3.2, 18.5, 19.1, 19.1, 20.6, 26.3, 36.7, 44.6, 44.8, 58.5, 73.4, 74.0, 75.2, 81.7, 84.2, 87.6, 92.3, 121.1, 127.3, 127.7, 127.9, 128.2, 128.3, 128.4, 128.9, 129.0, 137.2, 137.6, 138.3, 147.4, 148.8, 155.6, 170.2, 178.1; HRMS (MALDI) calcd. for $C_{41}H_{53}N_5O_8NaSi$ [M + Na]$^+$ 794.3556, found 794.3544.

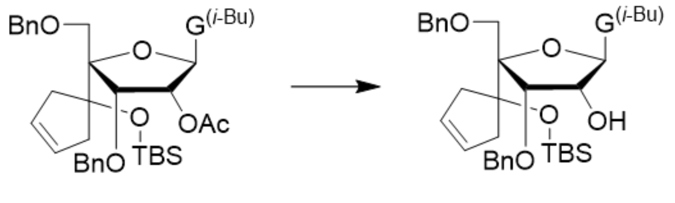


ribo-type

**29** **30**

**Compound 30**

To a solution of **29** (3.80 g, 4.92 mmol) in methanol (50 mL) was added potassium carbonate (1.36 g, 9.84 mmol) at 0 ºC, and the reaction mixture was stirred at the same temperature for 1 h. After the completion of the reaction, saturated aqueous $NaHCO_3$ was added, and the product was extracted with AcOEt. The organic layer was washed with brine and dried over $Na_2SO_4$. The organic layer was concentrated under reduced pressure and purified by column chromatography ($SiO_2$, $CHCl_3$/MeOH = 20:1) to afford **30** (2.88 g, 80%) as a white form.

$^1$H NMR (300 MHz, $CDCl_3$) δ –0.07 (s, 3H), 0.00 (s, 3H), 0.78 (s, 9H), 1.16 (d, $J$ = 6.6 Hz, 3H), 1.18 (d, $J$ = 6.6 Hz, 3H),

2.21–2.26 (m, 1H), 2.53–2.62 (m, 2H), 2.79–2.90 (m, 2H), 3.64 (d, $J$ = 9.9 Hz, 1H), 3.96–3.98 (m, 2H), 4.25 (d, $J$ = 4.8 Hz, 1H), 4.43 (d, $J$ = 12.0 Hz, 1H), 4.61 (d, $J$ = 12.0 Hz, 1H), 4.68–4.79 (m, 2H), 4.94–5.01 (m, 1H), 5.61–5.64 (m, 2H), 5.76 (d, $J$ = 7.5 Hz, 1H), 7.21–7.35 (m, 10H), 7.98 (s, 1H), 9.33 (brs, 1H), 12.02 (brs, 1H); $^{13}$C NMR (125.8 MHz, $CDCl_3$) δ −3.2, −3.1, 18.4, 18.5, 18.9, 19.0, 26.3, 36.4, 44.8, 44.9, 58.3, 73.7, 73.9, 75.6, 83.1, 87.5, 89.0, 92.3, 120.8, 127.8, 127.9, 128.0, 128.1, 128.4, 128.7, 128.8, 137.3, 137.5, 137.9, 147.5, 148.5, 155.8, 178.7; HRMS (MALDI) calcd. for $C_{39}H_{51}N_5O_7NaSi$ [M + Na]$^+$ 752.3450, found 752.3448.

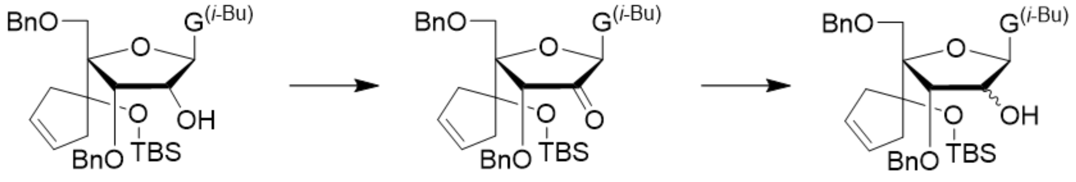


ribo-type
**30**

**31**

ribo/arabino
**30/32**
2 :1 mixture

**Compounds 31 and 32**

To a solution of **30** (1.62 g, 2.22 mmol) in dichloromethane (45 mL) were added iodobenzene diacetate (1.15 g, 3.57 mmol) and 2-hydroxy-2-azaadamantane (17.8 mg, 116 μmol, 0.05 eq.) at 0 ºC, and the reaction mixture was stirred at room temperature for 18 h under $N_2$ atmosphere. After the completion of the reaction, saturated aqueous $NaHCO_3$ and saturated aqueous $Na_2S_2O_3$ were added, and the product was extracted with AcOEt. The organic layer was washed with brine and dried over $Na_2SO_4$, and concentrated under reduced pressure. The crude ketone **31** (1.60 g, a yellow form) was used immediately for the next reaction without further purification.

To a solution of the crude ketone **31** (1.60 g) in methanol/dichloromethane (36 mL, 1:2) was added sodium borohydride (163 mg, 4.31 mmol) at 0 ºC, and the reaction mixture was stirred at the same temperature for 45 min under $N_2$ atmosphere. After the completion of the reaction, saturated aqueous $NH_4Cl$ was added, and the product was extracted with AcOEt. The organic layer was washed with brine and dried over $Na_2SO_4$. The organic layer was concentrated under reduced pressure and purified by column chromatography ($SiO_2$, $CHCl_3$/MeOH = 19:1) to afford a mixture of stereoisomers **30** and **32** (1.42 g, 88%, **30/32** = 2:1) as a white form, which were difficult to separate.

**31**: HRMS (MALDI) calcd. for $C_{39}H_{49}N_5O_7NaSi$ [M + Na]$^+$ 750.3293, found 750.3302.

**30** and **32**: $^1$H NMR (400 MHz, $CDCl_3$) δ –0.13 (s, 1H), –0.06 (s, 2H), –0.03 (s, 1H), 0.06 (s, 2H), 0.76 (s, 3H), 0.78 (s, 6H), 1.13–1.19 (m, 6H), 2.22–2.26 (m, 2/3H), 2.28–2.33 (m, 1/3H), 2.51–2.68 (m, 7/3H), 2.80–2.89 (m, 4/3H), 2.95–2.99 (m, 1/3H), 3.66 (d, $J$ = 9.6 Hz, 2/3H), 3.91–4.10 (m, 5/3H), 4.25–4.29 (m, 1/3H), 4.26 (d, $J$ = 4.4 Hz, 2/3H), 4.45 (d, $J$ = 11.6 Hz, 2/3H), 4.47–4.52 (m, 2/3H), 4.50 (d, $J$ = 11.6 Hz, 1/3H), 4.63–4.70 (m, 2H), 4.80 (s, 2/3H), 4.94–5.00 (m, 2/3H), 5.61–5.65 (m, 2H), 5.76 (d, $J$ = 7.6 Hz, 2/3H), 6.11 (s, 1/3H), 7.22–7.37 (m, 10H), 7.88 (s, 1/3H), 7.99 (s, 2/3H), 8.73 (brs, 1/3H), 8.98 (brs, 2/3H), 11.89 (brs, 1/3H), 11.96 (brs, 2/3H); $^{13}$C NMR (125.8 MHz, $CDCl_3$) δ –3.4, –3.3, –3.2, –3.1, 18.4, 18.5, 18.9, 19.0, 26.1, 26.3, 36.4, 36.5, 44.8, 44.8, 45.7, 45.8, 73.2, 73.7, 74.0, 74.2, 75.4, 75.7, 83.2, 87.0, 87.3, 87.5, 88.3, 88.6, 90.7, 92.1, 120.6, 120.7, 127.5, 127.7, 127.9, 128.1, 128.2, 128.3, 128.5, 128.8, 128.9, 137.2, 137.4, 137.8, 147.1, 147.4, 148.4, 155.6, 155.6, 178.4, 178.5; HRMS (MALDI) calcd. for $C_{39}H_{51}N_5O_7NaSi$ [M + Na]$^+$ 752.3450, found 752.3464.

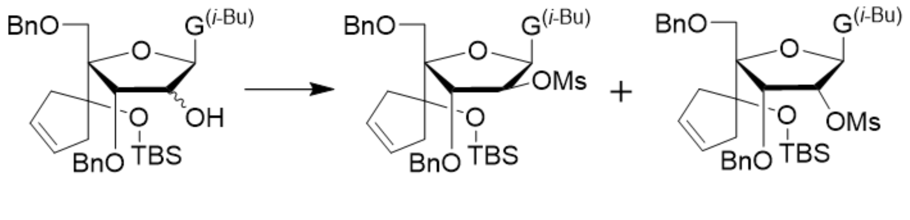


**Compounds 33 and ribo-mesylate**

To a solution of stereoisomers **30** and **32** (720 mg, 986 μmol, **30/32** = 2:1) in dry pyridine (10 mL) was added methanesulfonyl chloride (120 μL, 1.55 mmol) at 0 ºC, and the reaction mixture was stirred at room temperature for 18 h under $N_2$ atmosphere. After the completion of the reaction, saturated aqueous $NaHCO_3$ was added, and the product was extracted with $CHCl_3$. The organic layer was washed with brine and dried over $Na_2SO_4$, and concentrated under reduced pressure. The residue was purified by column chromatography ($SiO_2$, hexane/AcOEt = 10:7) to afford ribo-mesylate (595 mg, 53%) as a white form and **33** (328 mg, 29%) as a white form.

ribo-mesylate: $^1$H NMR (400 MHz, $CDCl_3$) δ –0.11 (s, 3H), –0.03 (s, 3H), 0.78 (s, 9H), 1.15 (d, *J* = 6.8 Hz, 3H), 1.16 (d, *J* = 3.2 Hz, 3H), 2.17–2.22 (m, 1H), 2.50 (s, 3H), 2.55–2.64 (m, 2H), 2.73–2.78 (m, 1H), 3.73 (d, *J* = 10.0 Hz, 1H), 4.03 (d, *J* = 10.0 Hz, 1H), 4.41 (d, *J* = 4.8 Hz, 1H), 4.54 (d, *J* = 11.6 Hz, 1H), 4.71–4.74 (m, 2H), 4.79 (d, *J* = 10.4 Hz, 1H), 5.55–5.60 (m, 2H), 5.95 (dd, *J* = 4.8, 8.4 Hz, 1 H), 6.11 (d, *J* = 8.4 Hz, 1H), 7.25–7.41 (m, 10H), 7.96 (s, 1H), 9.23 (brs, 1H), 12.12 (brs, 1H); $^{13}$C NMR (100.6 MHz, $CDCl_3$) δ –3.5, –3.4, 18.3, 18.8, 18.9, 26.1, 36.1, 37.2, 44.4, 44.5, 73.0, 73.9, 74.9, 80.8, 81.3, 83.8, 87.1, 92.0, 120.7, 127.4, 127.6, 127.7, 128.0, 128.2, 128.2, 128.8, 129.0, 136.8, 137.0, 137.6, 147.8, 148.8, 155.6, 178.9; HRMS (MALDI) calcd. for $C_{40}H_{53}N_5O_9NaSiS$ [M + Na]$^+$ 830.3225, found 830.3236.

**33**: $^1$H NMR (400 MHz, $CDCl_3$) δ 0.09 (s, 6H), 0.92 (s, 9H), 1.22 (d, *J* = 6.8 Hz, 3H), 1.22 (d, *J* = 6.8 Hz, 3H), 2.39–2.44 (m, 2H), 2.49 (s, 3H), 2.57–2.64 (m, 1H), 2.87–2.92 (m, 2H), 3.43–3.50 (m, 2H), 4.38 (d, *J* = 11.6 Hz, 1H), 4.52–4.57 (m, 2H), 4.73 (d, *J* = 11.6 Hz, 3H), 4.90 (d, *J* = 5.2 Hz, 1H), 5.56–5.60 (m, 2H), 5.90 (s, 1H), 6.49 (d, *J* = 6.4 Hz, 1H), 7.25–7.38 (m, 10H), 7.95 (s, 1H), 8.72 (brs, 1H), 12.08 (brs, 1H); $^{13}$C NMR (100.5 MHz, $CDCl_3$) δ –2.8, –2.8, 18.5, 18.9, 19.2, 26.4, 36.5, 38.1, 44.5, 45.8, 69.6, 73.6, 73.8, 80.4, 81.7, 81.8, 87.0, 89.3, 120.4, 128.1, 128.2, 128.2, 128.4, 128.5, 128.7, 129.2, 129.2, 137.3, 139.0, 147.6, 148.5, 155.8, 178.6; HRMS (MALDI) calcd. for $C_{40}H_{53}N_5O_9NaSiS$ [M + Na]$^+$ 830.3225, found 830.3235.

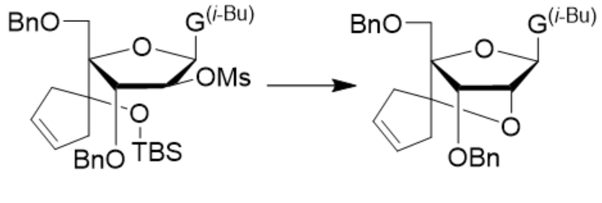


**Compound 34**

To a solution of **33** (221 mg, 274 μmol) in THF (4 mL) was added 1 M tetrabutylammonium fluoride in tetrahydrofuran (1.09 mL, 1.09 mmol) at 0 ºC, and the reaction mixture was refluxed for 12 h under $N_2$ atmosphere. After the completion of the reaction, the mixture was concentrated. The residue was purified by column chromatography ($SiO_2$, hexane/AcOEt = 1:1) to afford **34** (164 mg, quant.) as a white form.

$^1$H NMR (400 MHz, $CDCl_3$) δ 1.24 (d, *J* = 6.8 Hz, 3H), 1.24 (d, *J* = 6.8 Hz, 3H), 2.40–2.45 (m, 1H), 2.54–2.67 (m, 2H), 2.73–

2.79 (m, 1H), 2.94–2.98 (m, 1H), 3.72 (d, $J$ = 11.2 Hz, 1H), 3.78 (d, $J$ = 11.2 Hz, 1H), 4.23 (s, 1H), 4.41–4.46 (m, 1H), 4.43 (s, 1H), 4.49–4.60 (m, 3H), 5.59 (s, 1H), 5.60–5.64 (m, 2H), 7.17–7.31 (m, 10H), 7.87 (s, 1H), 9.81 (brs, 1H), 12.17 (brs, 1H); $^{13}$C NMR (100.6 MHz, $CDCl_3$) δ 19.0, 36.3, 42.0, 42.6, 65.0, 72.4, 73.7, 78.0, 79.2, 85.7, 88.2, 93.3, 121.5, 127.5, 127.6, 128.0, 128.4, 128.5, 128.6, 136.3, 137.1, 137.5, 147.4, 148.0, 155.8, 179.3; HRMS (MALDI) calcd. for $C_{33}H_{35}N_5O_6Na$ [M + Na]$^+$ 620.2480, found 620.2482.

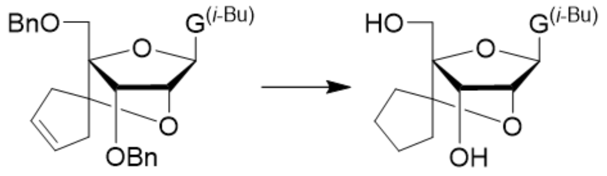


**34** **19**

**Compound 19**

To a solution of **34** (40.4 mg, 67.6 μmol) in AcOEt (1 mL) was added palladium hydroxide 20% on carbon (20.2 mg, 50wt%), and the mixture was stirred at room temperature for 20 h under $H_2$ atmosphere. After completion of the reaction, the mixture was filtered through Celite and the Celite pad was washed with AcOEt and MeOH. The filtrate was concentrated, and the crude product was purified by column chromatography ($SiO_2$, $CHCl_3$/MeOH = 15:1→9:1) to afford **19** (15.8 mg, 56%) as a white solid.

## 3. $^{1}$H, $^{13}$C, COSY and NOESY spectra of synthesized compounds

Compound **2** ($^{1}$H-NMR, $CDCl_3$, 500 MHz), ($^{13}$C NMR, $CDCl_3$, 125.7 MHz)

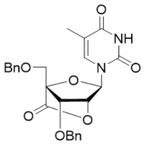


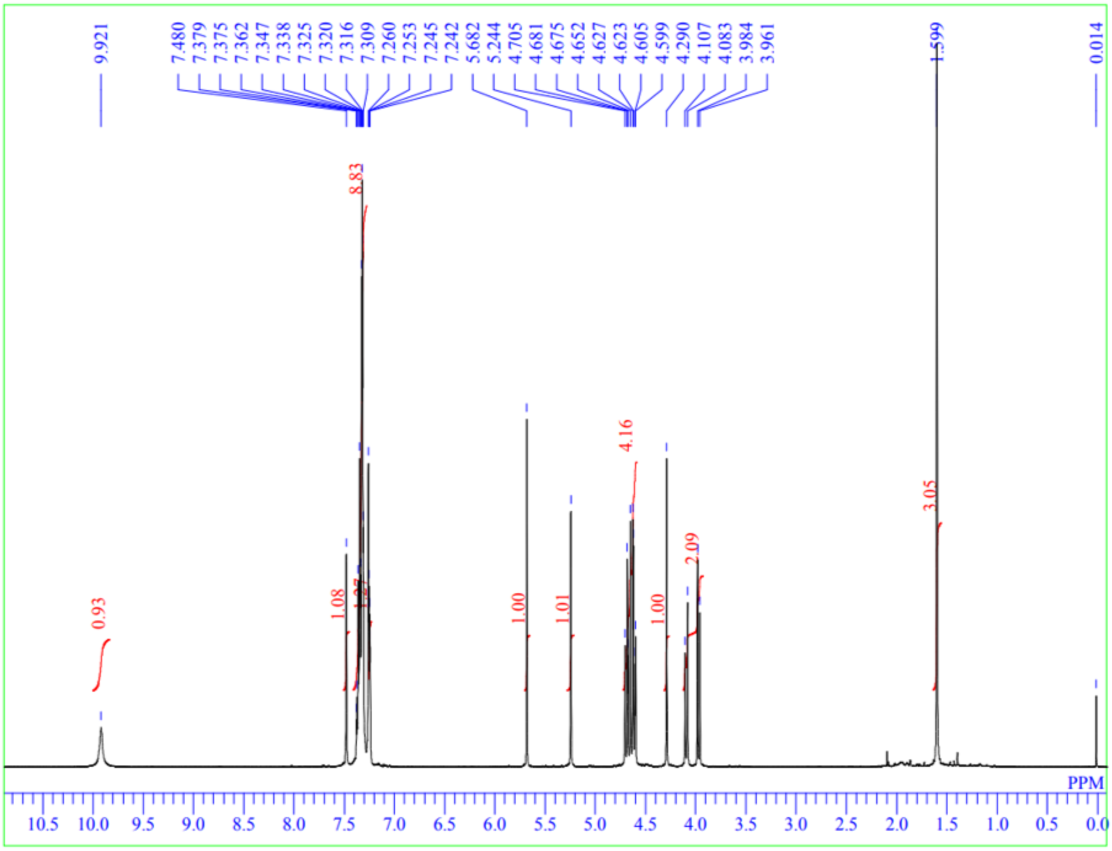


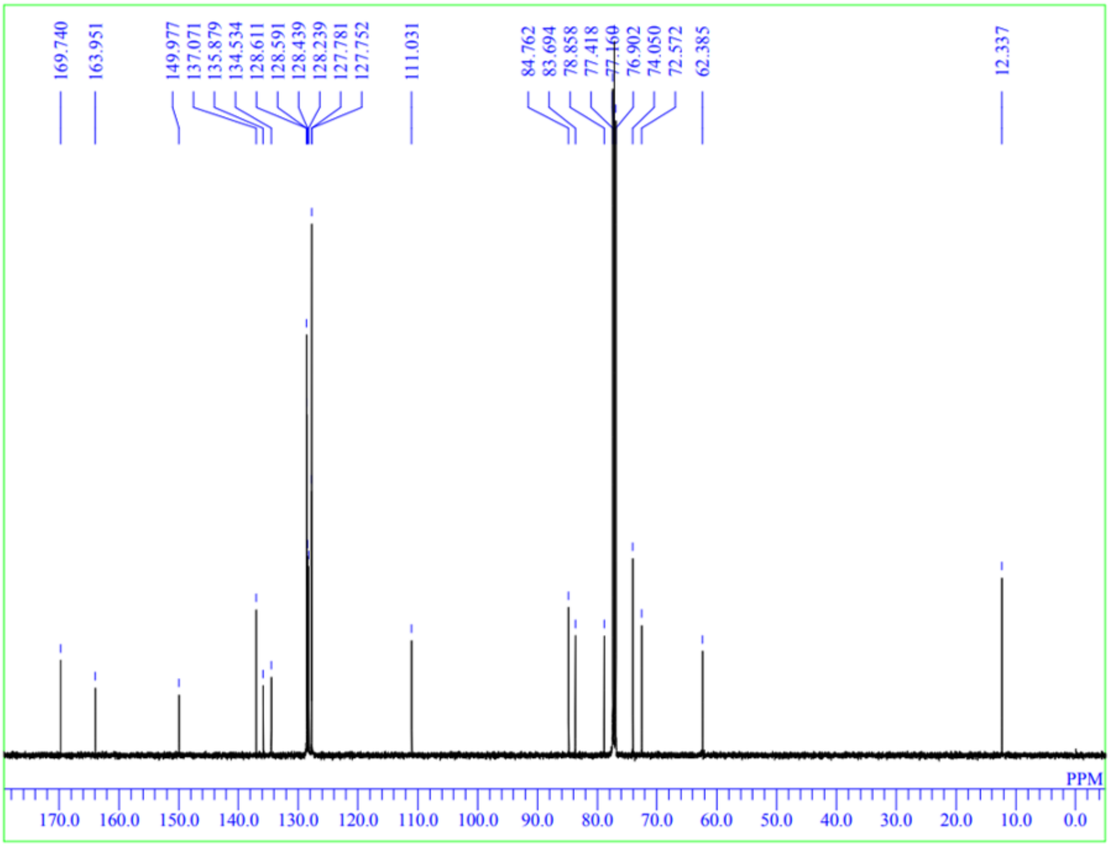

Compound **3** ($^{1}$H-NMR, $CDCl_3$, 500 MHz), ($^{13}$C NMR, $CDCl_3$, 75.6 MHz)

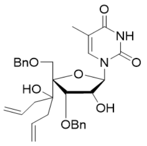


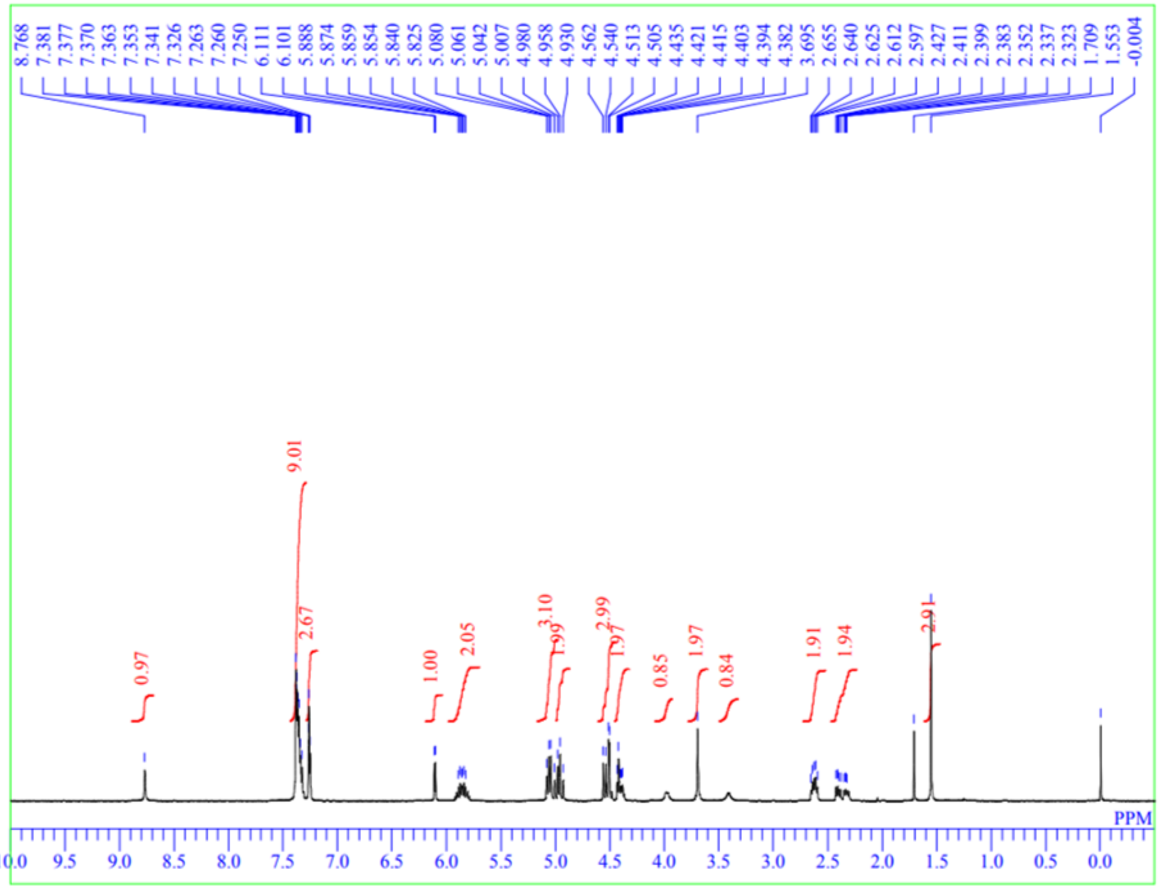


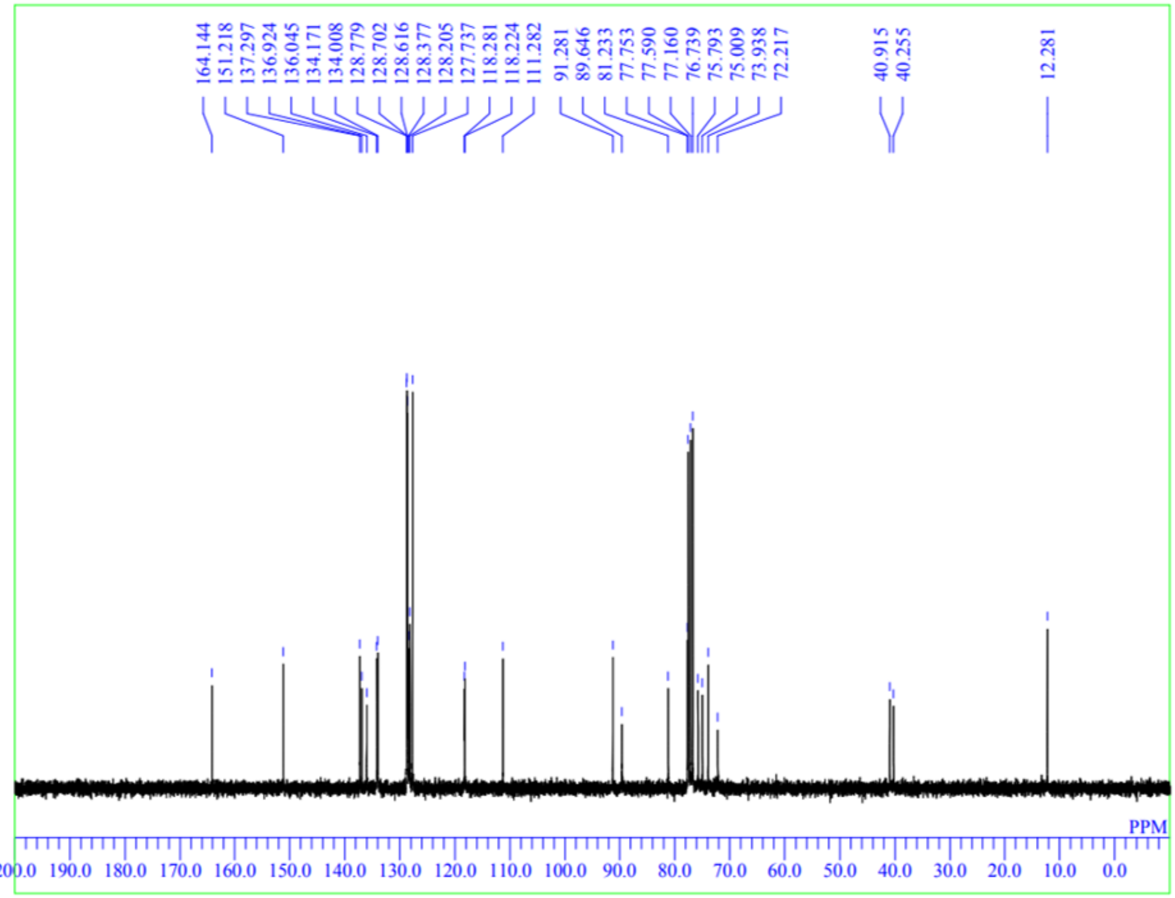

Compound **4** ($^{1}$H-NMR, $CDCl_3$, 300 MHz), ($^{13}$C NMR, $CDCl_3$, 75.6 MHz)

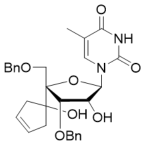


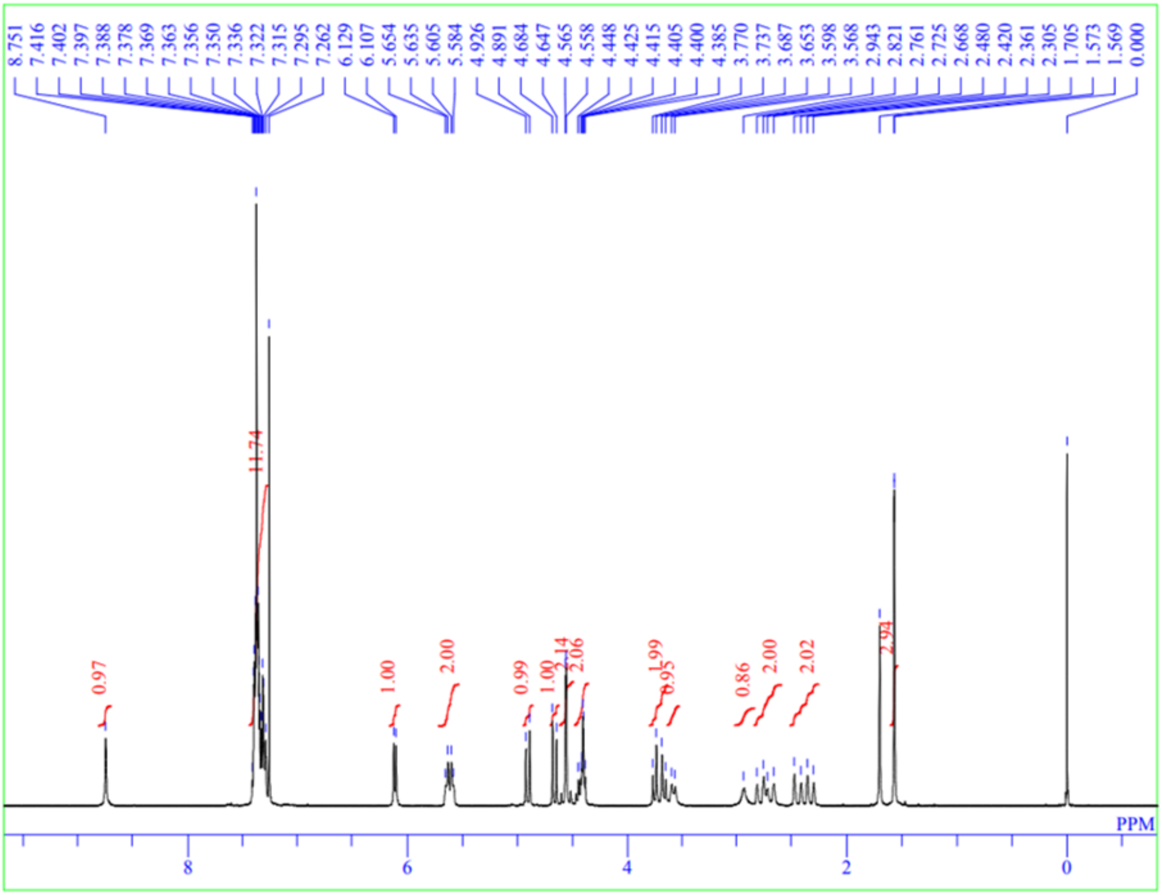


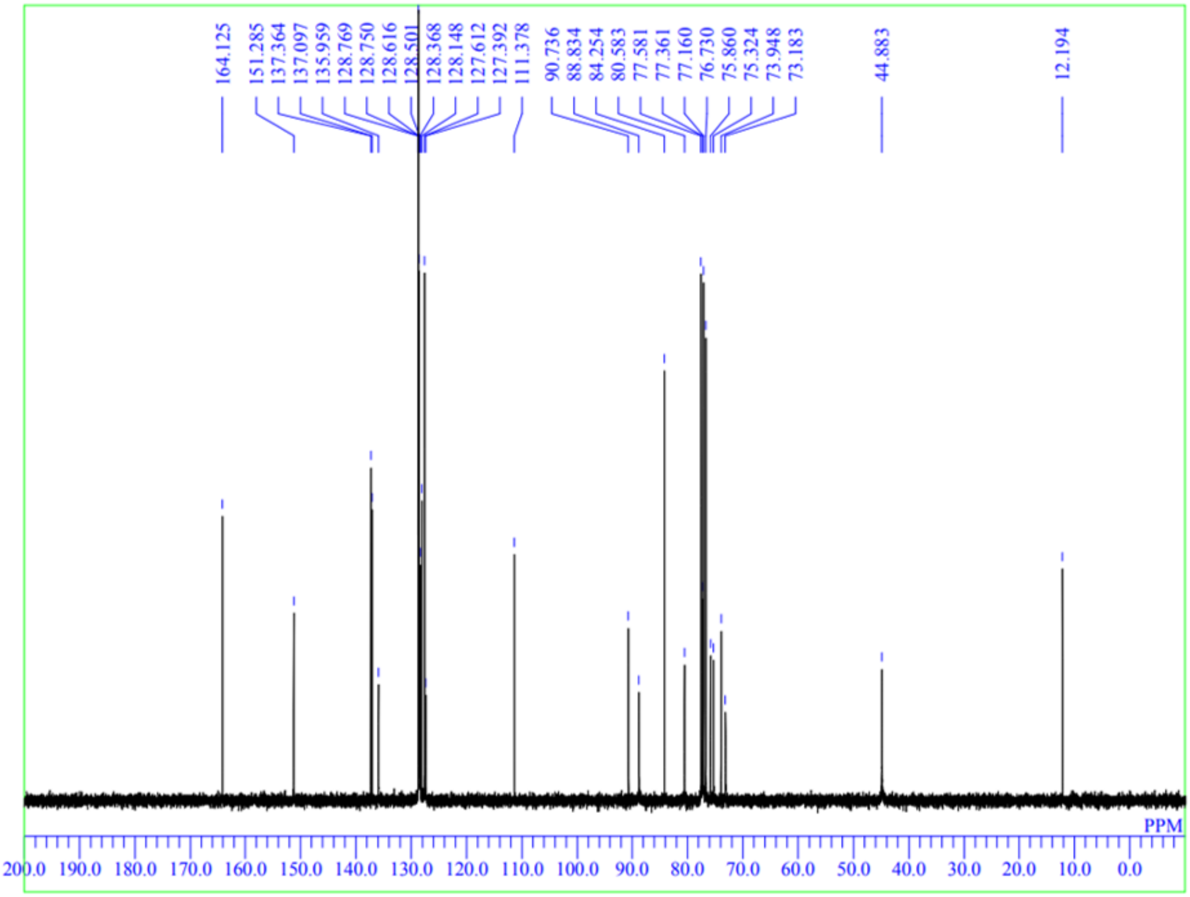

Compound **5** ($^{1}$H-NMR, $CDCl_3$, 400 MHz), ($^{13}$C NMR, $CDCl_3$, 100.6 MHz)

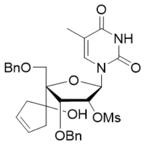


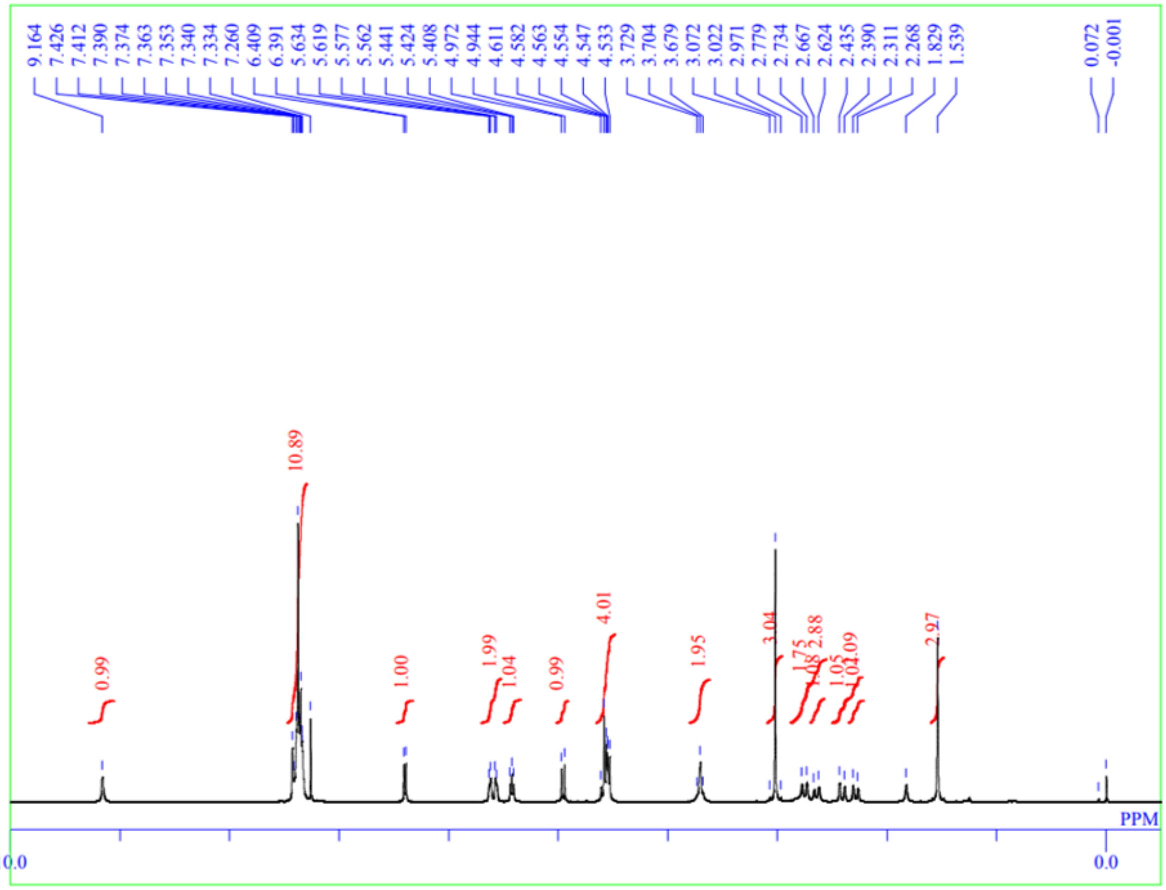


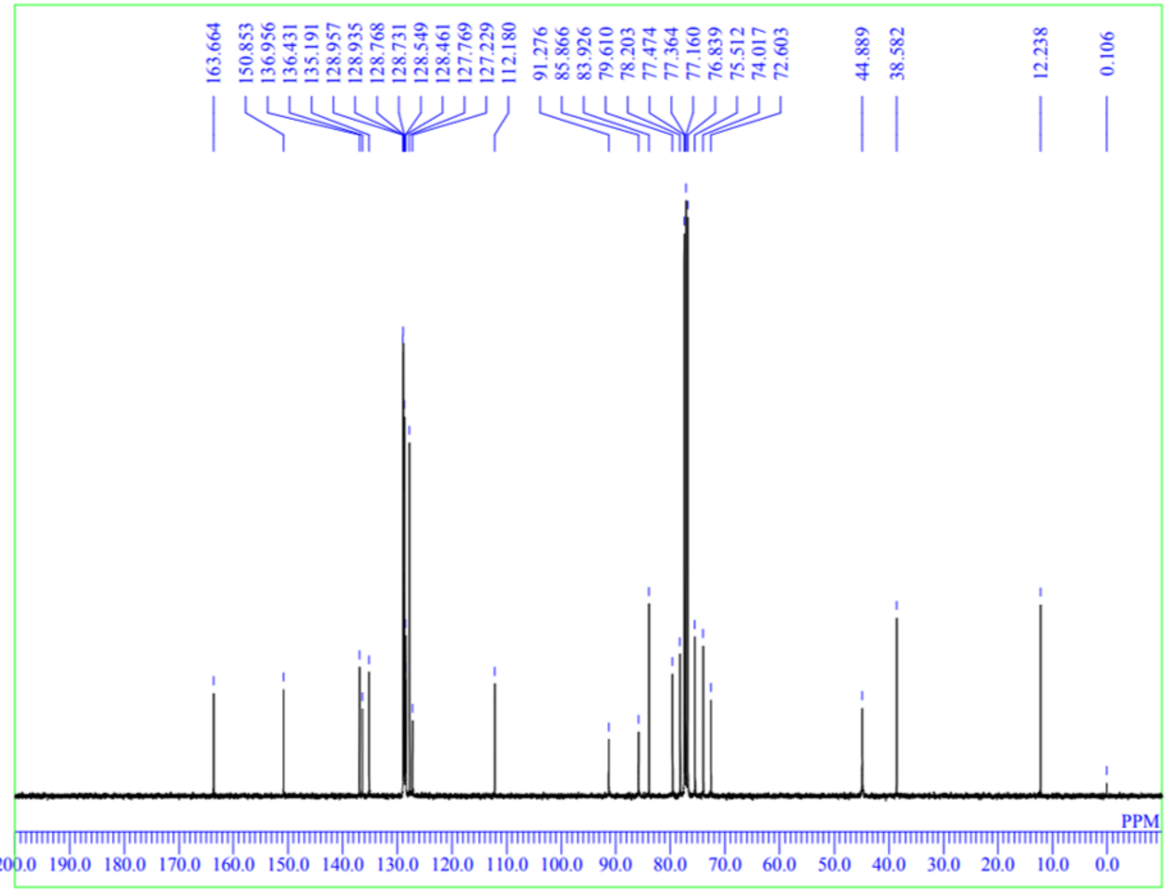

Compound **8** ($^{1}$H-NMR, $CDCl_3$, 300 MHz), ($^{13}$C NMR, $CDCl_3$, 75.6 MHz)

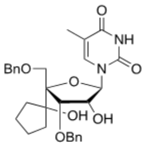


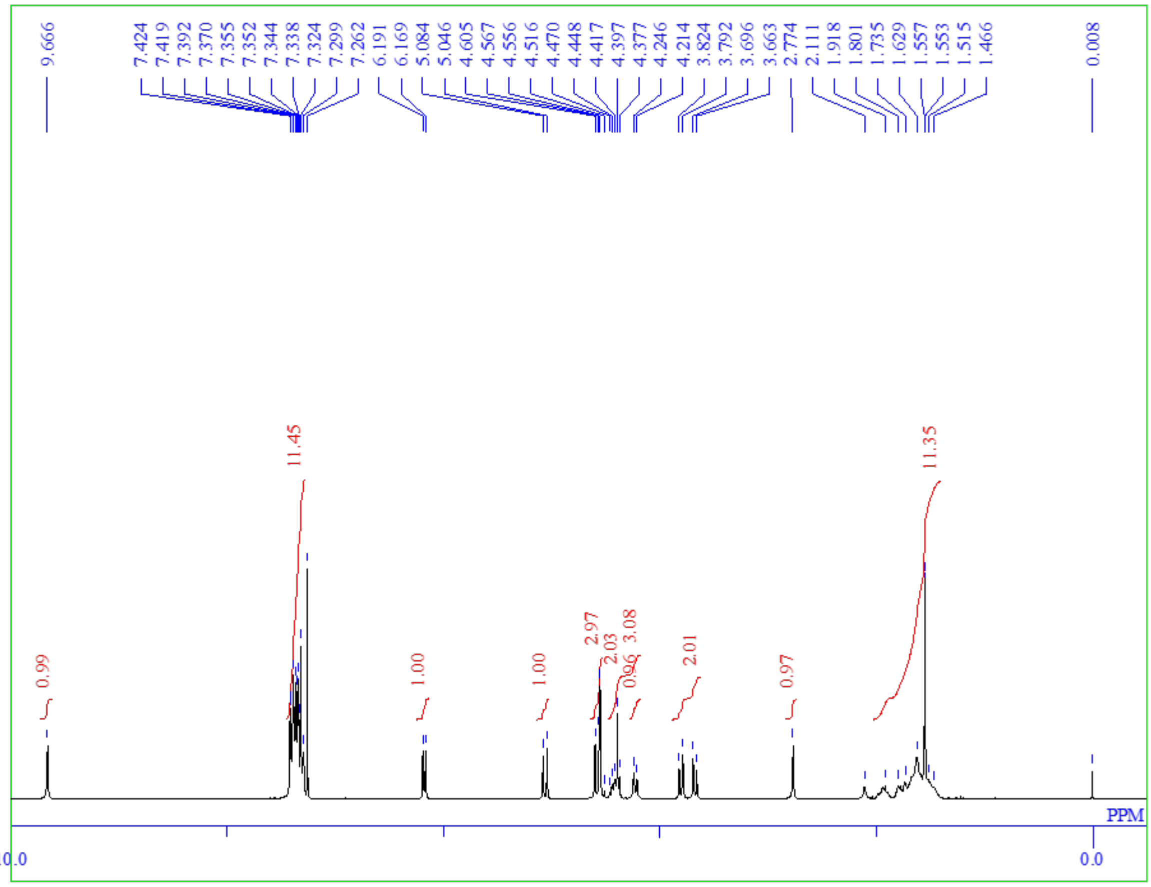


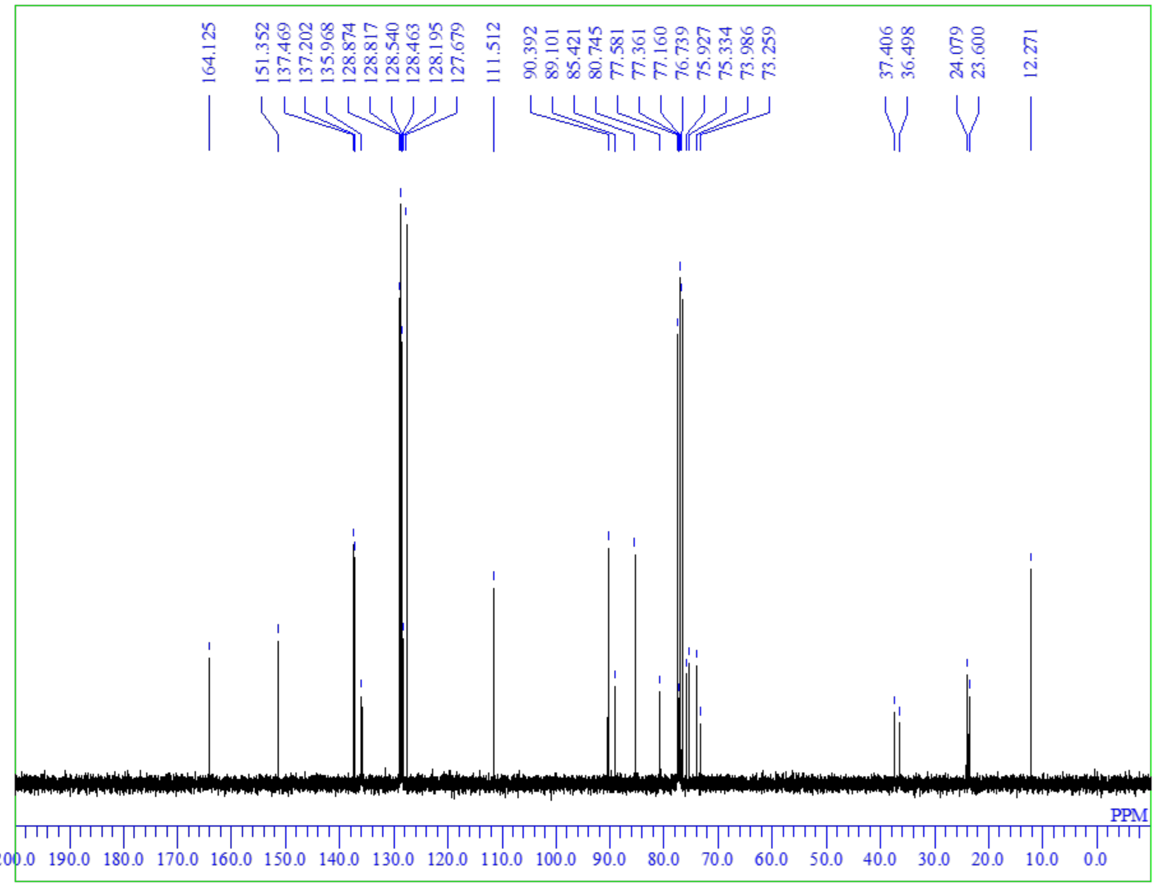

Compound **9** ($^1$H-NMR, $CDCl_3$, 500 MHz), ($^{13}$C NMR, $CDCl_3$, 125.8 MHz)

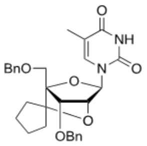


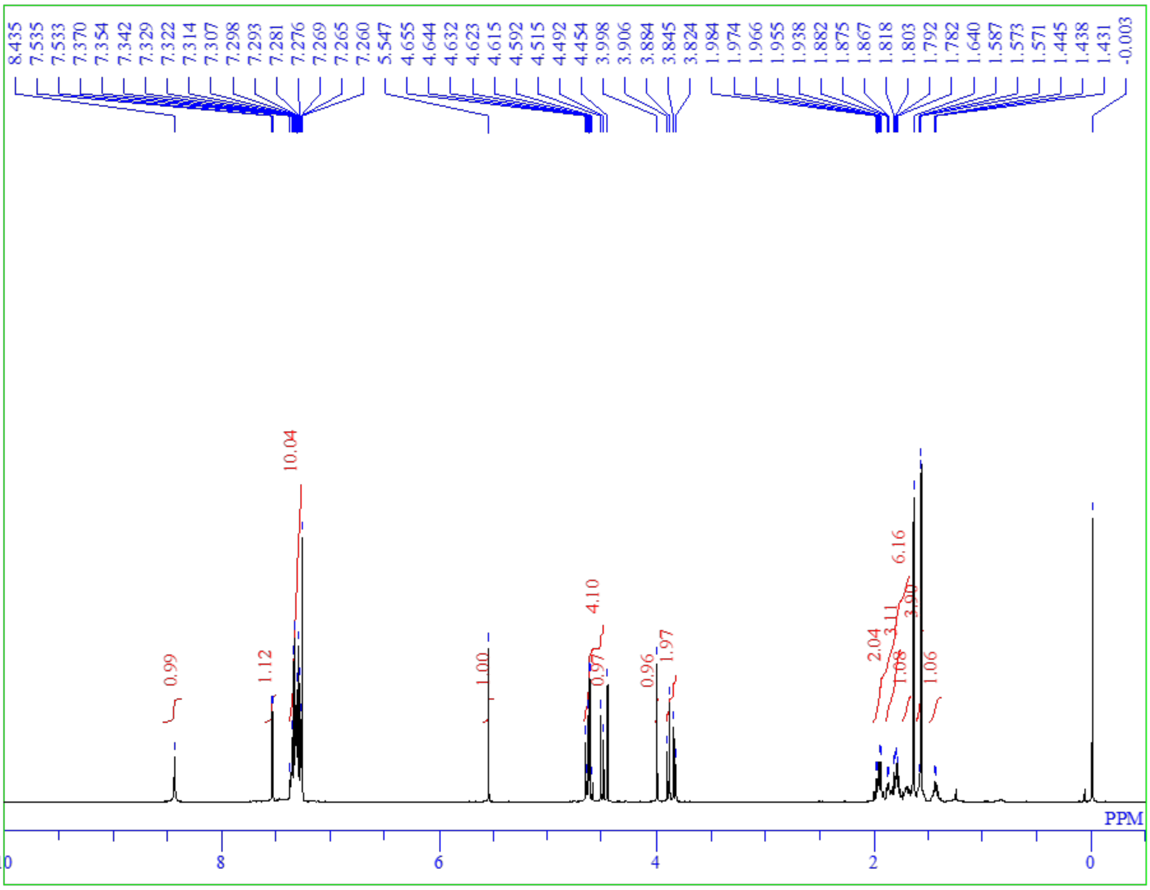


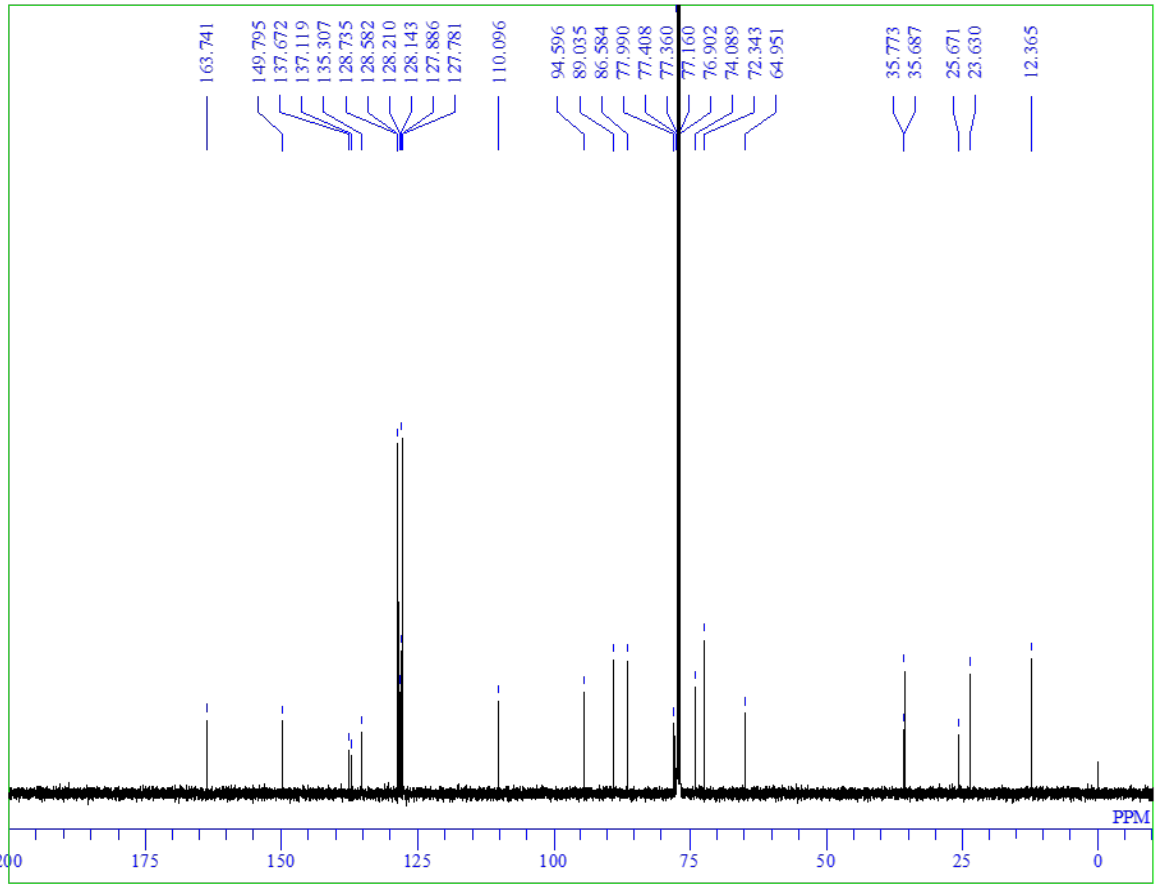

Compound **10** ($^{1}$H-NMR, $CDCl_3$, 500 MHz), ($^{13}$C NMR, $CDCl_3$, 75.6 MHz)

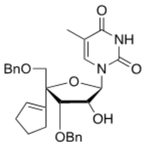


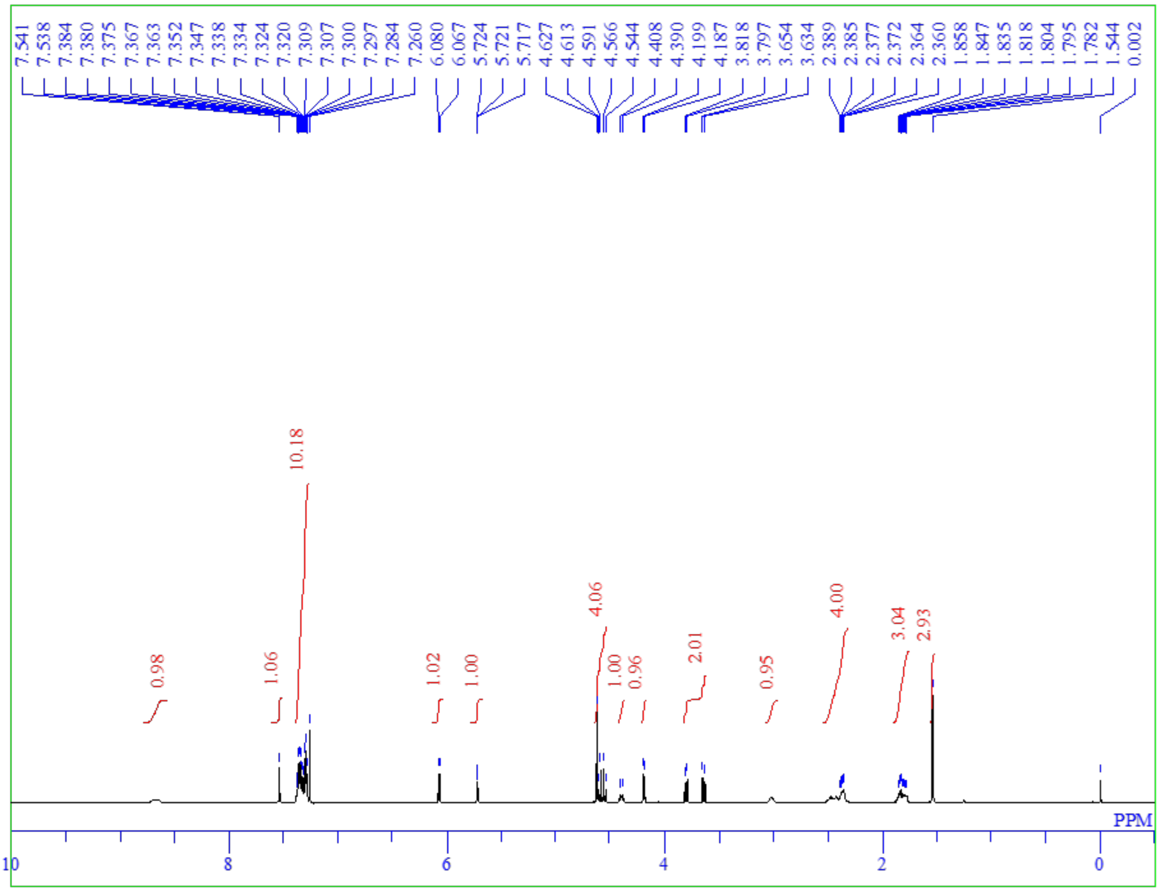


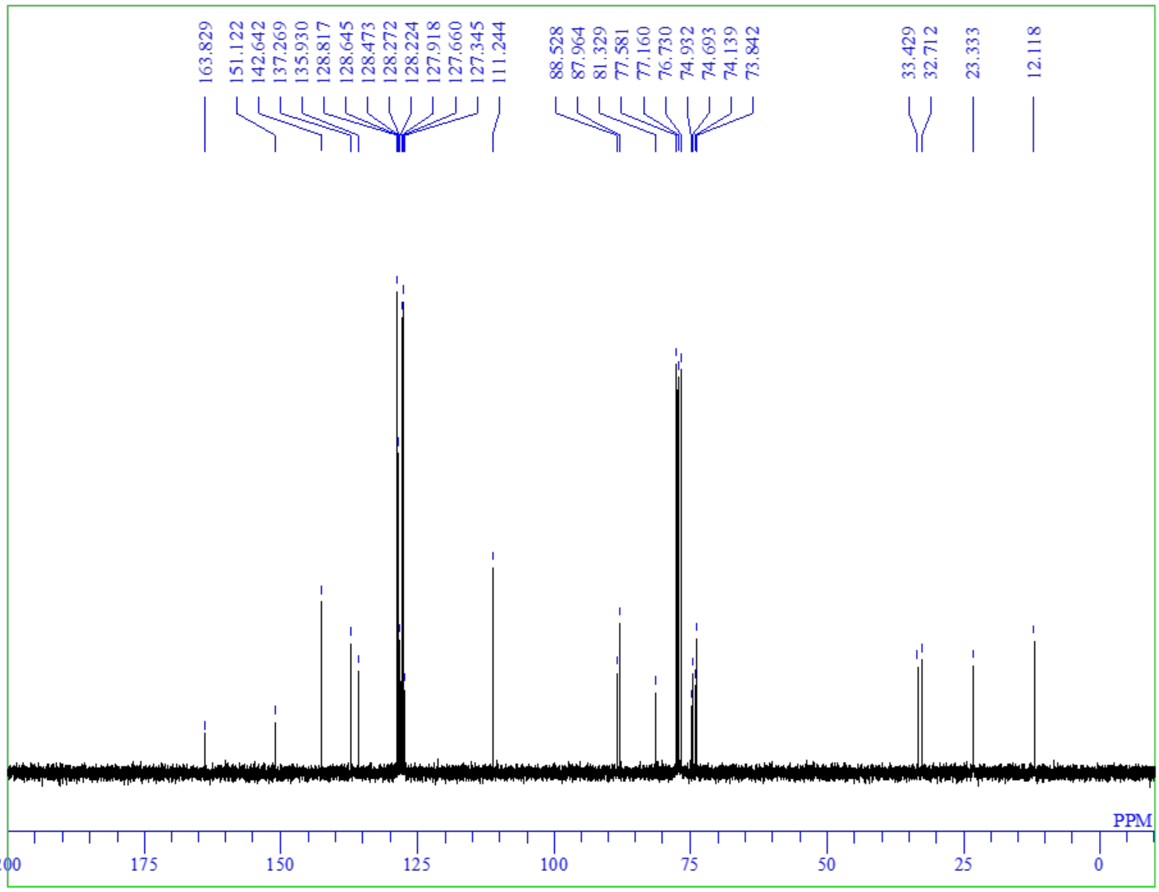

Compound **11** ($^1$H-NMR, $CDCl_3$, 500 MHz), ($^{13}$C NMR, $CDCl_3$, 125.8 MHz)

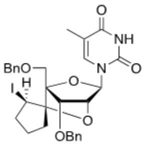


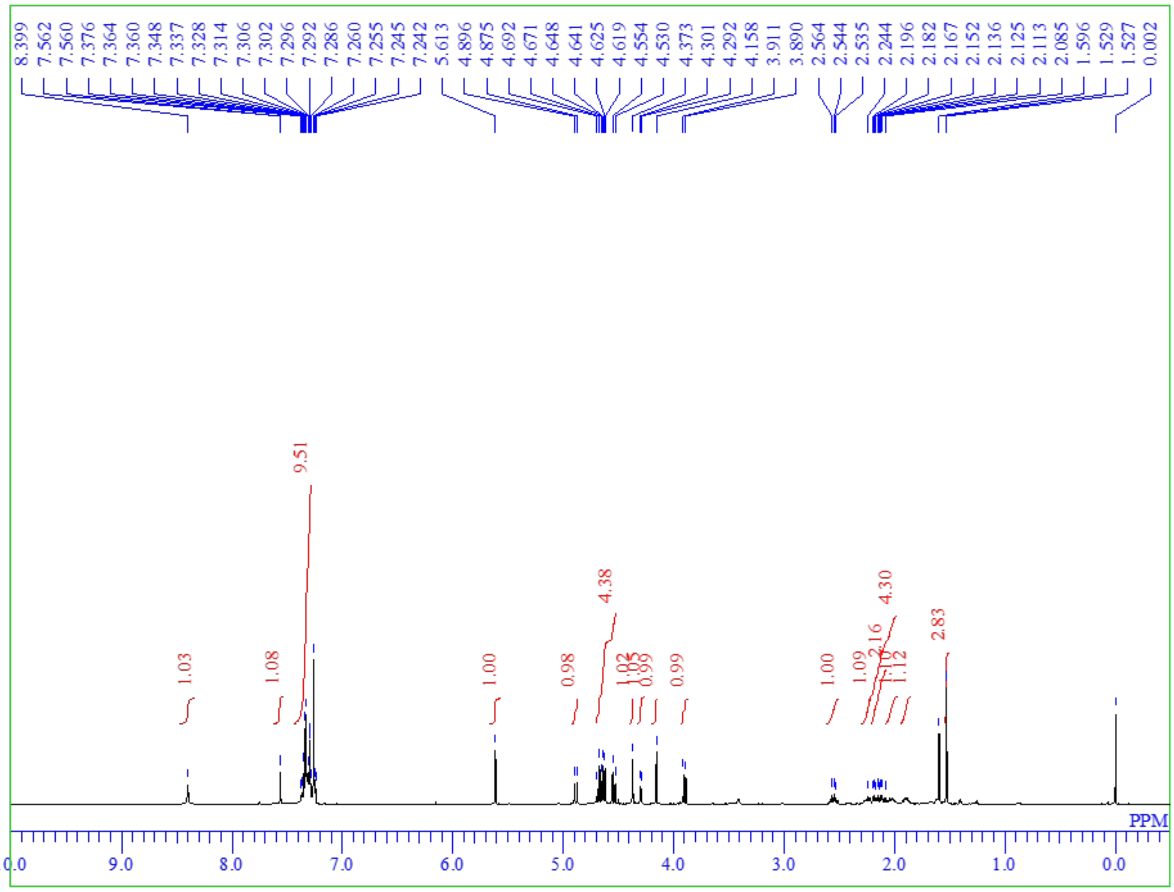

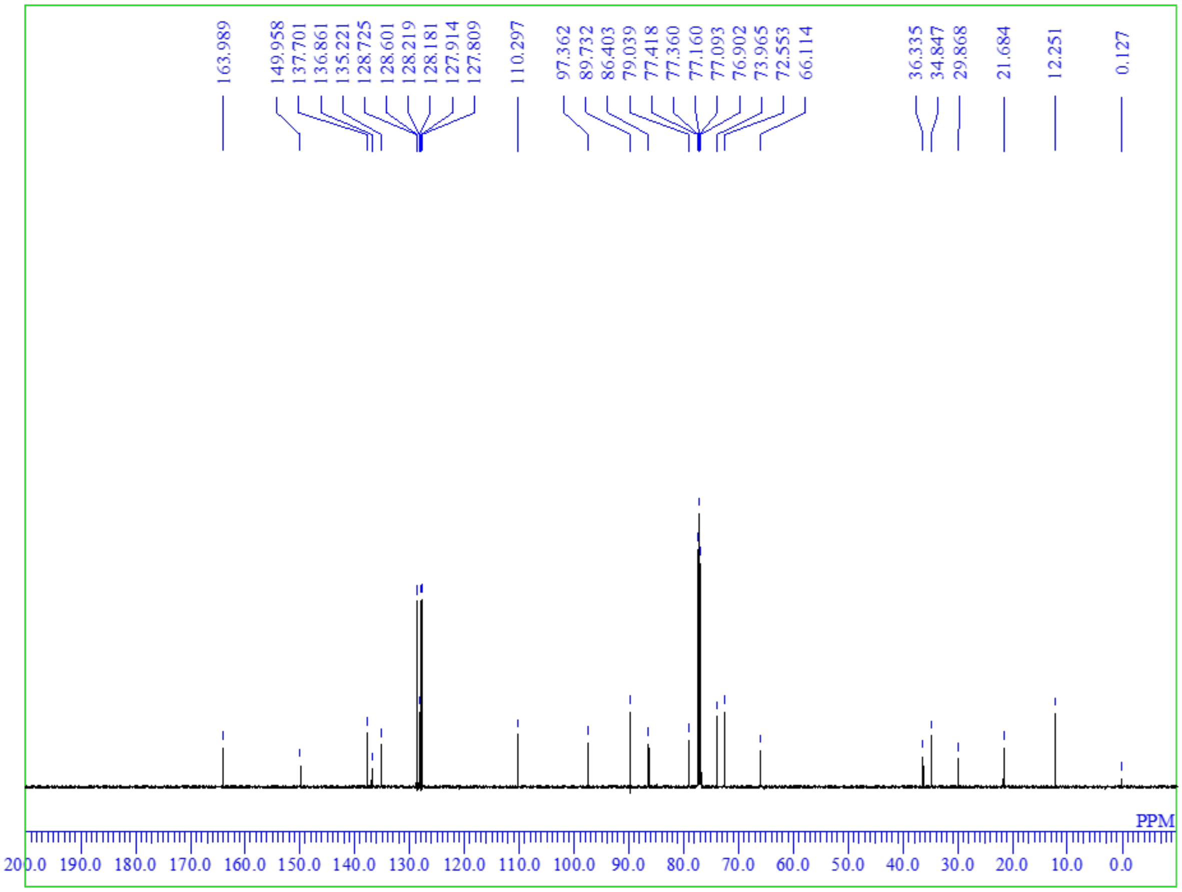

COSY spectrum

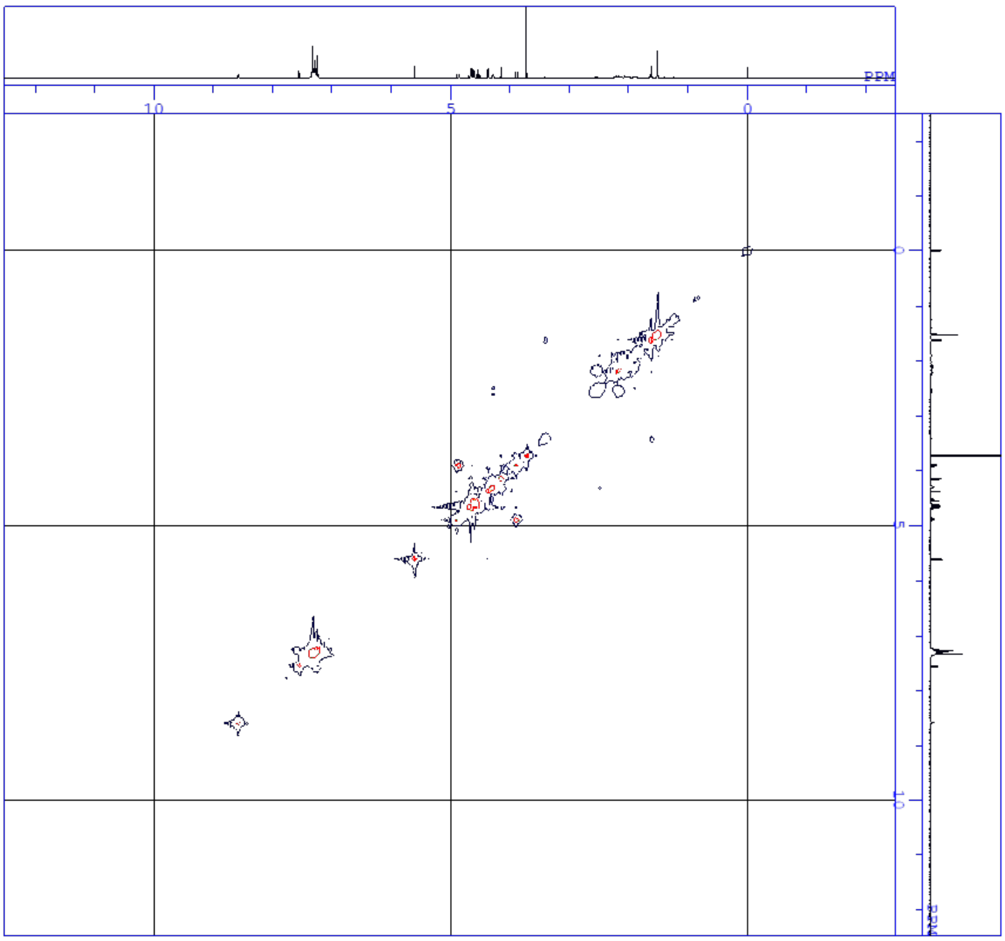

NOESY spectrum

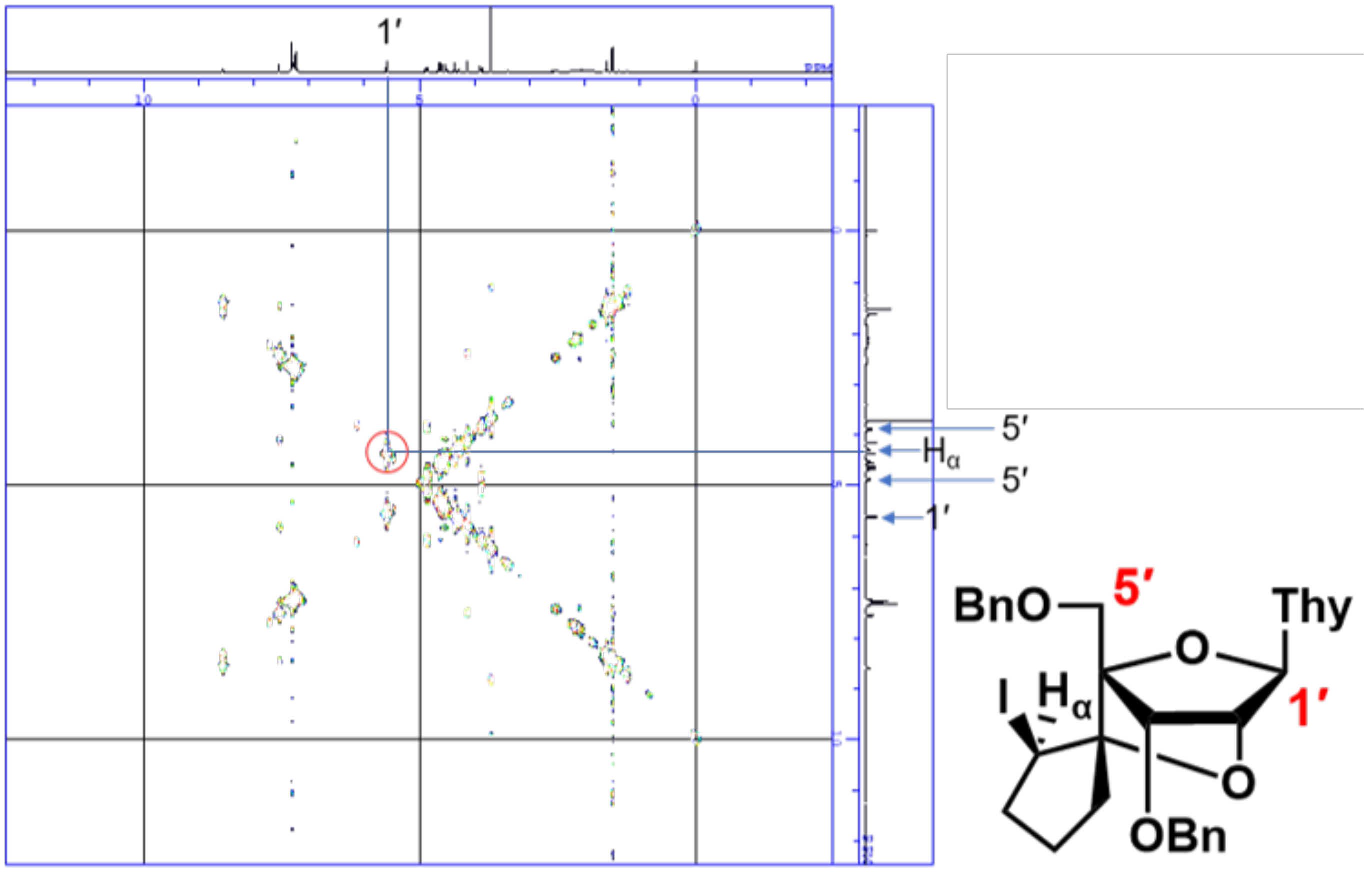

Compound **12** ($^{1}$H-NMR, $CDCl_3$, 500 MHz), ($^{1}$H-NMR, $CDCl_3$ by a $D_2O$ drop, 500 MHz), ($^{13}$C NMR, $CDCl_3$, 100.6 MHz)

Stereochemistry is unidentified.

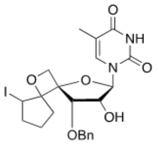


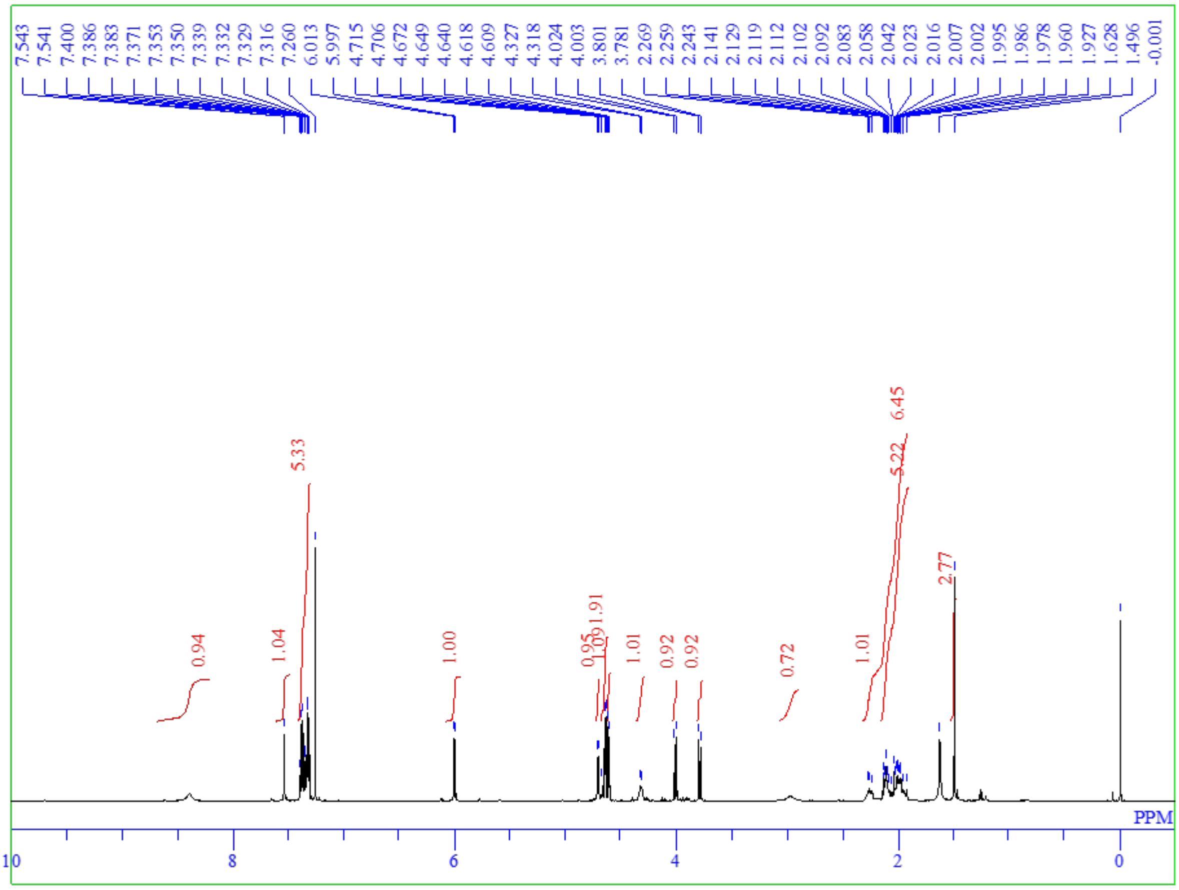


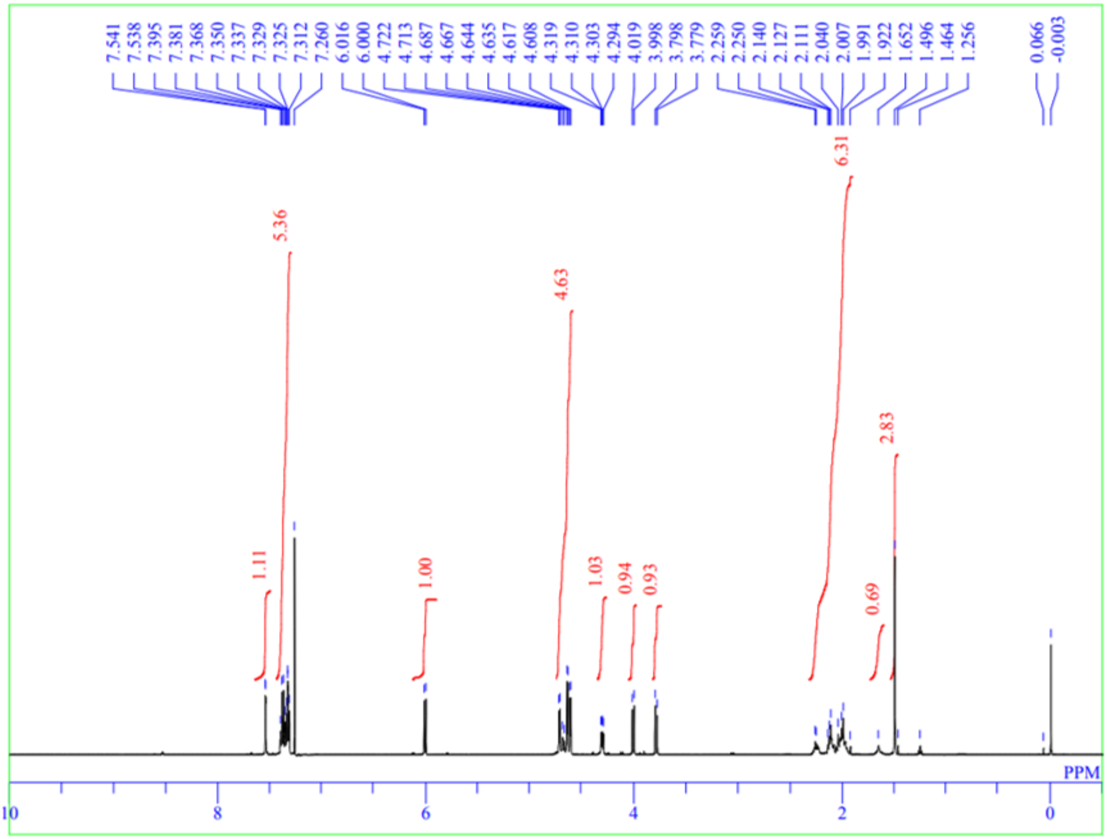

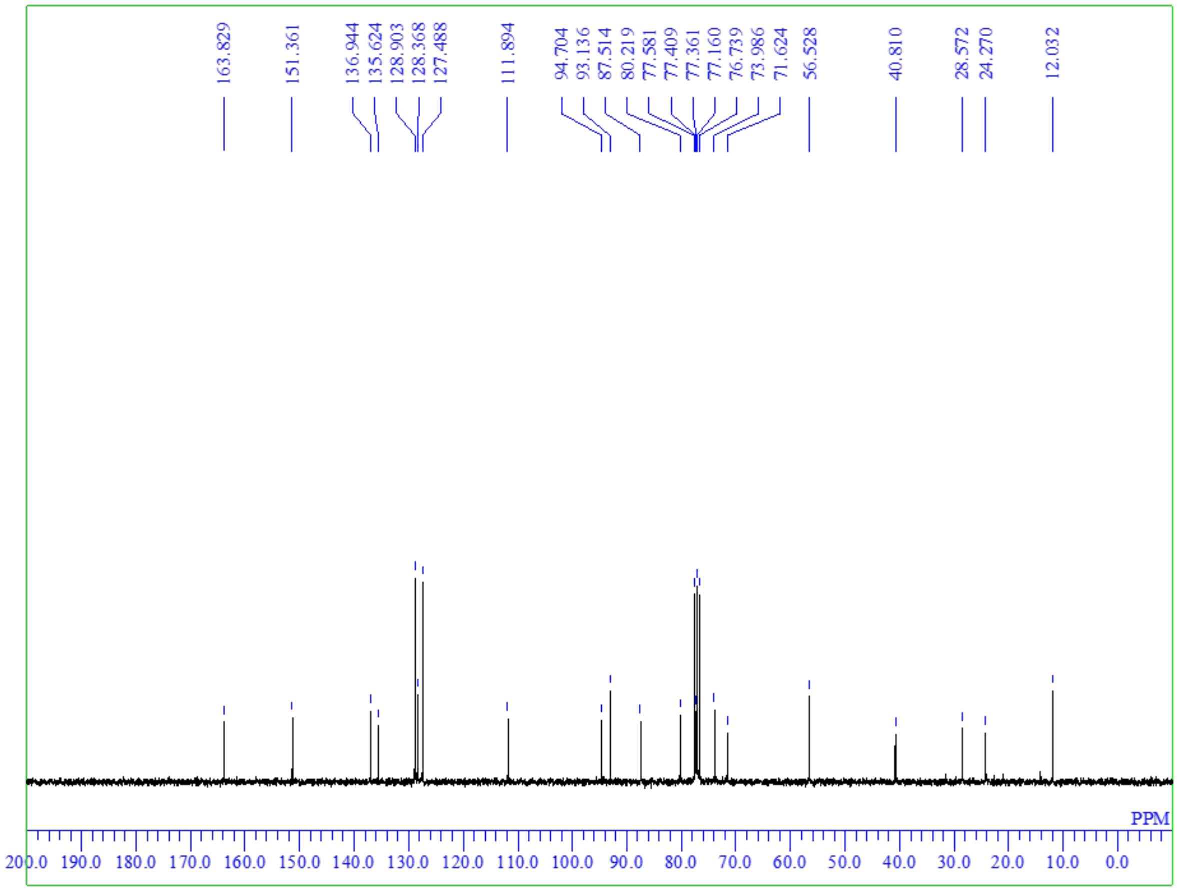


COSY spectrum

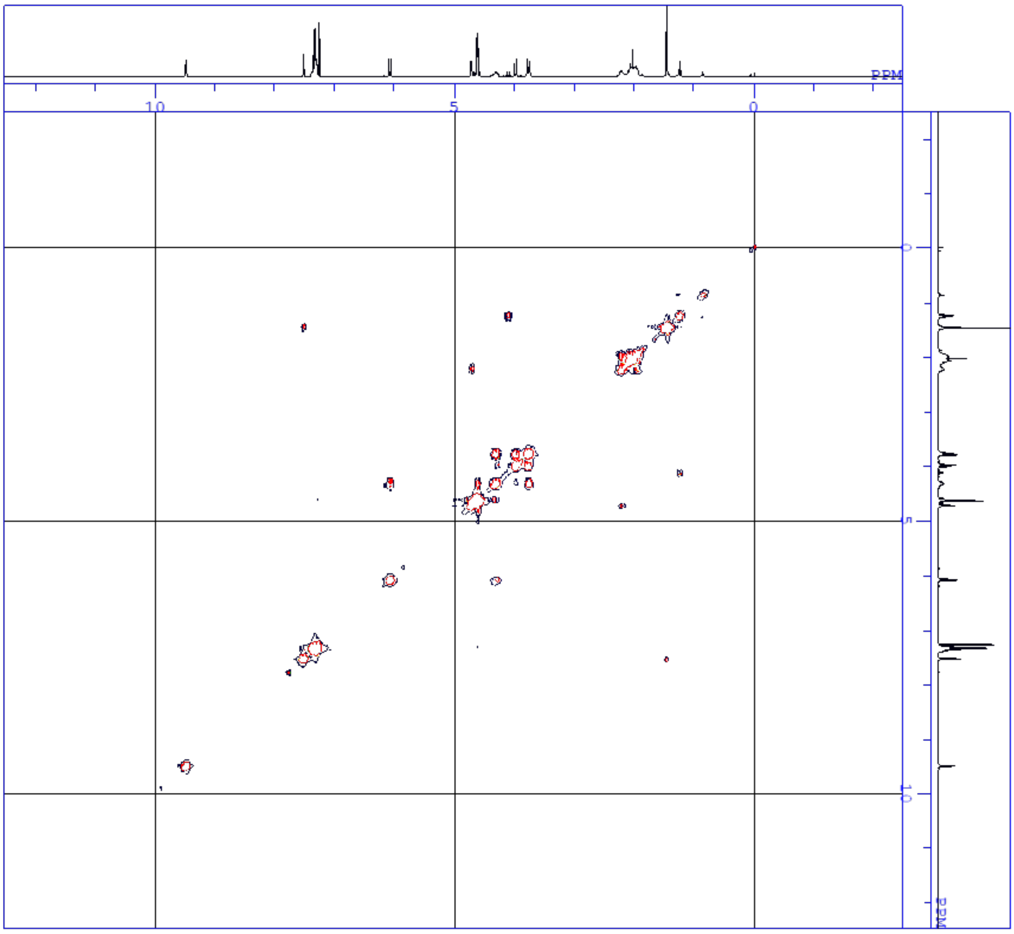

NOESY spectrum

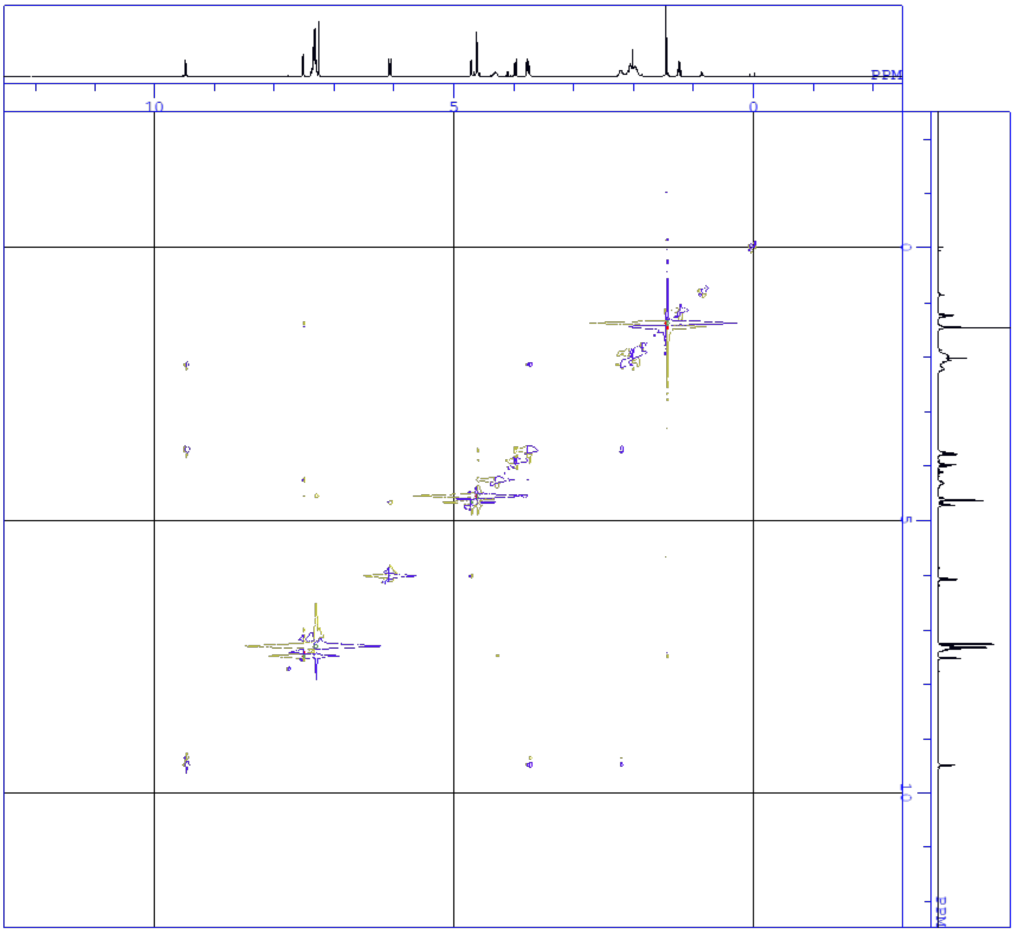

Compound **13** ($^1$H-NMR, $CDCl_3$, 500 MHz), ($^{13}$C NMR, $CDCl_3$, 125.8 MHz)

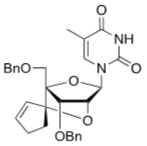


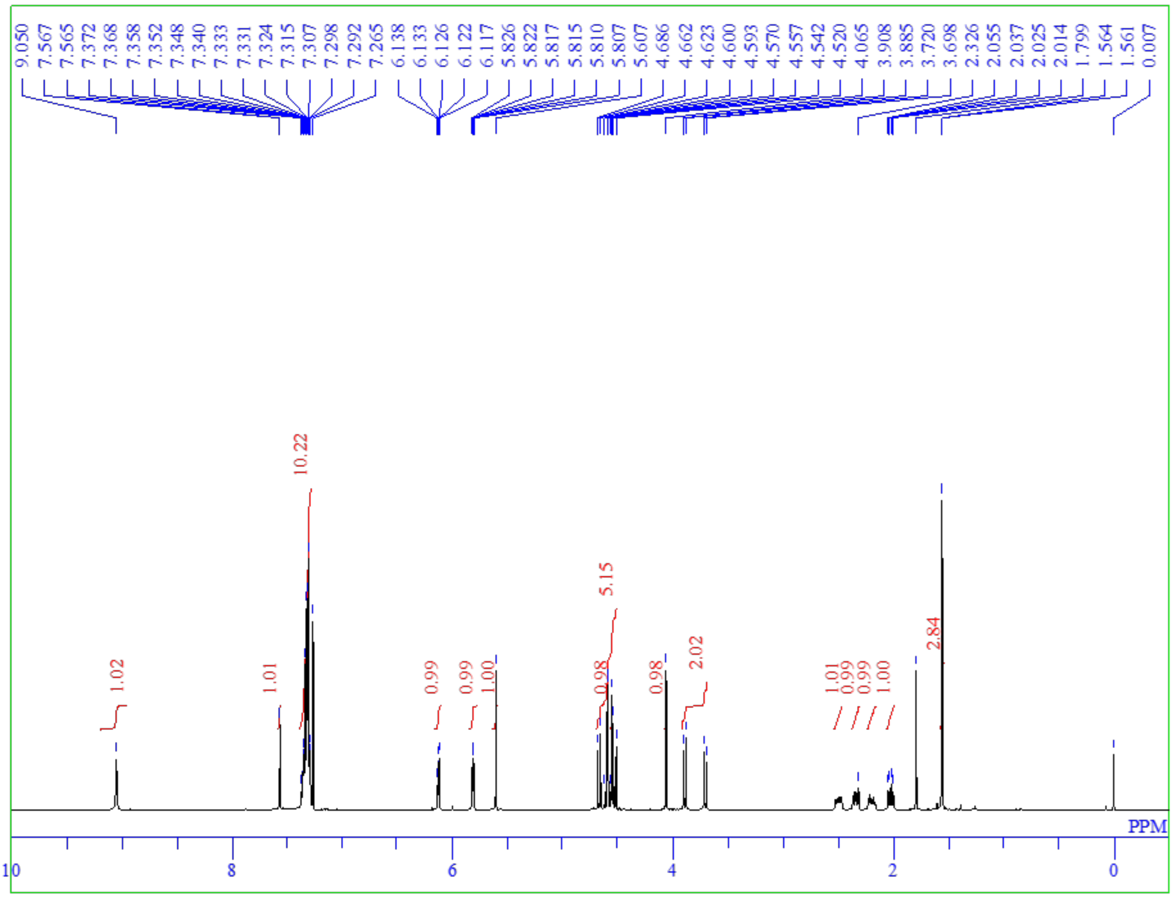


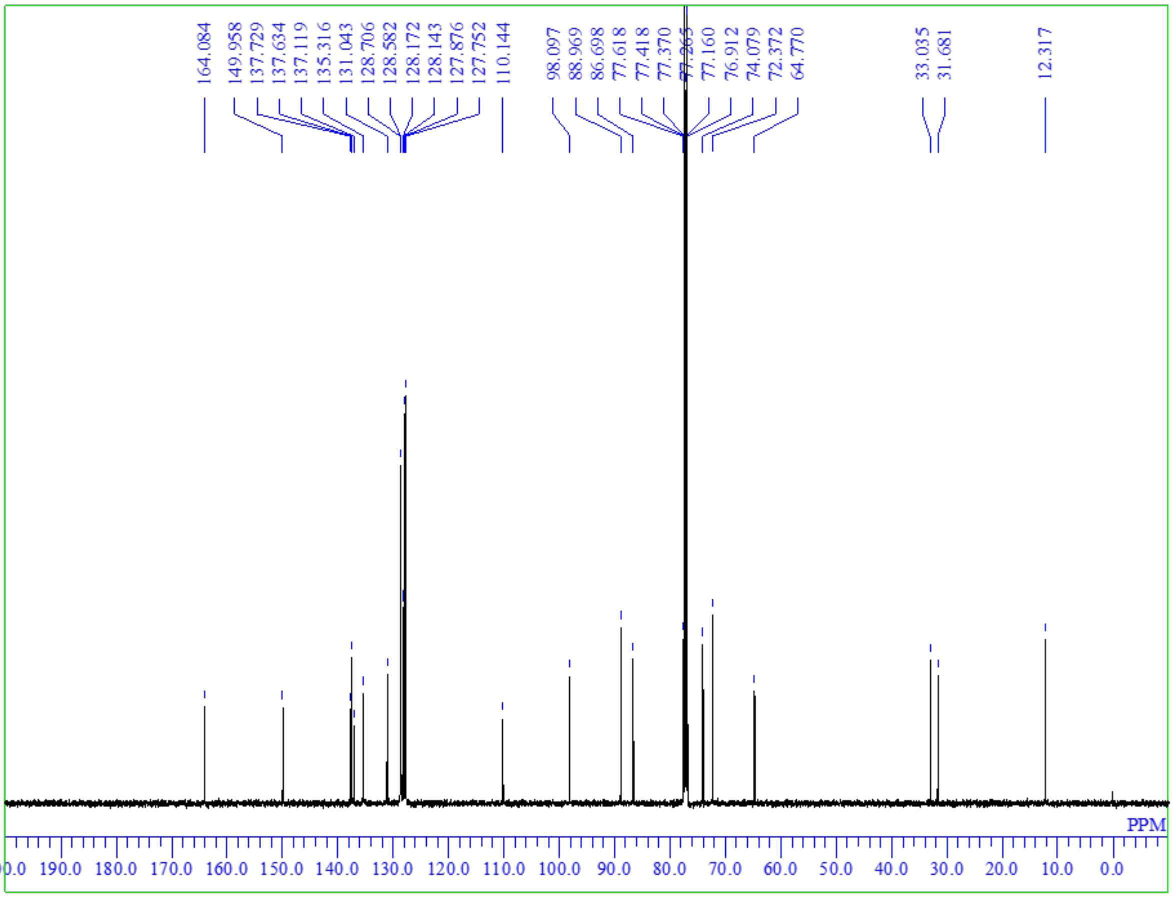

Compound **14** ($^{1}$H-NMR, $CDCl_3$, 300 MHz), ($^{13}$C NMR, $CDCl_3$, 75.6 MHz)

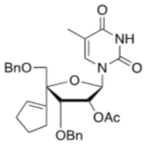


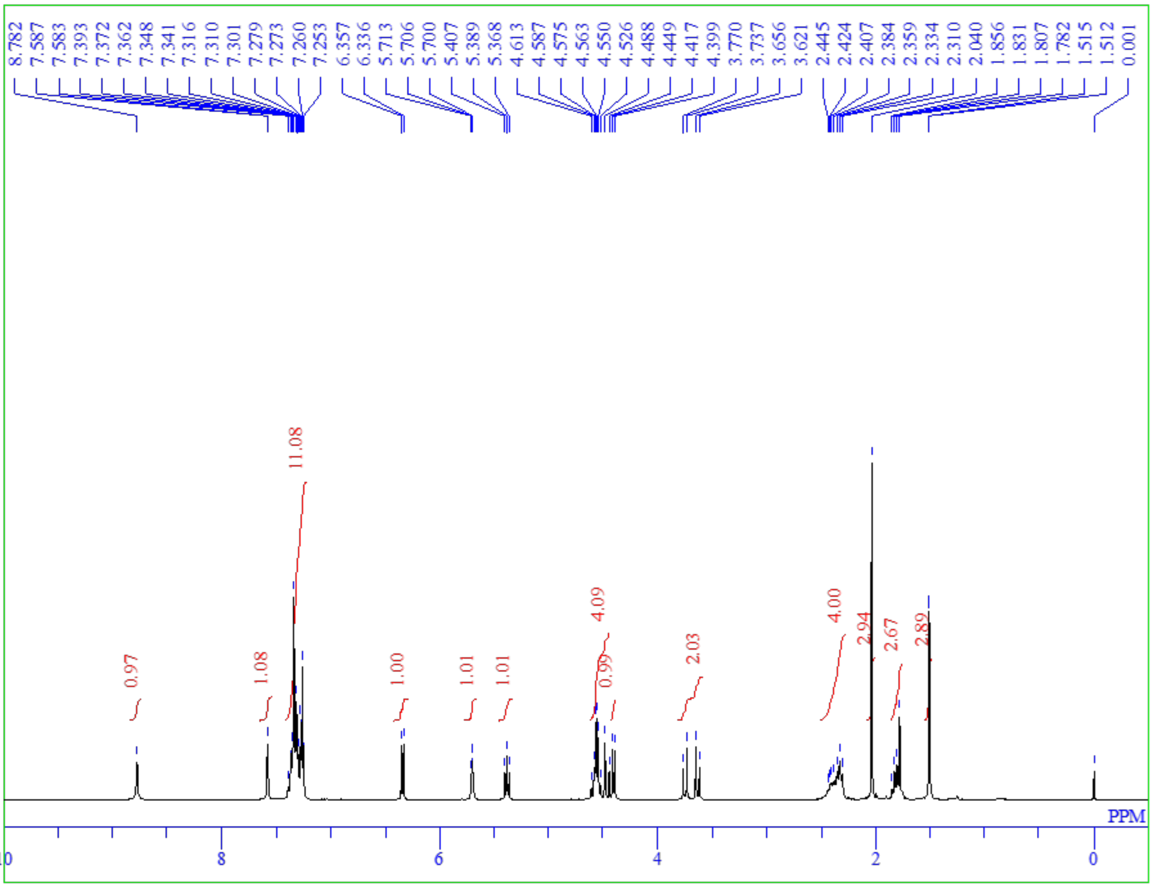


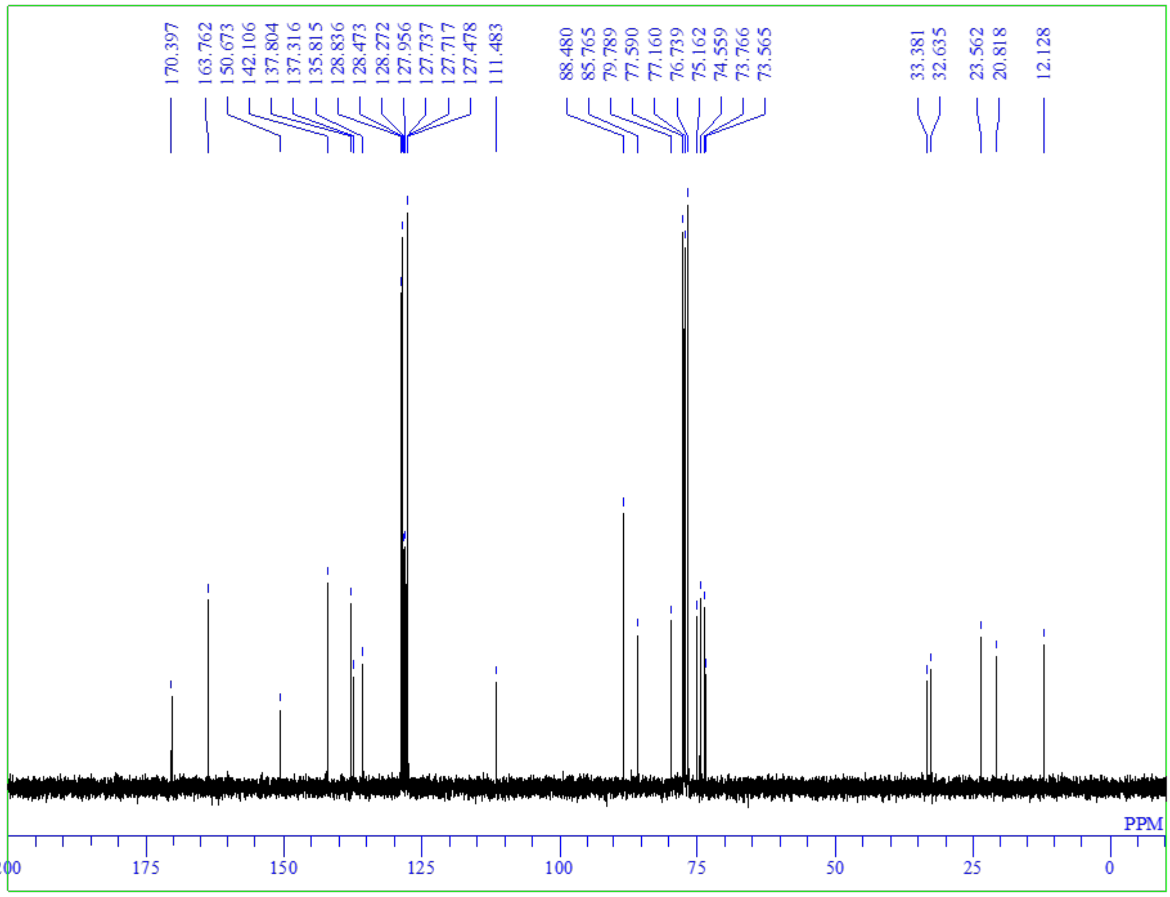

Compound **15** ($^{1}$H-NMR, $CDCl_3$, 500 MHz), ($^{13}$C NMR, $CDCl_3$, 125.8 MHz)

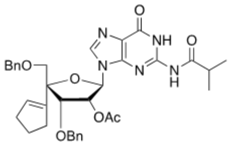


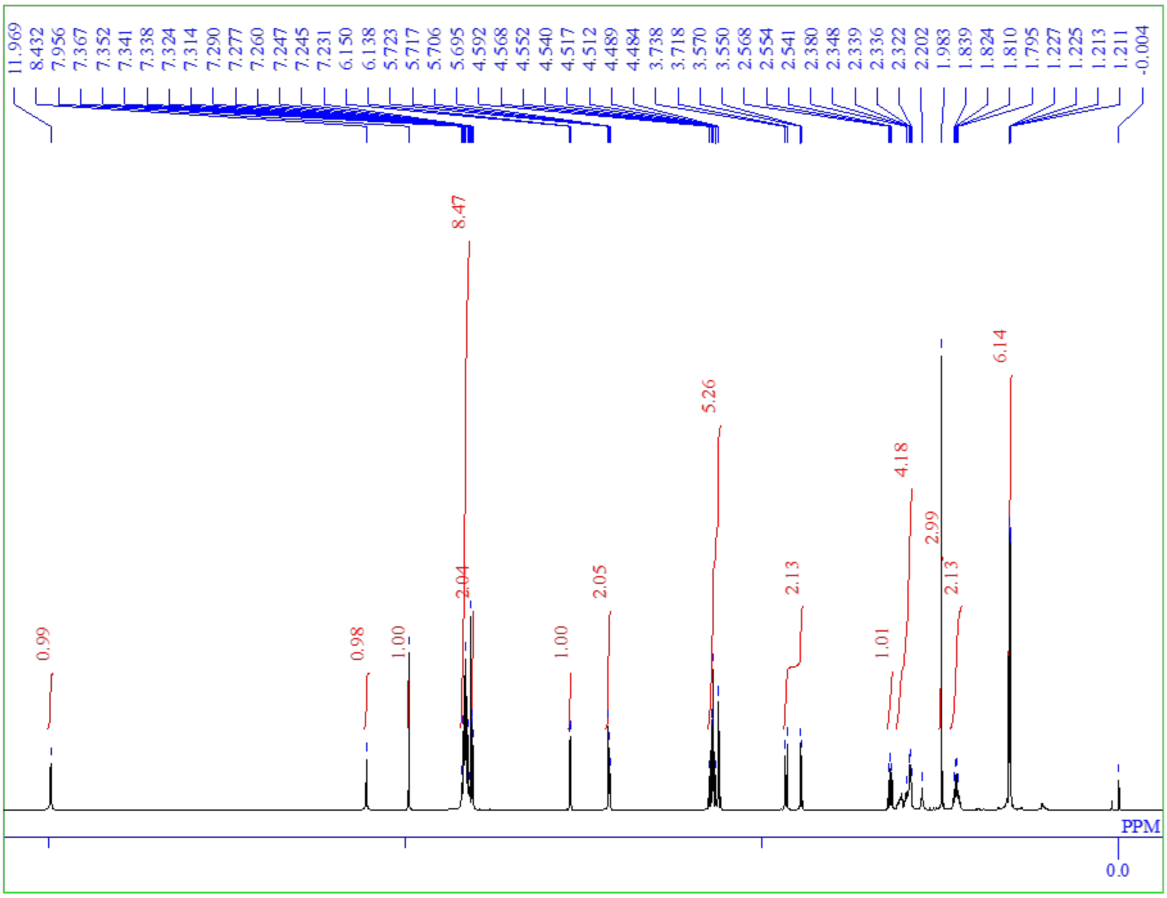


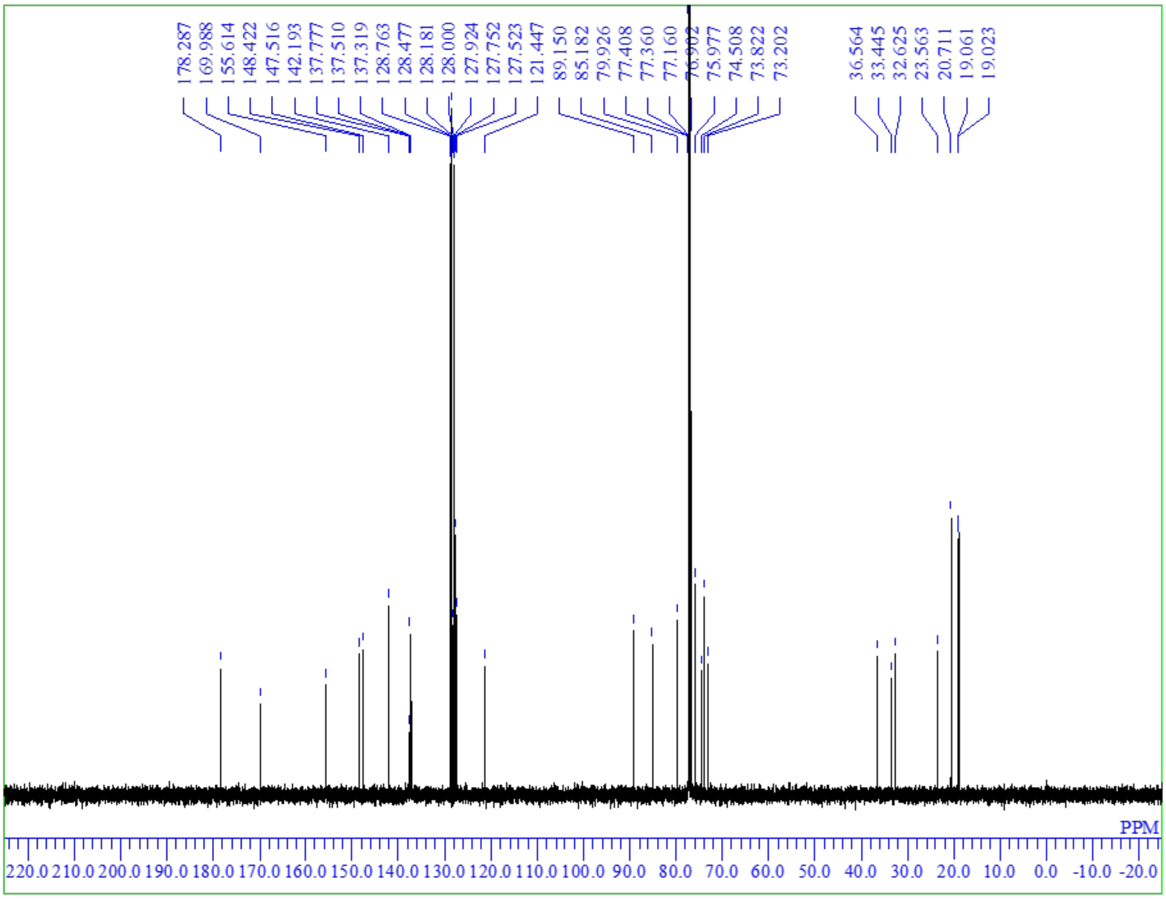

Compound **16** ($^1$H-NMR, $CDCl_3$, 300 MHz), ($^{13}$C NMR, $CDCl_3$, 75.6 MHz)

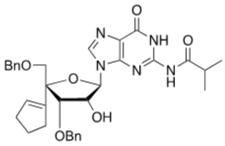


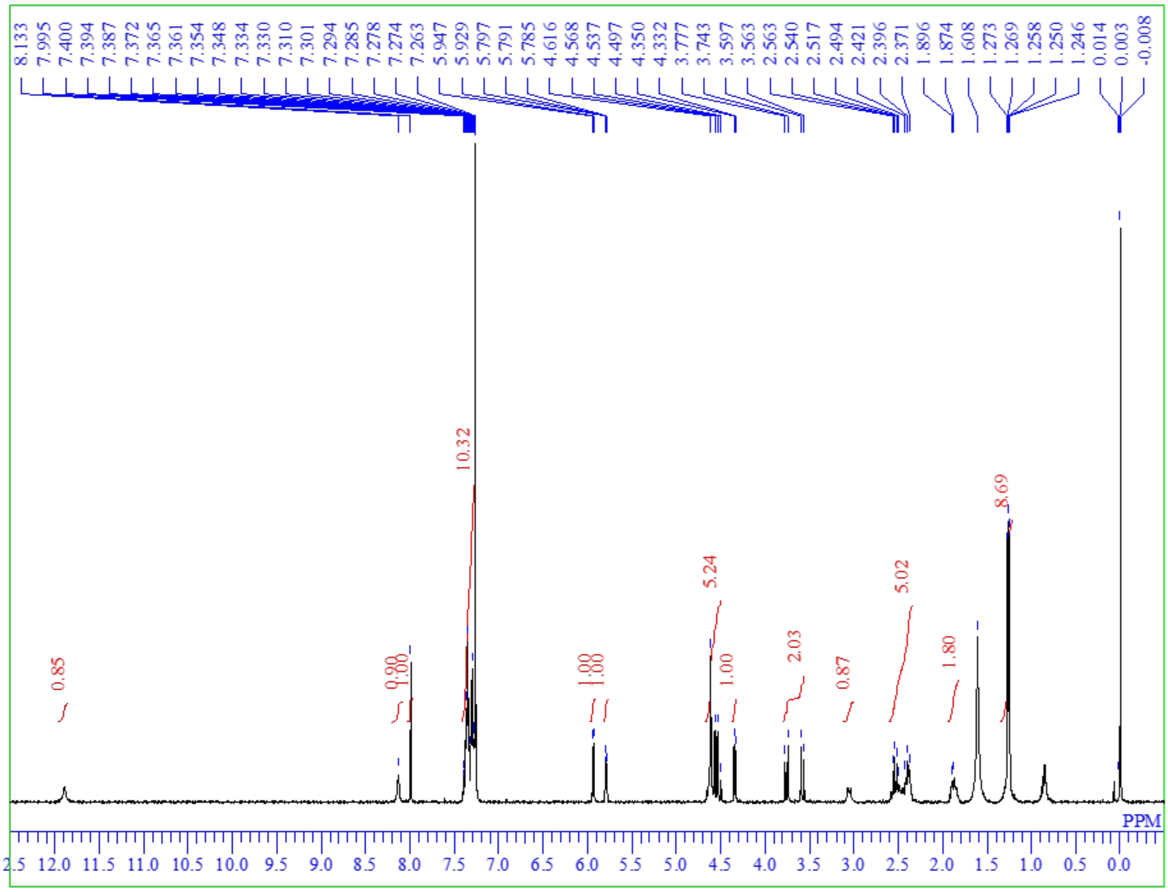


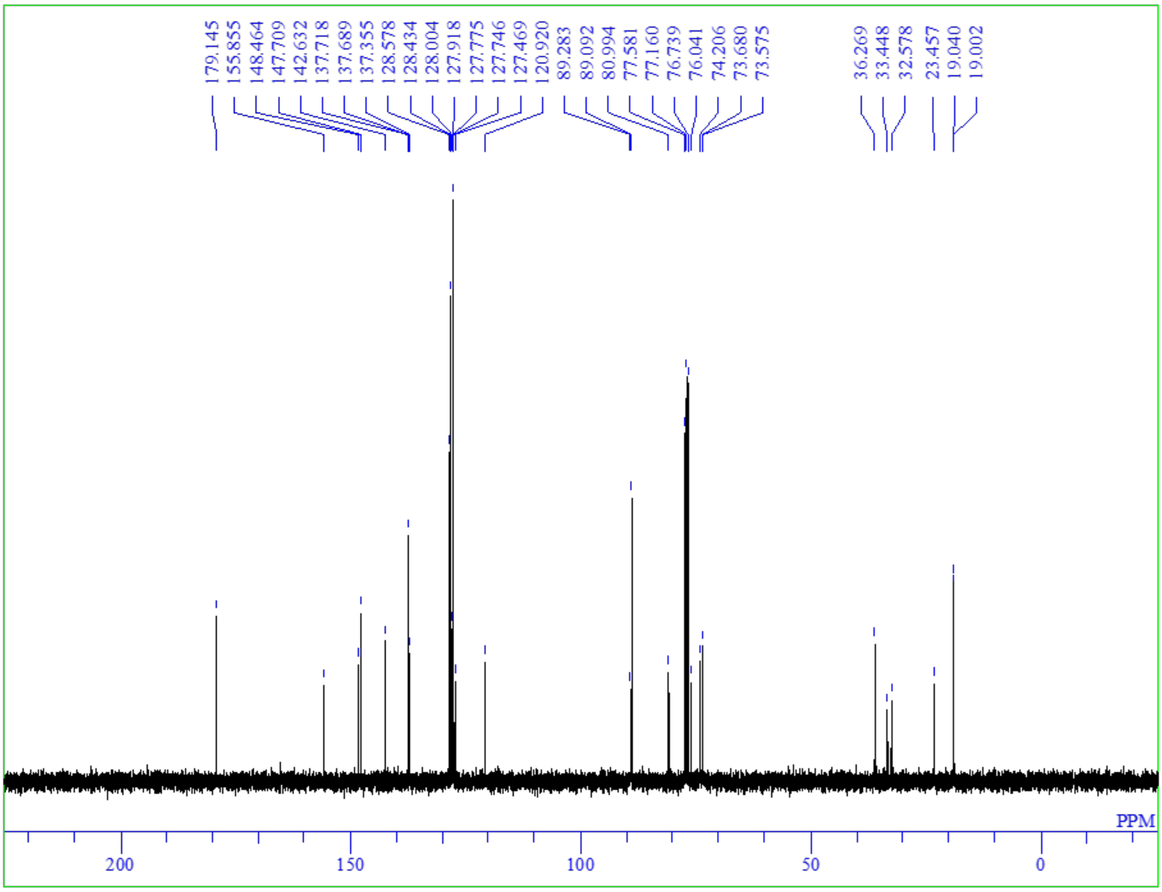

Compound **17** ($^{1}$H-NMR, $CDCl_3$, 300 MHz), ($^{13}$C NMR, $CDCl_3$, 75.6 MHz)

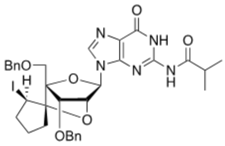


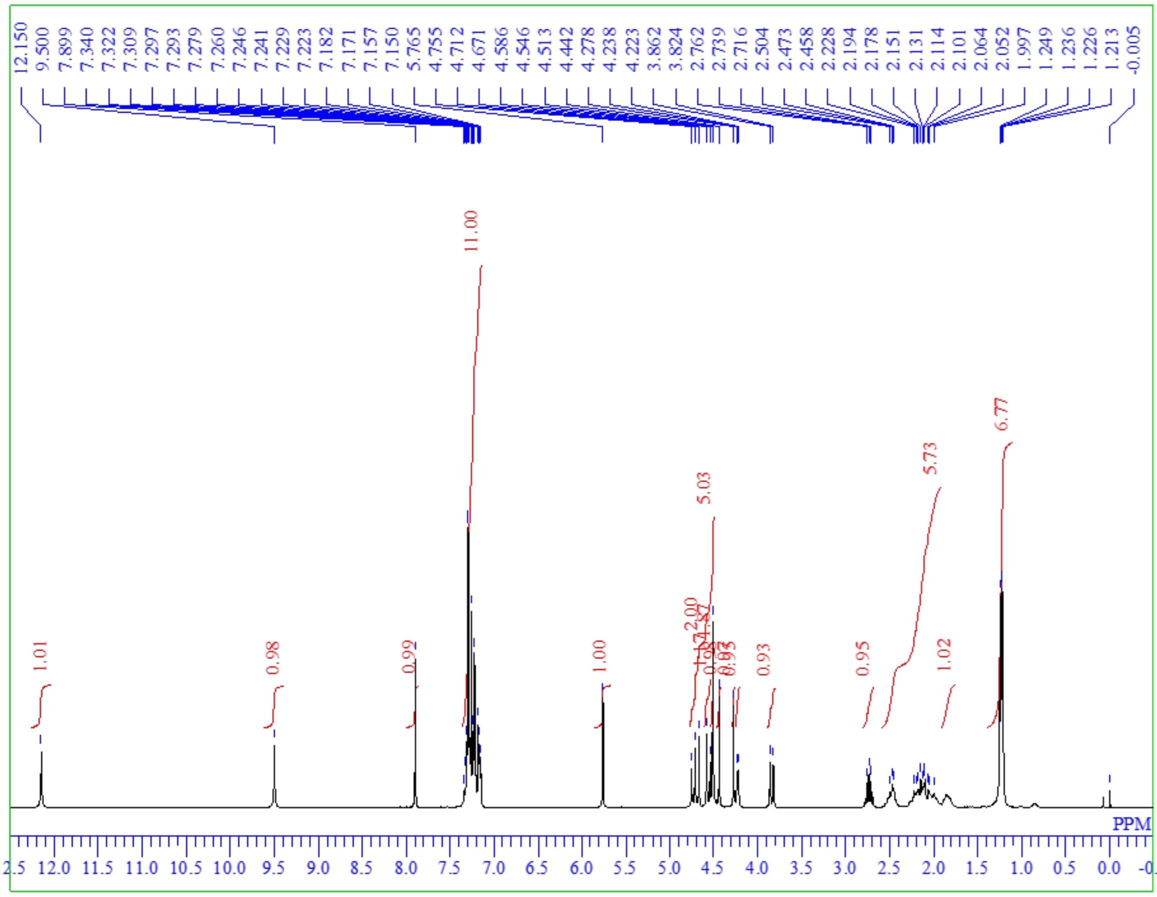


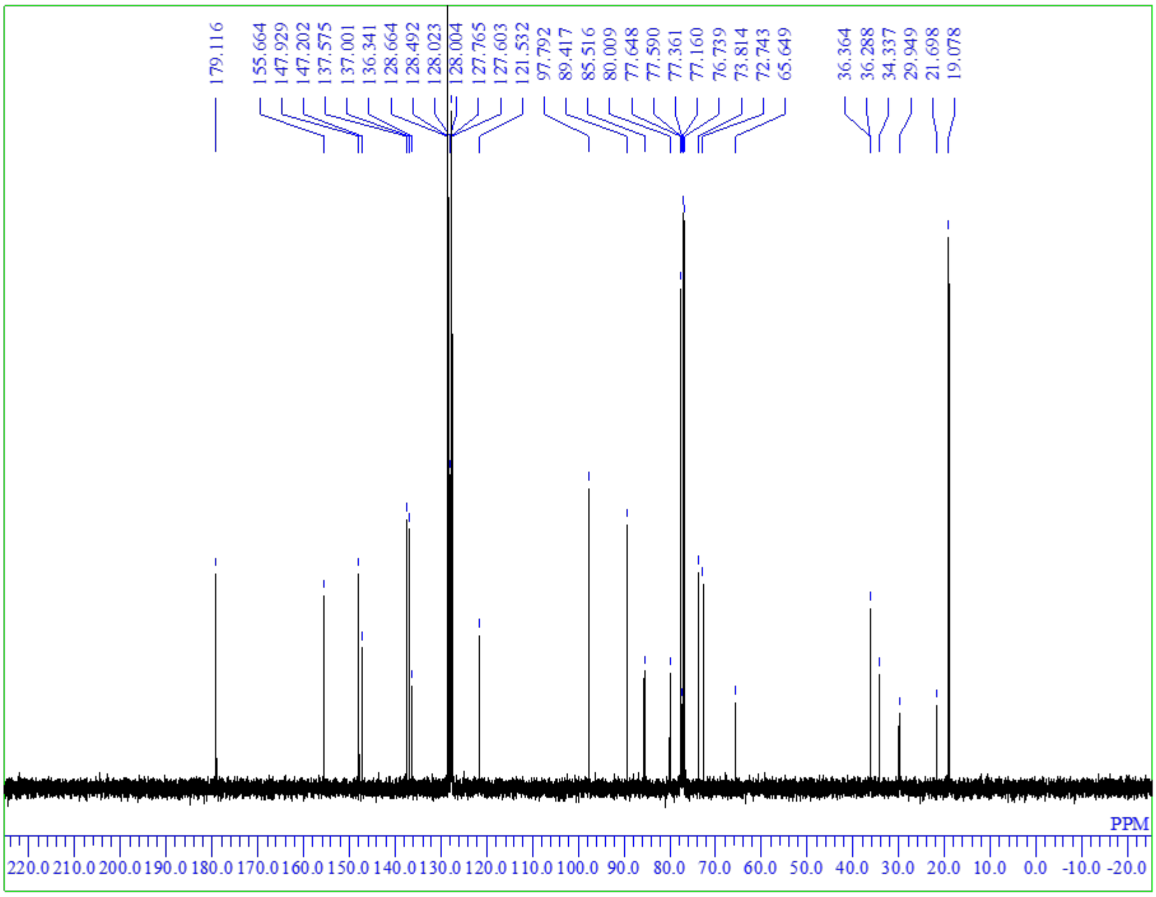

NOESY spectrum

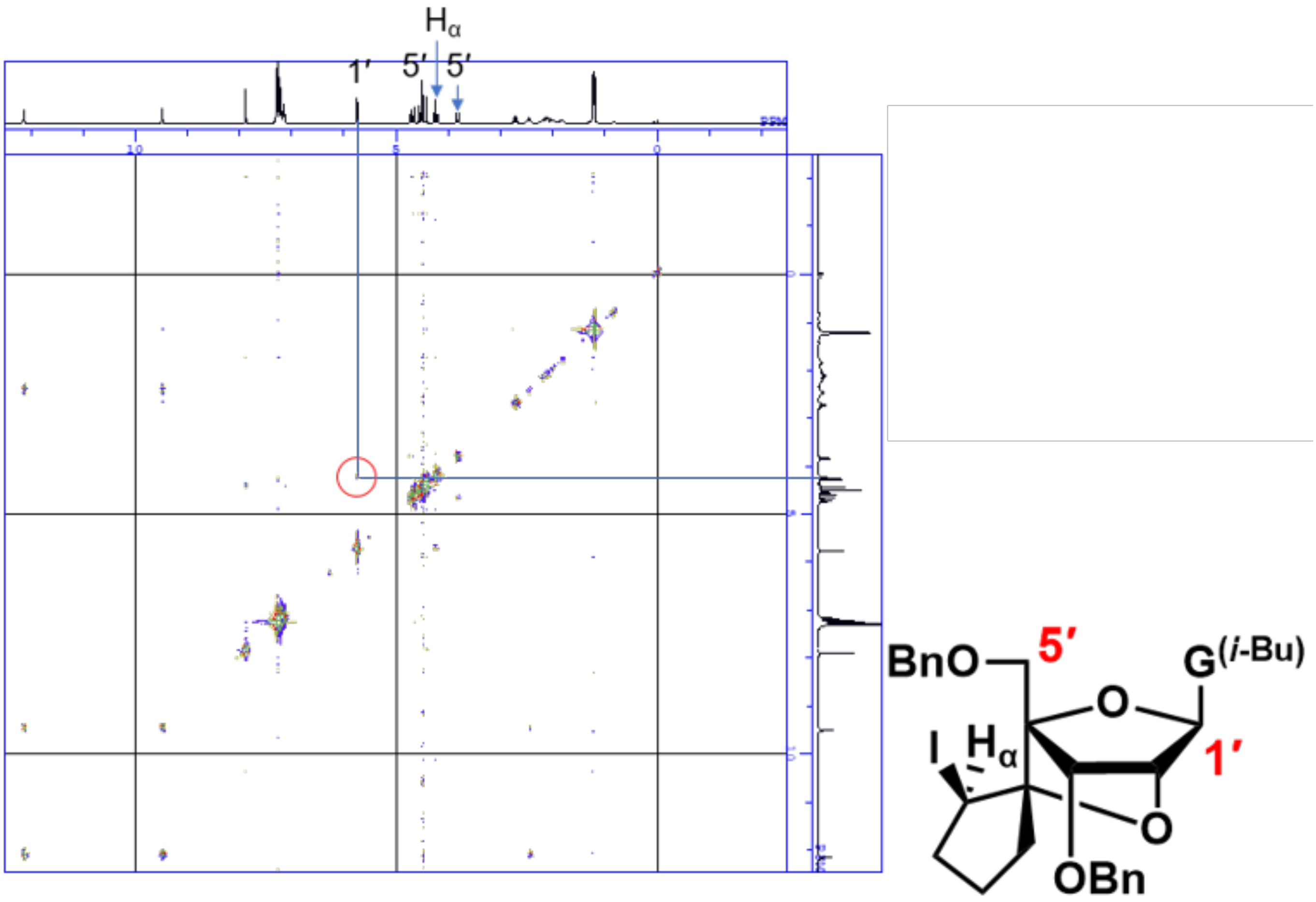

Compound **18** ($^{1}$H-NMR, $CDCl_3$, 500 MHz), ($^{13}$C NMR, $CDCl_3$, 125.8 MHz)

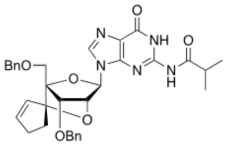

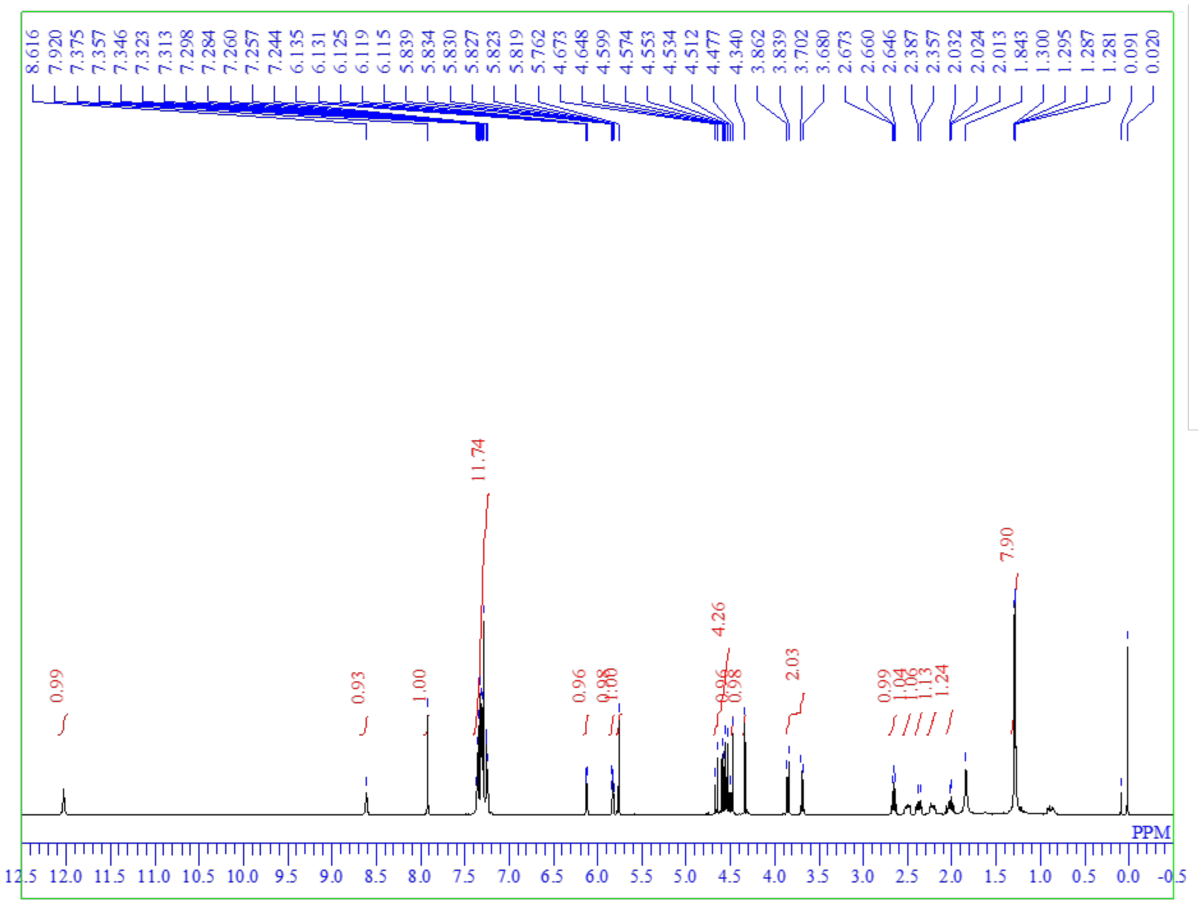

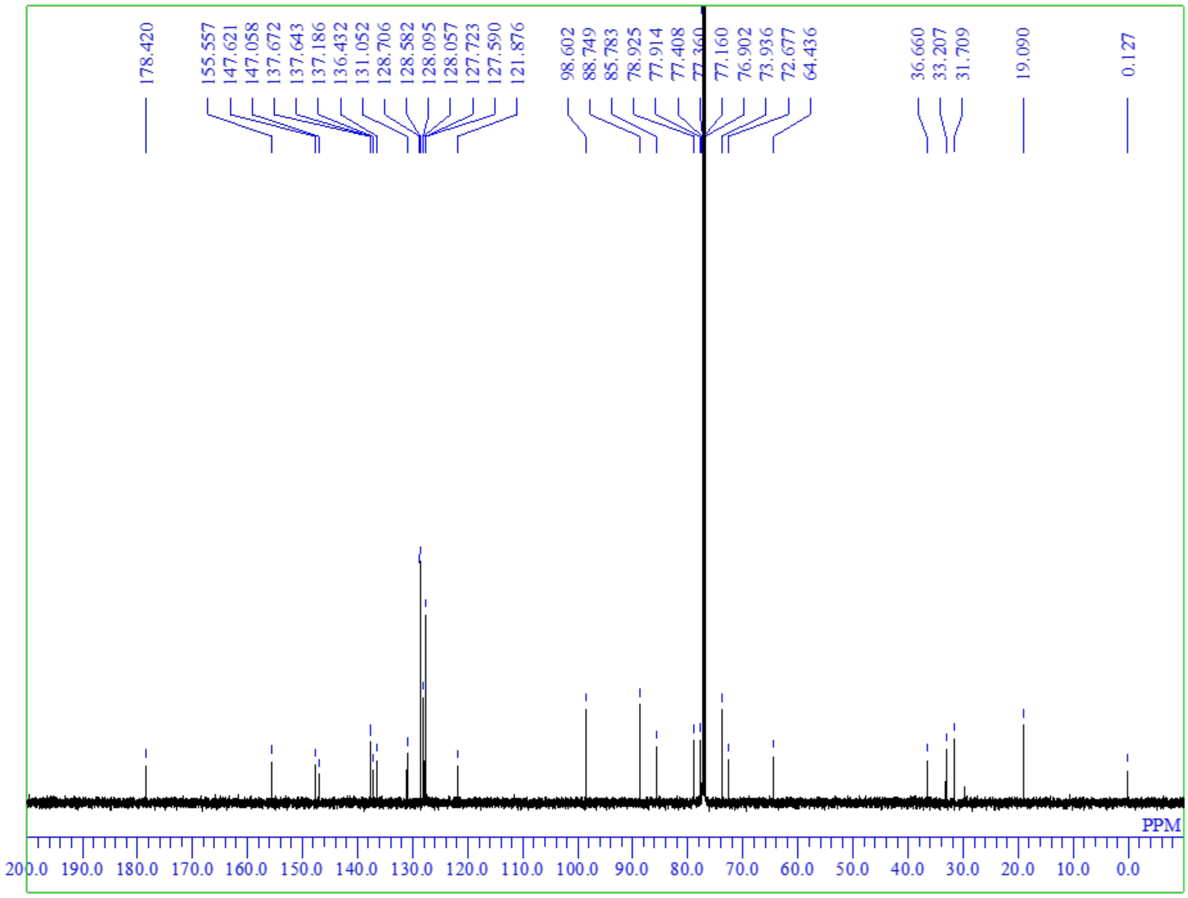

Compound **19** ($^{1}$H-NMR, $CD_3OD$, 300 MHz), ($^{13}$C NMR, $CD_3OD$, 125.8 MHz)

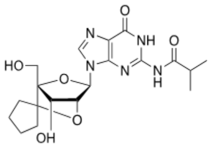

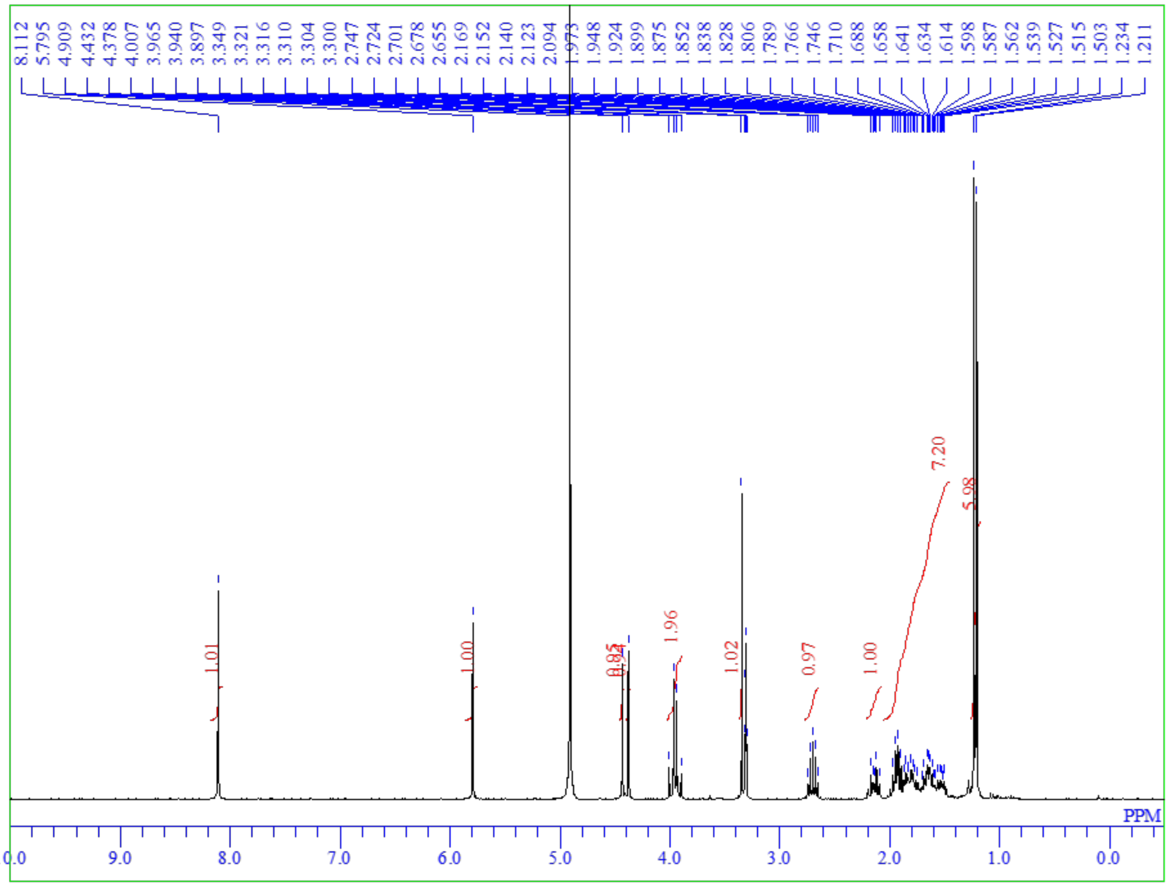


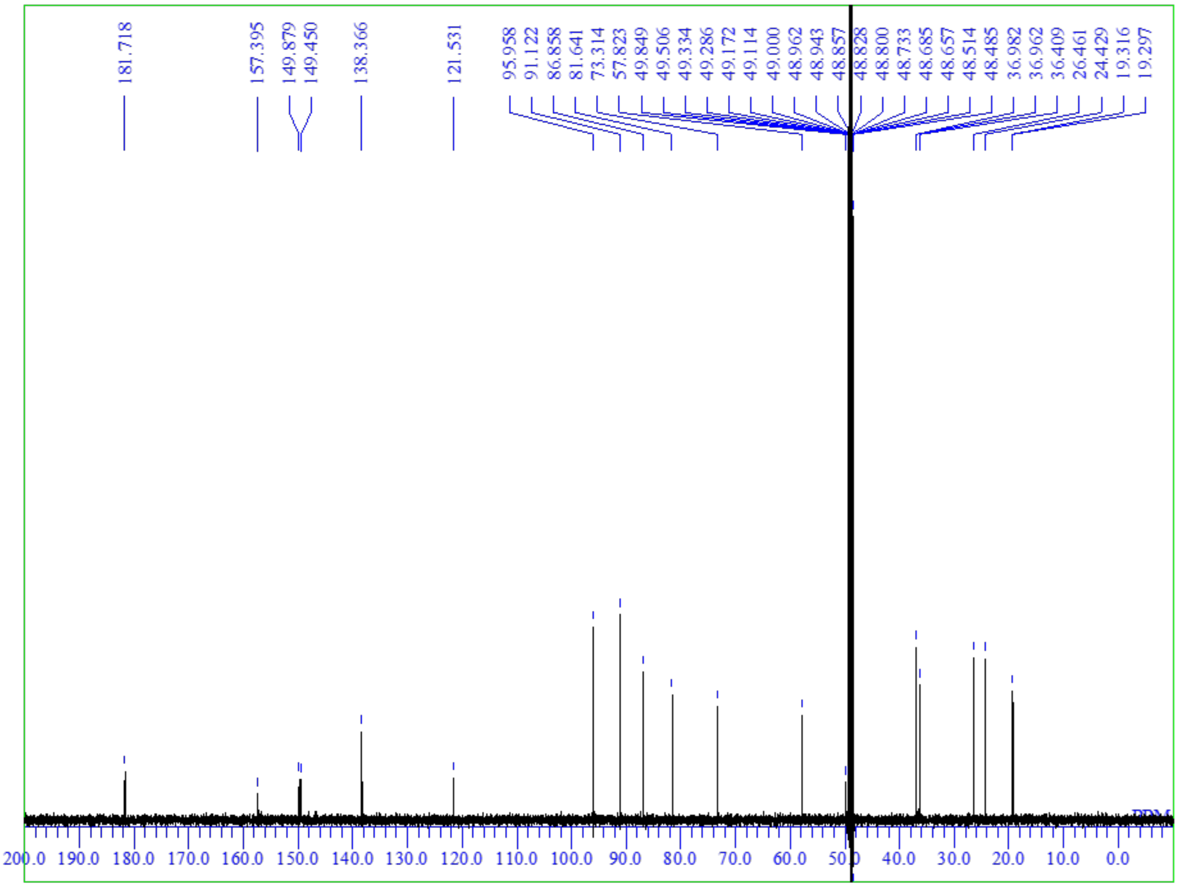

Compound **20** ($^{1}$H-NMR, $CDCl_3$, 300 MHz), ($^{13}$C NMR, $CDCl_3$, 125.8 MHz)

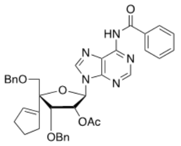


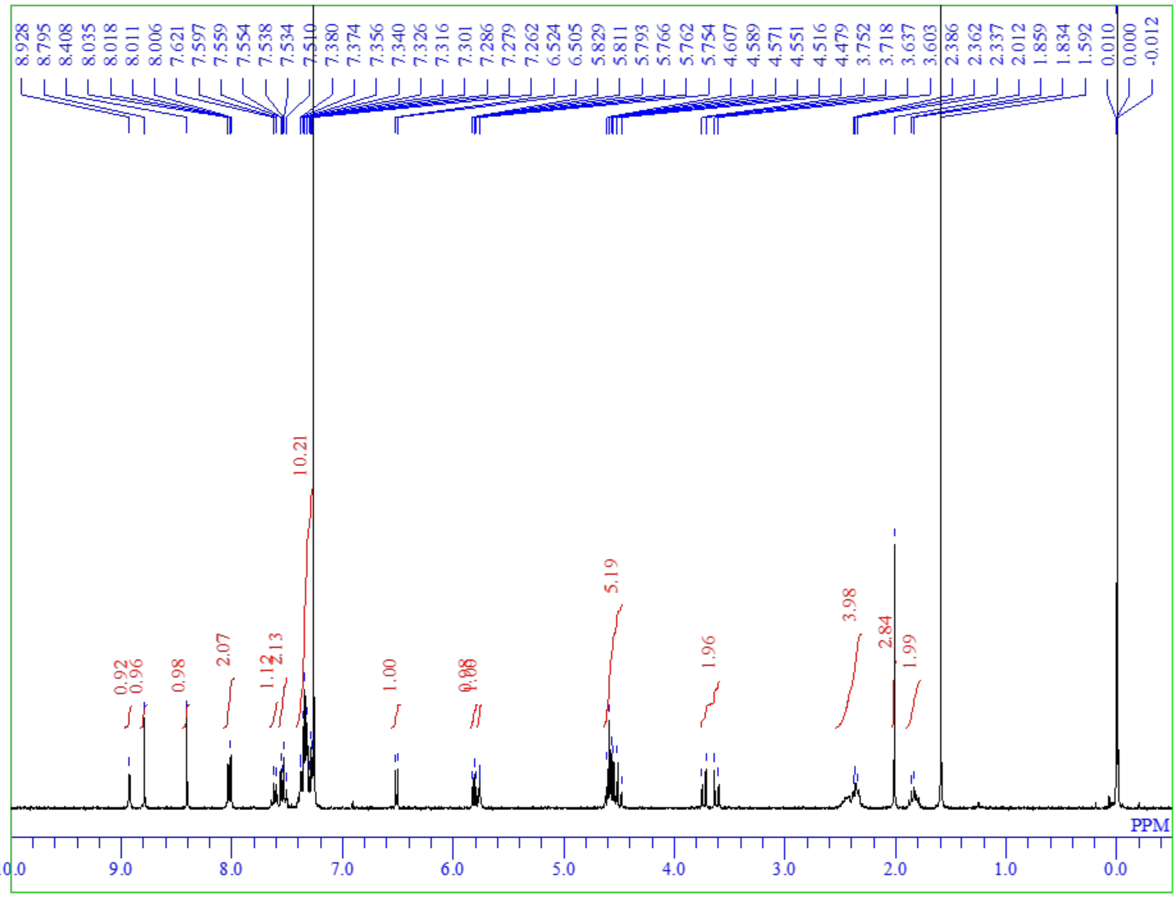


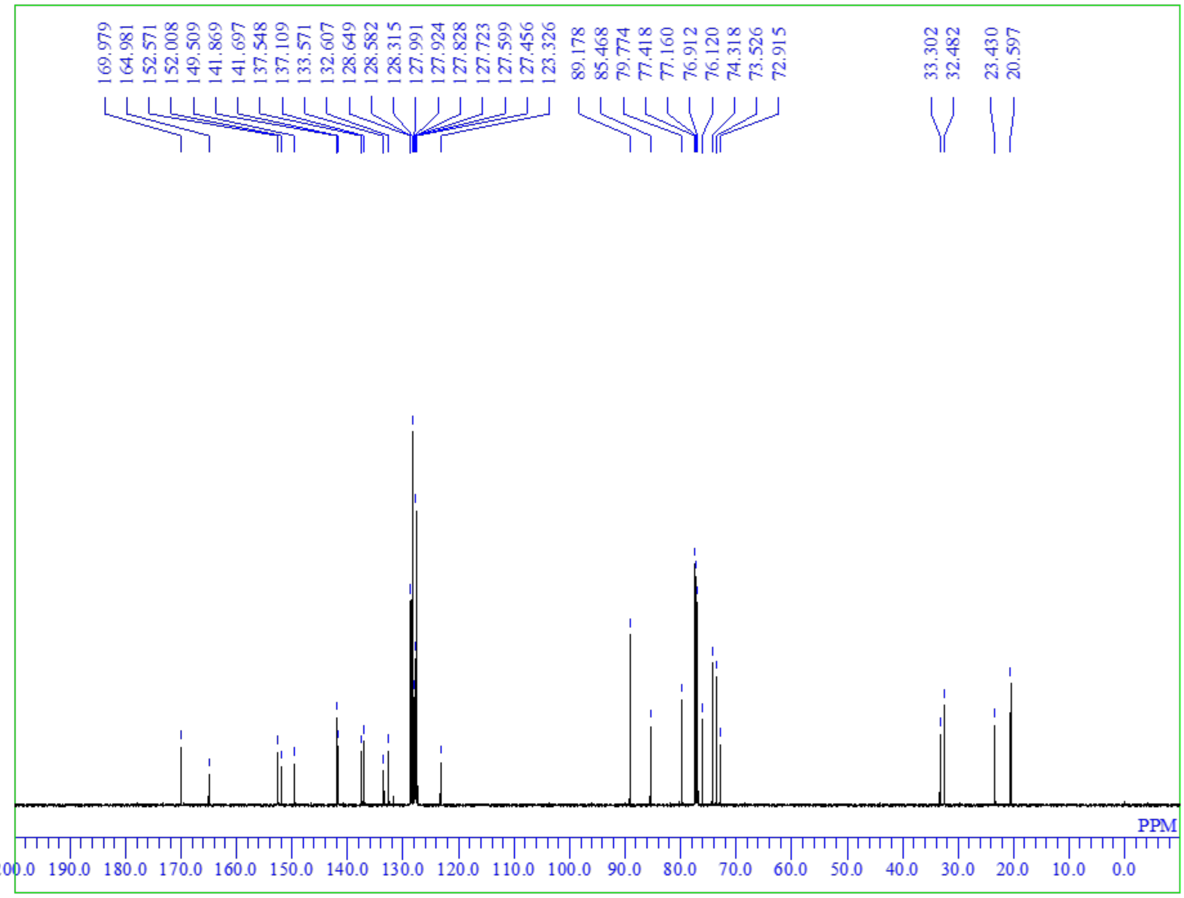

Compound **21** ($^1$H-NMR, $CDCl_3$, 500 MHz), ($^{13}$C NMR, $CDCl_3$, 125.8 MHz)

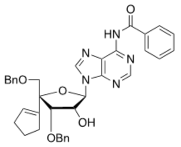


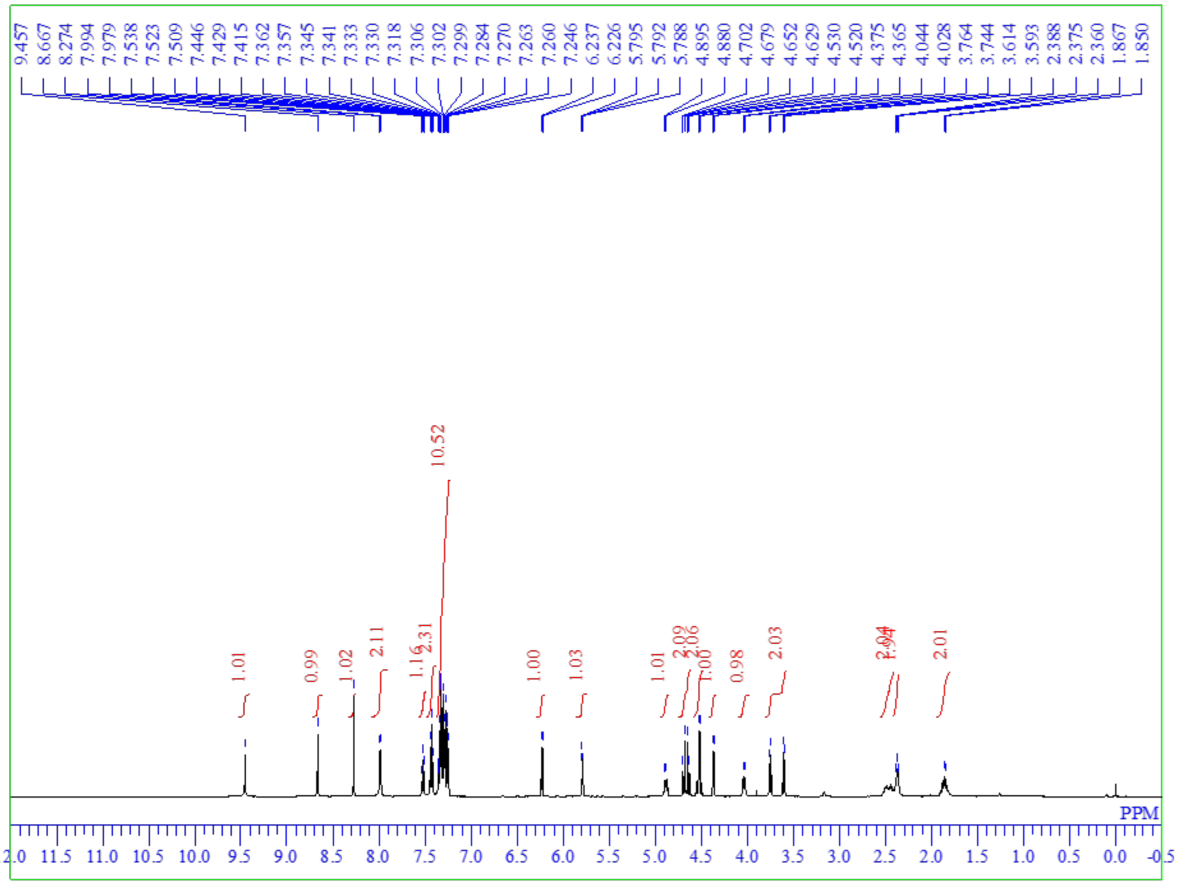


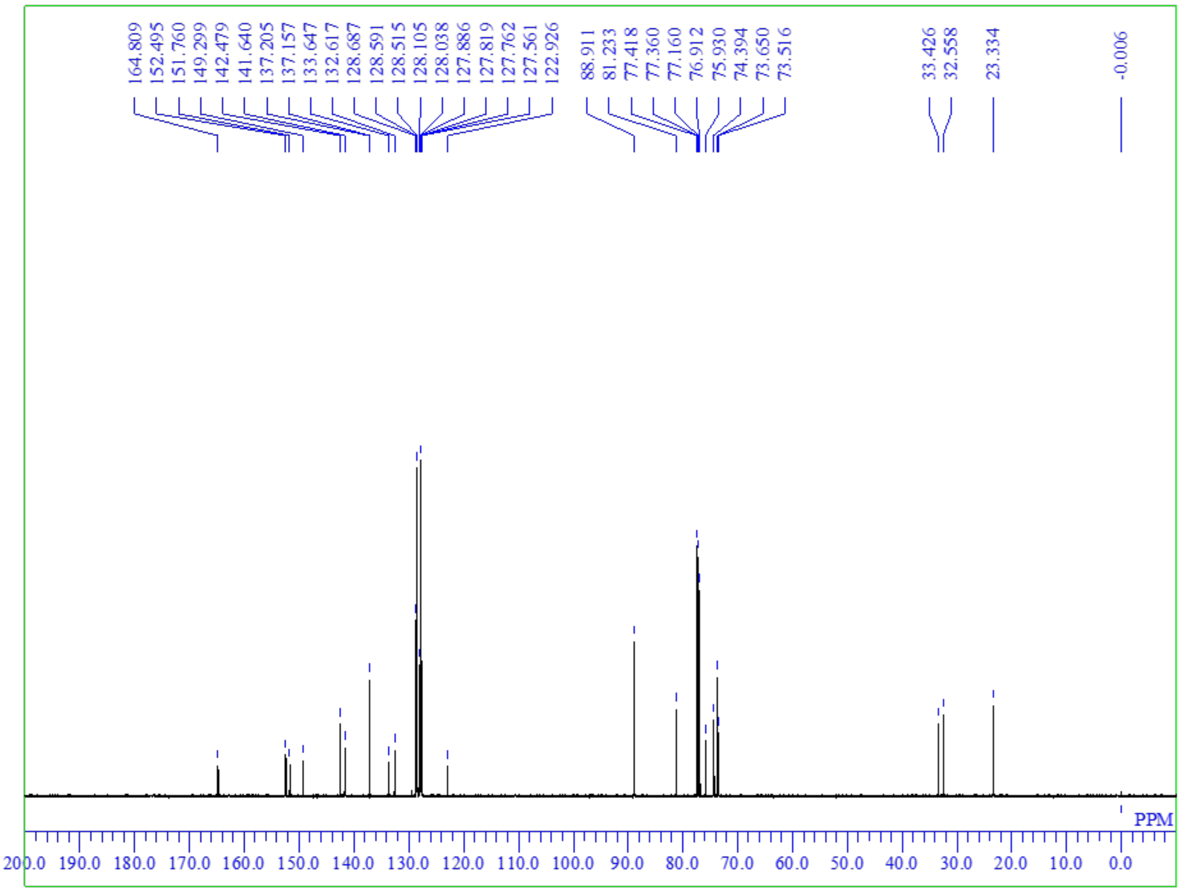

Compound **22** ($^{1}$H-NMR, $CDCl_3$, 500 MHz), ($^{13}$C NMR, $CDCl_3$, 125.8 MHz)

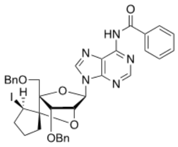


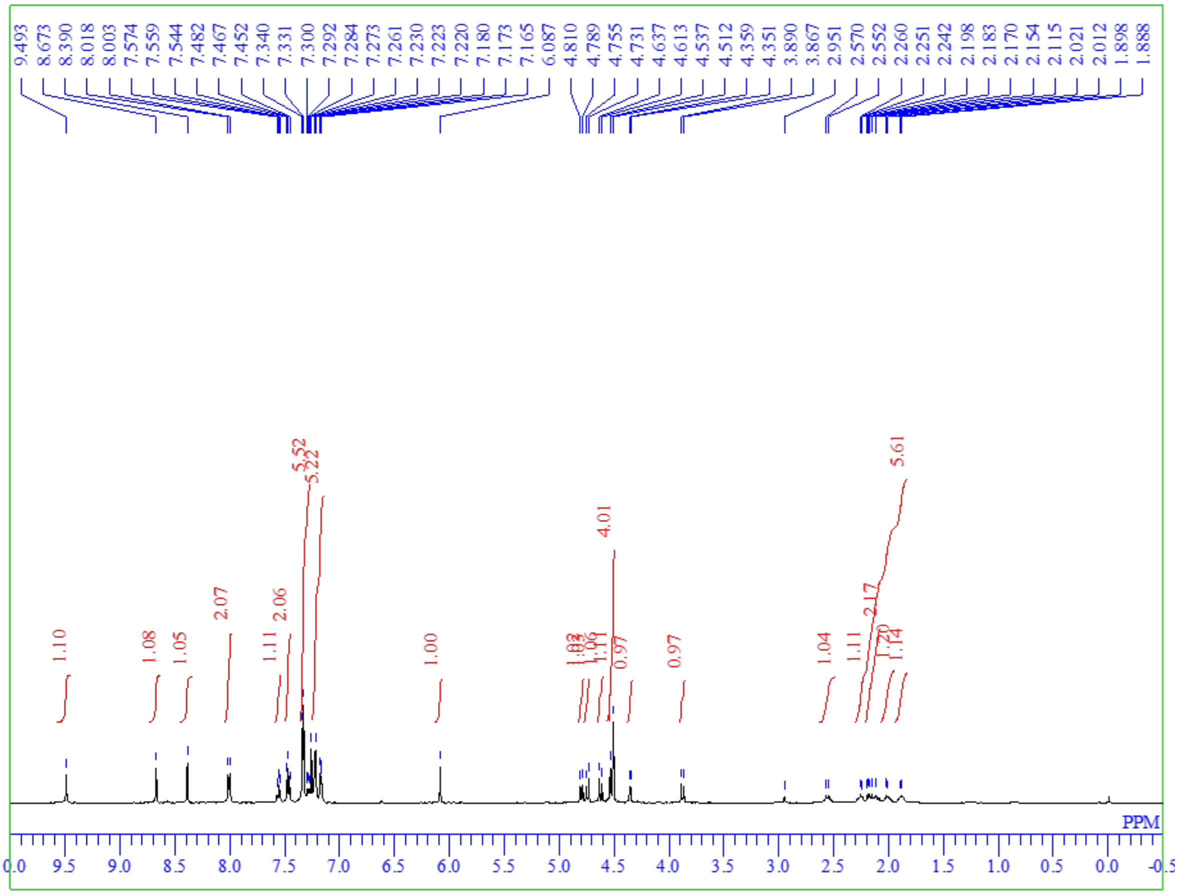


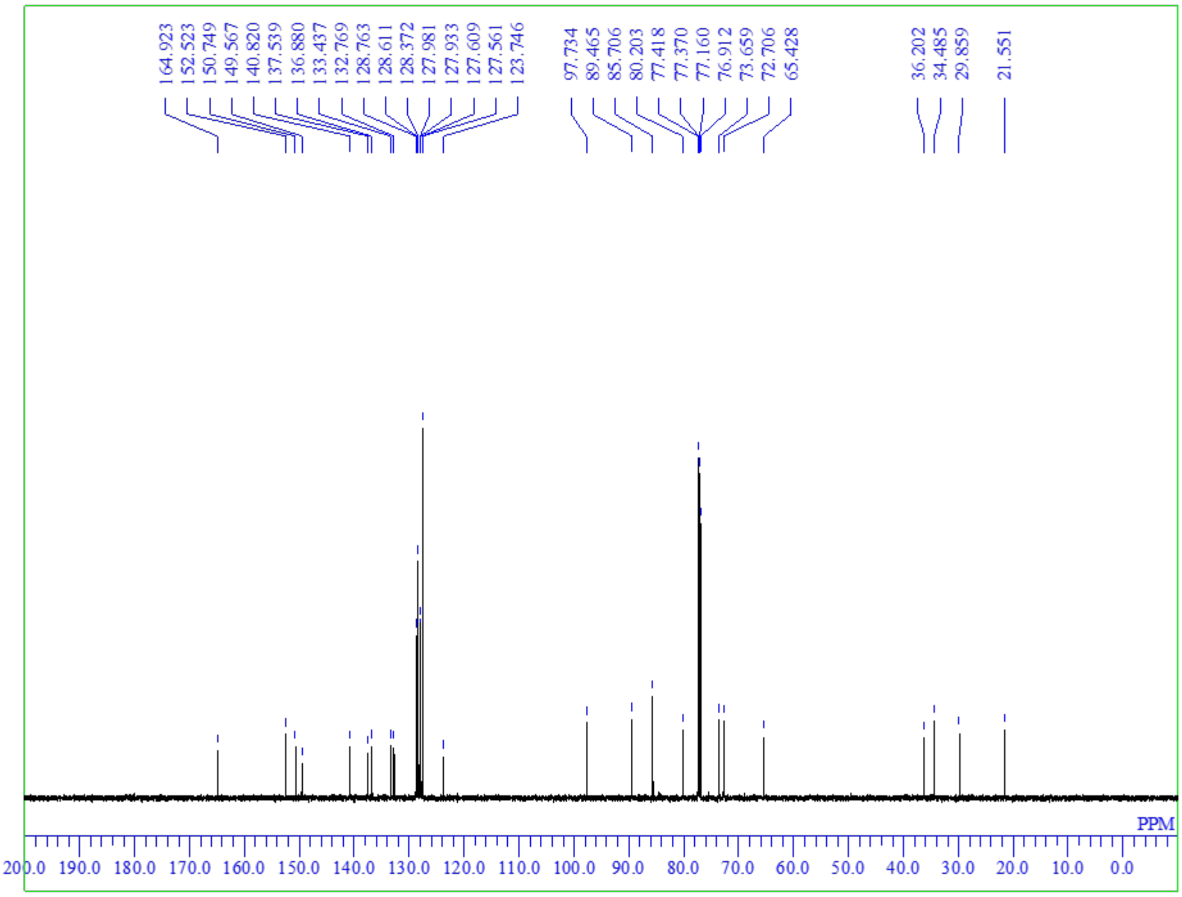

NOESY spectrum

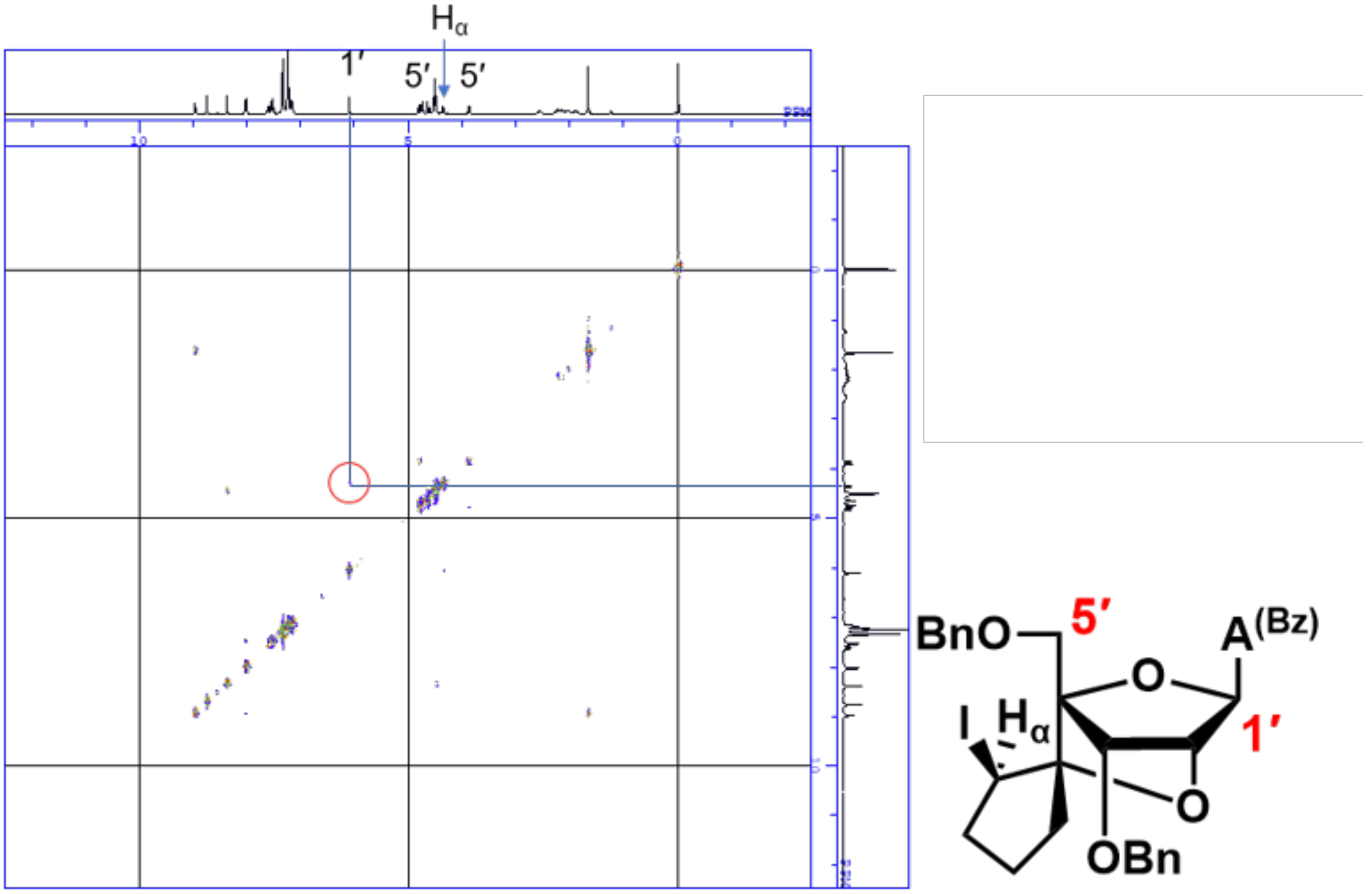

Compound **23** ($^1$H-NMR, $CDCl_3$, 300 MHz), ($^{13}$C NMR, $CDCl_3$, 75.6 MHz)

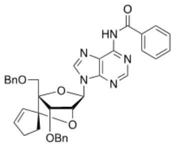


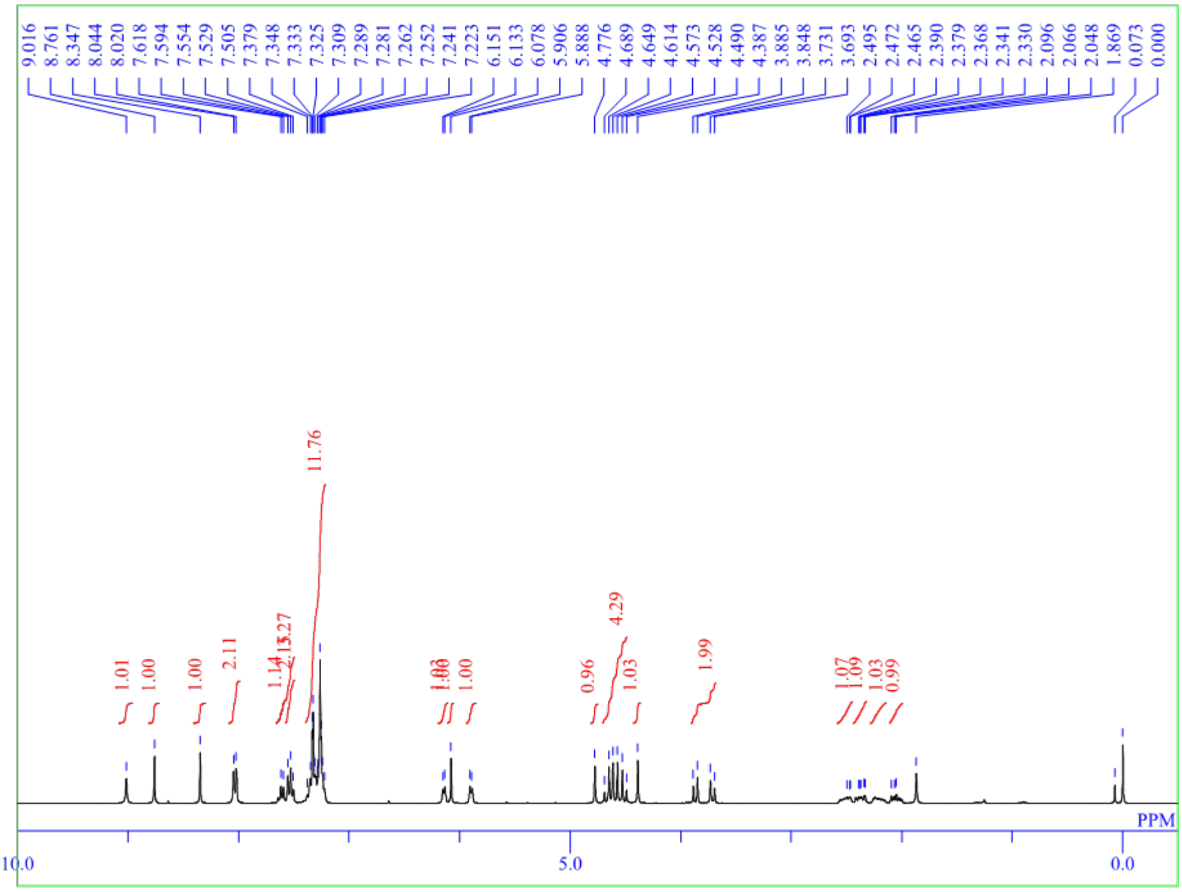


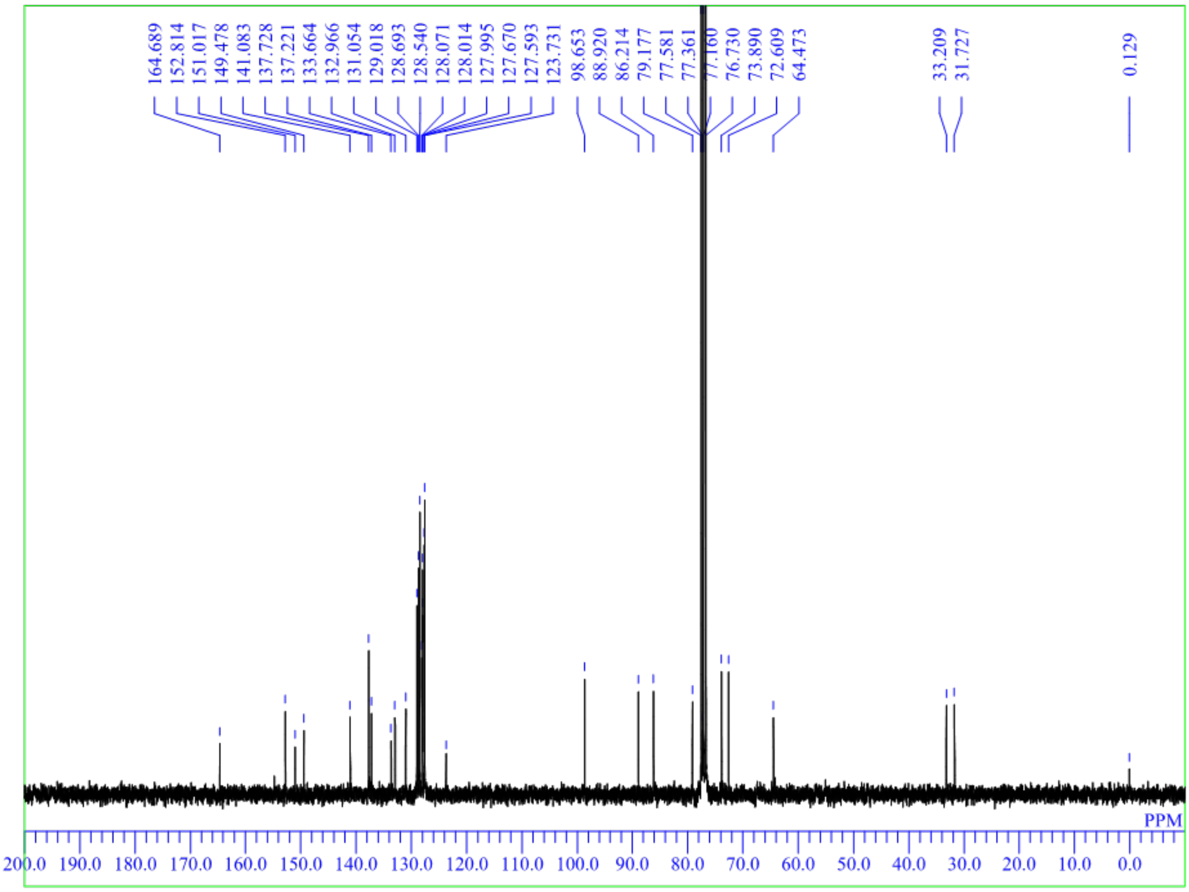

Compound **24** ($^{1}$H-NMR, $CDCl_3$, 300 MHz), ($^{13}$C NMR, $CDCl_3$, 75.6 MHz)

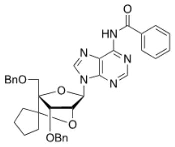


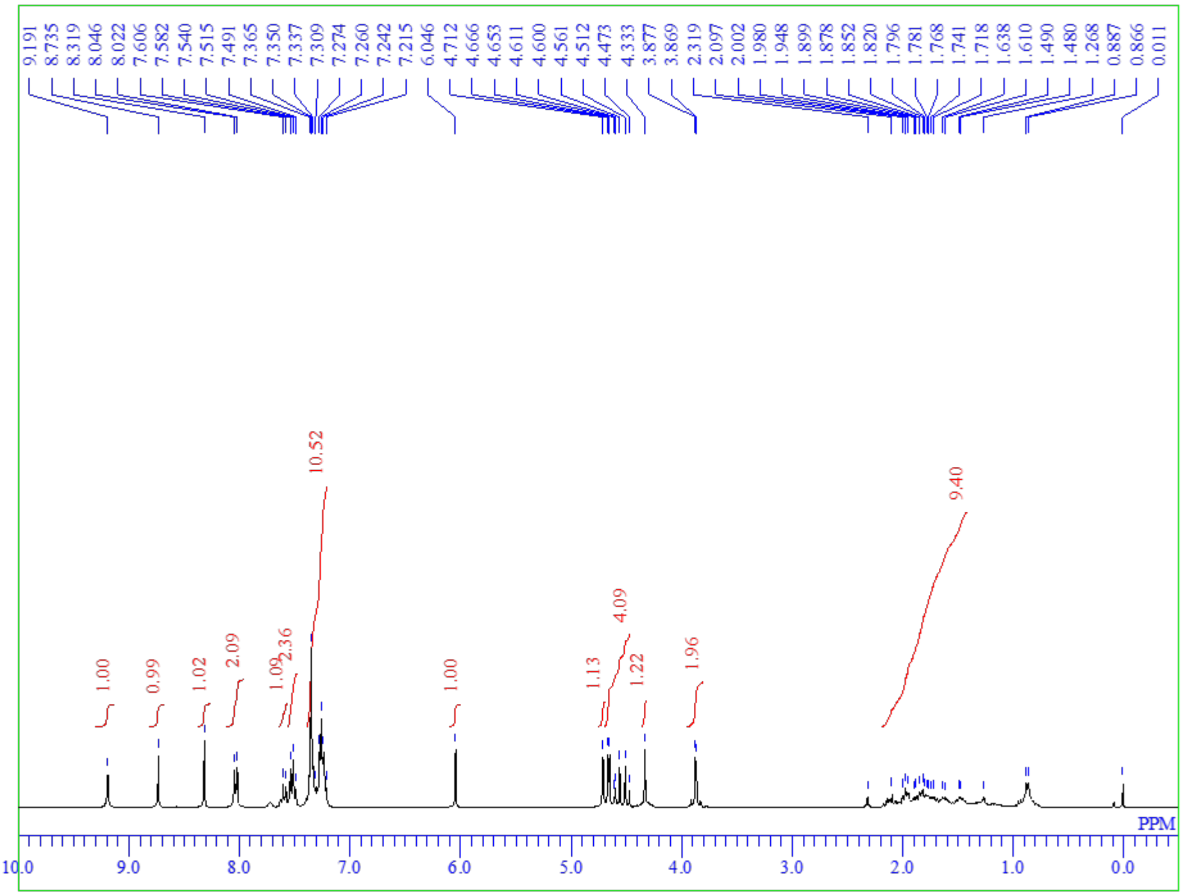


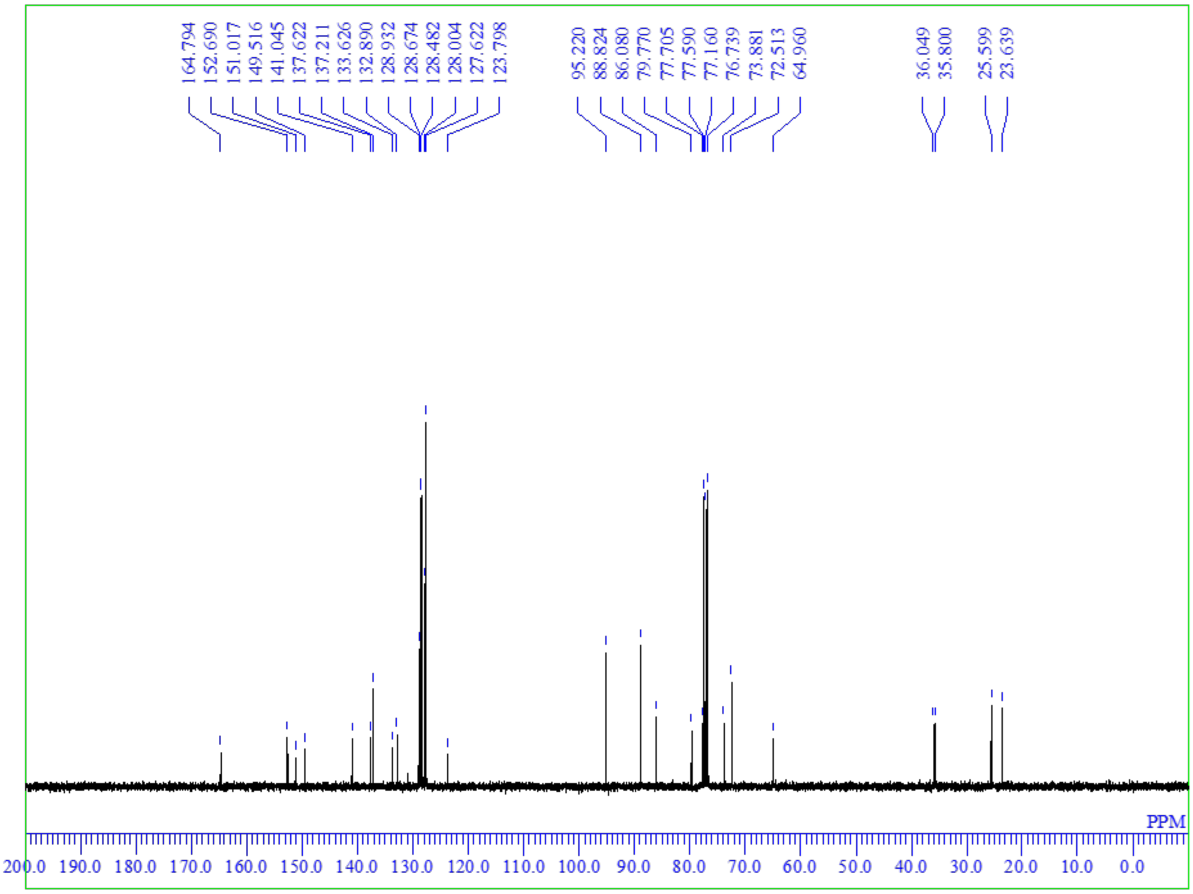

Compound **25** ($^{1}$H-NMR, $CDCl_3$, 300 MHz), ($^{13}$C NMR, $CDCl_3$, 75.6 MHz)

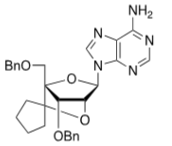


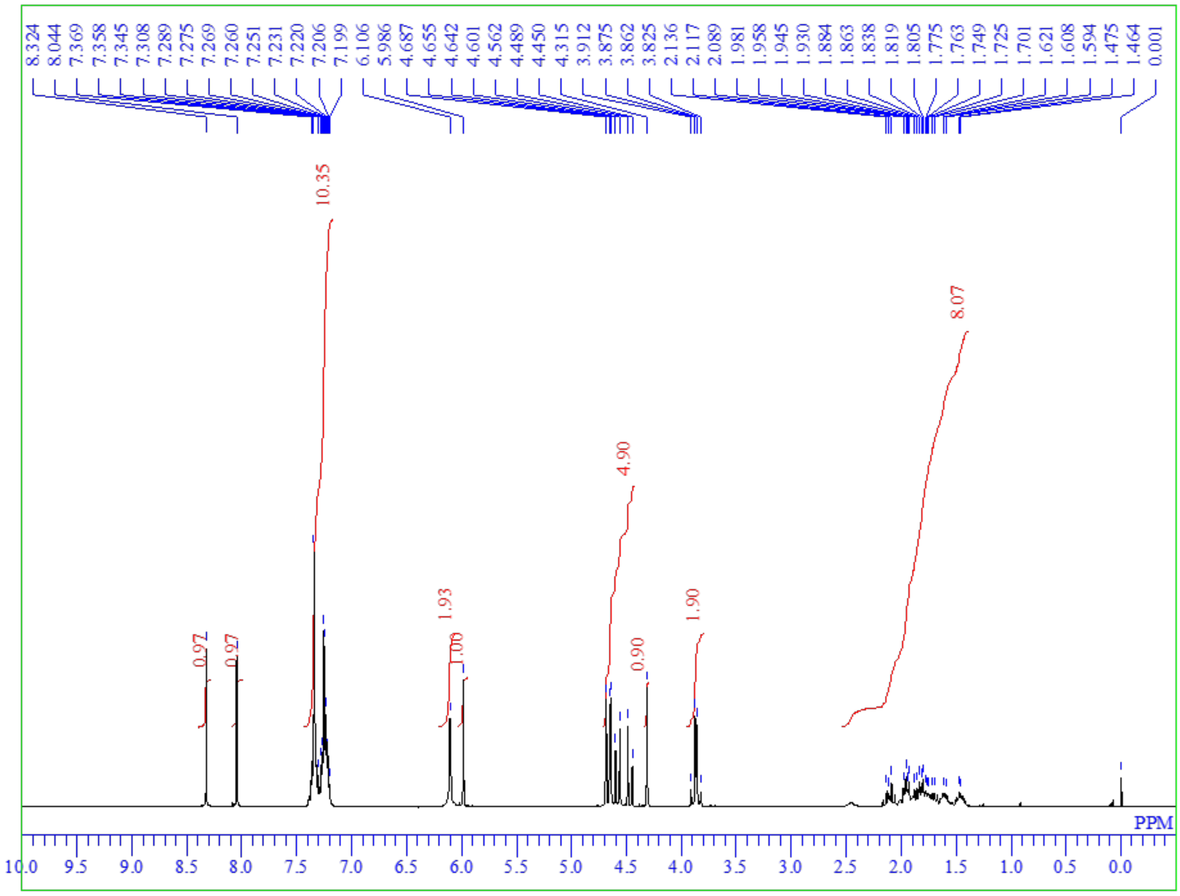


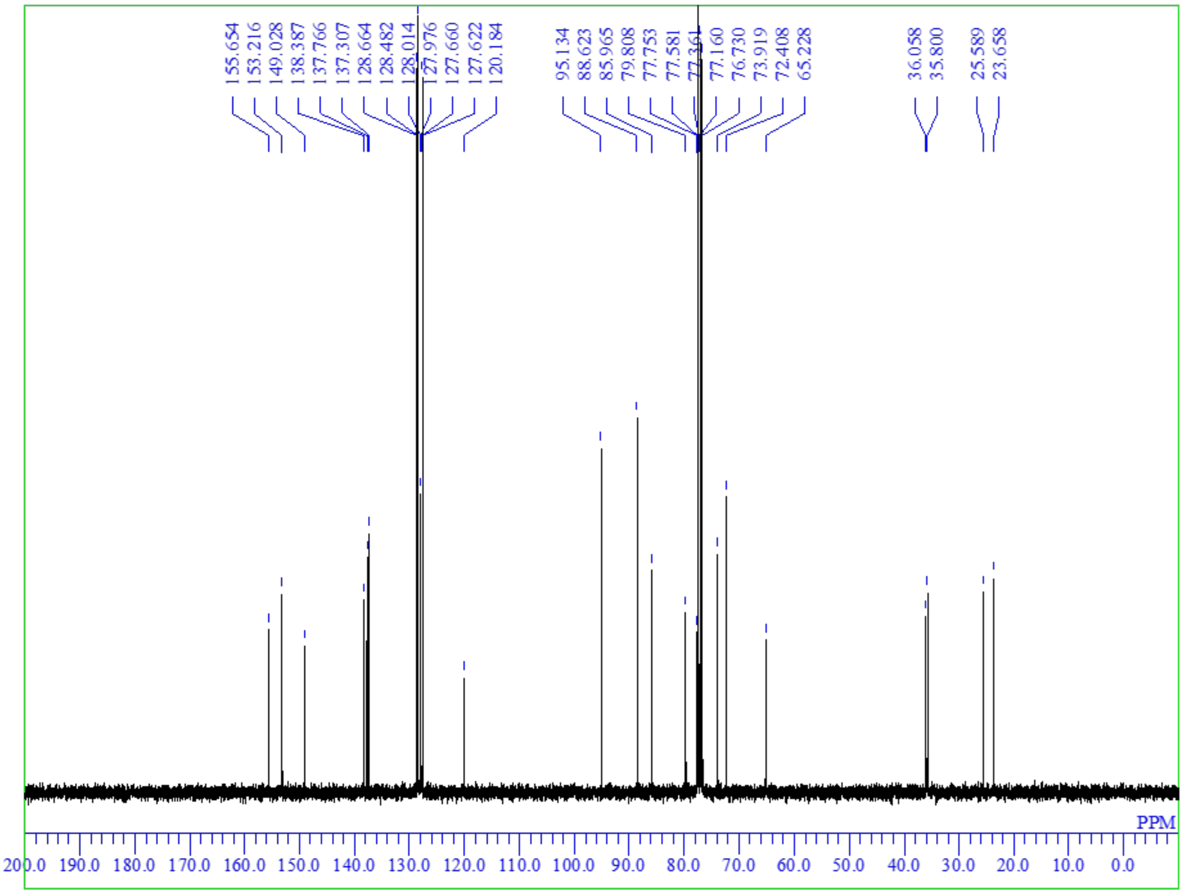

Compound **26** ($^{1}$H-NMR, DMSO-$d_6$, 400 MHz), ($^{1}$H-NMR, DMSO-$d_6$ by a $D_2O$ drop, 400 MHz) and ($^{13}$C NMR, DMSO-$d_6$, 100.6 MHz)

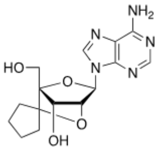

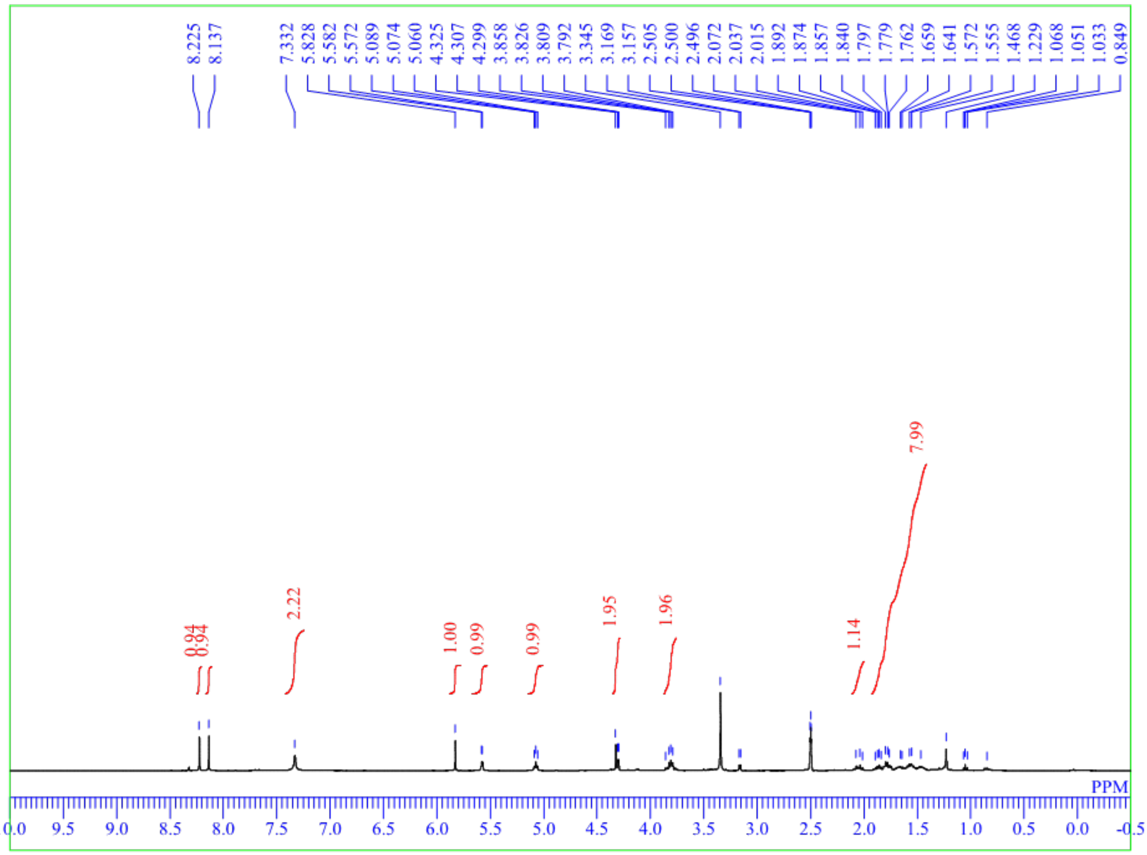

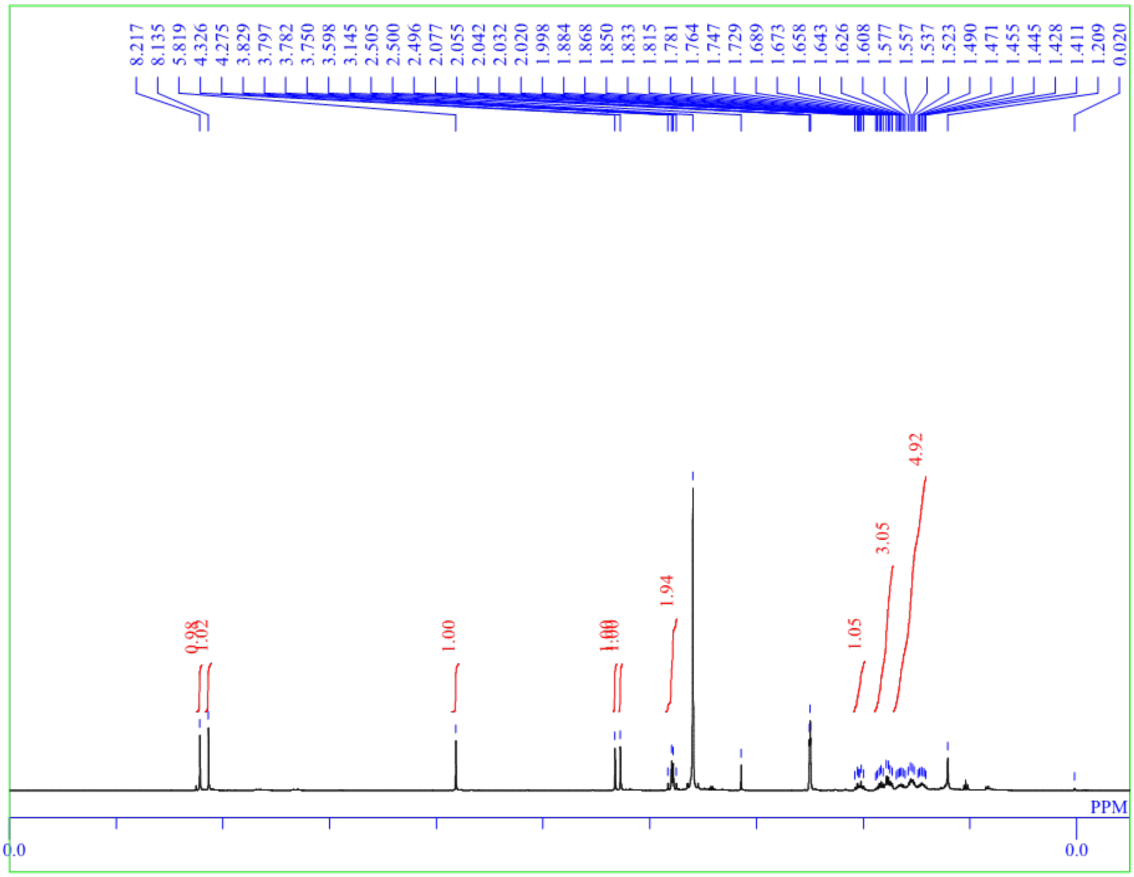

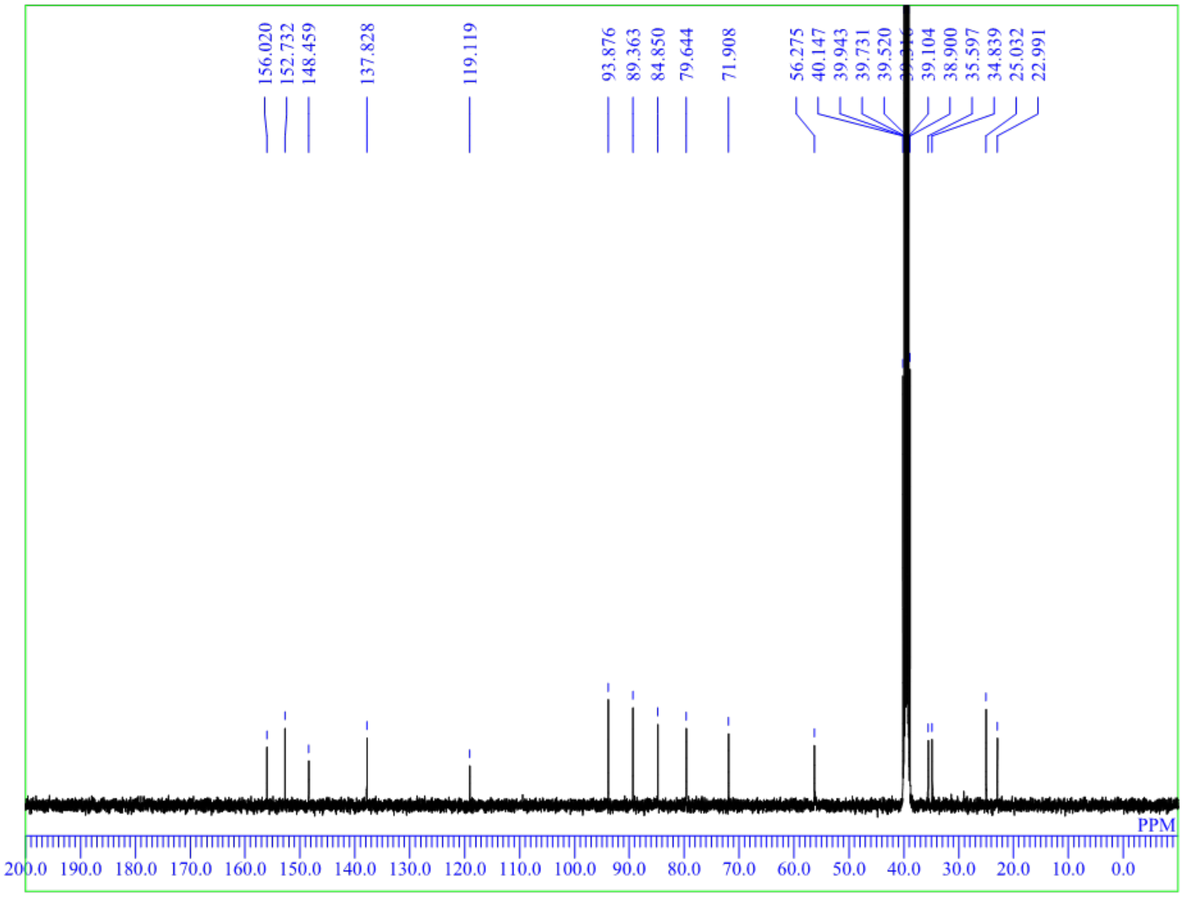
156.020
152.732
148.459
137.828
119.119
93.876
89.363
84.850
79.644
71.908
56.275
40.147
39.943
39.731
39.520
39.104
38.900
35.597
34.839
25.032
22.991
PPM
200.0 190.0 180.0 170.0 160.0 150.0 140.0 130.0 120.0 110.0 100.0 90.0 80.0 70.0 60.0 50.0 40.0 30.0 20.0 10.0 0.0

Compound **29** ($^{1}$H-NMR, $CDCl_3$, 300 MHz), ($^{13}$C NMR, $CDCl_3$, 75.6 MHz)

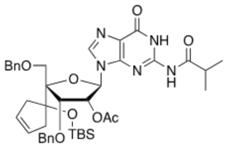


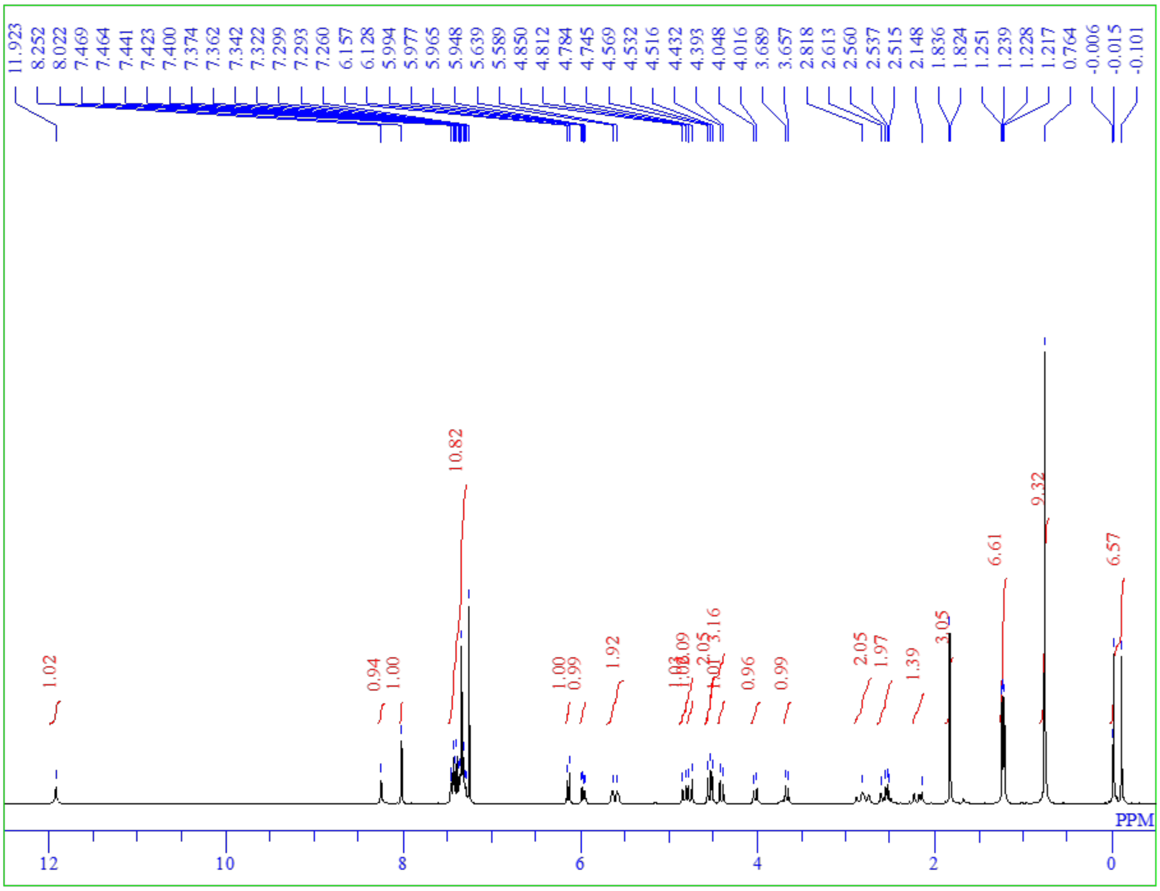


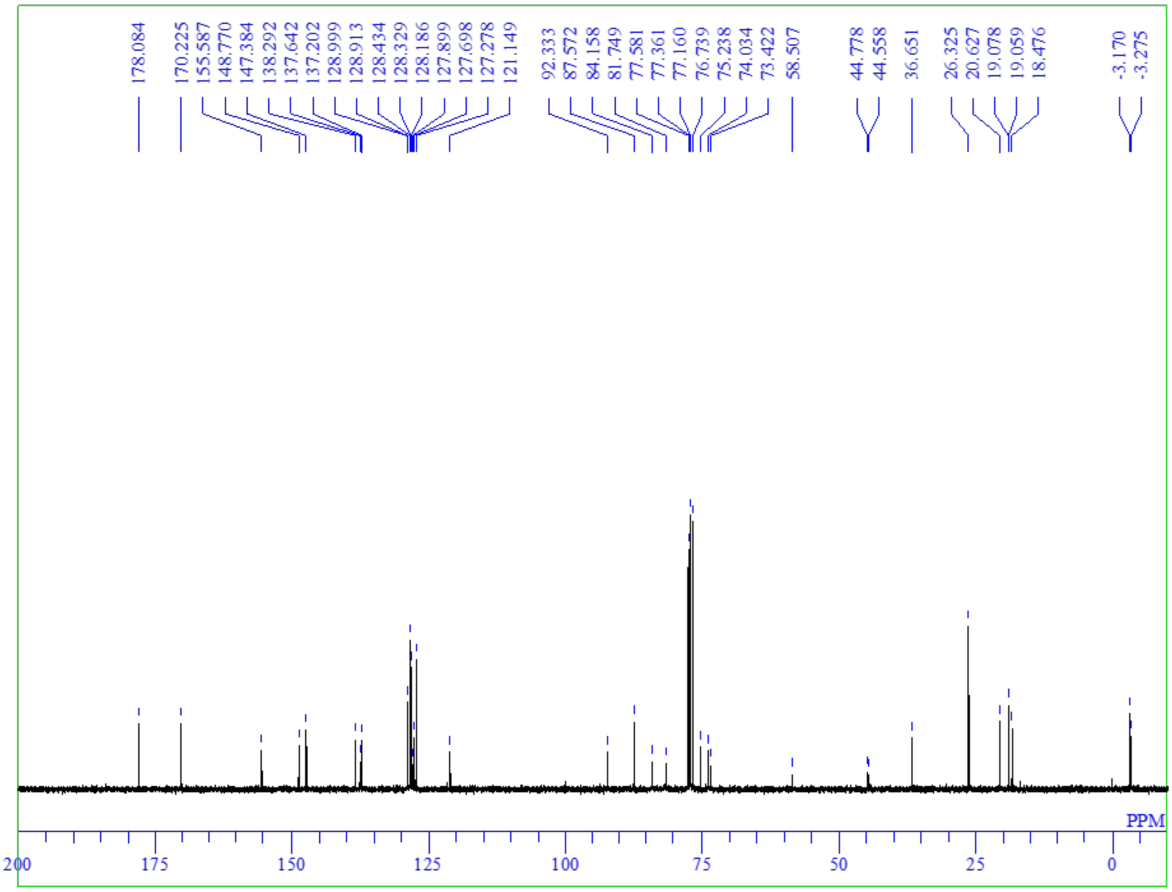

Compound **30** ($^1$H-NMR, $CDCl_3$, 300 MHz), ($^{13}$C NMR, $CDCl_3$, 125.8 MHz)

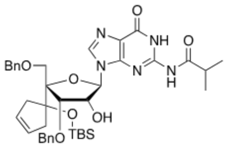


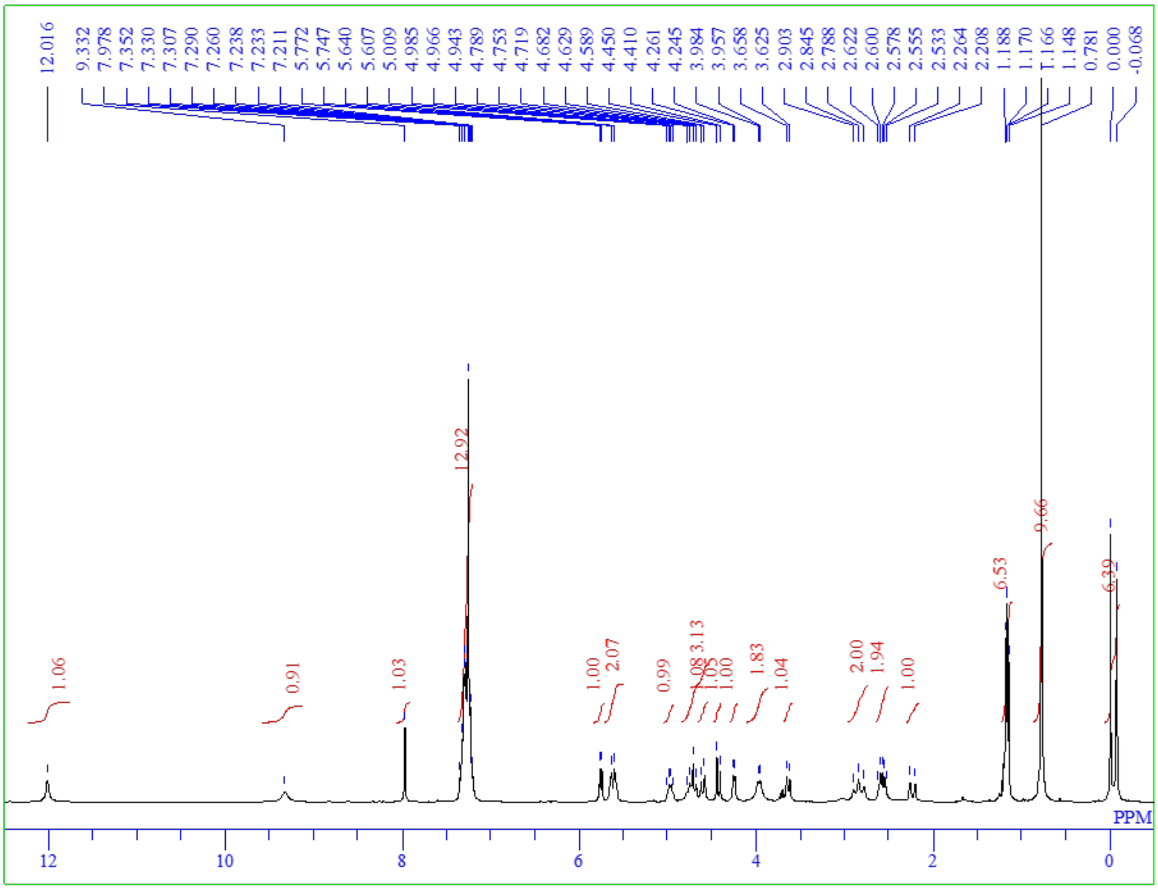


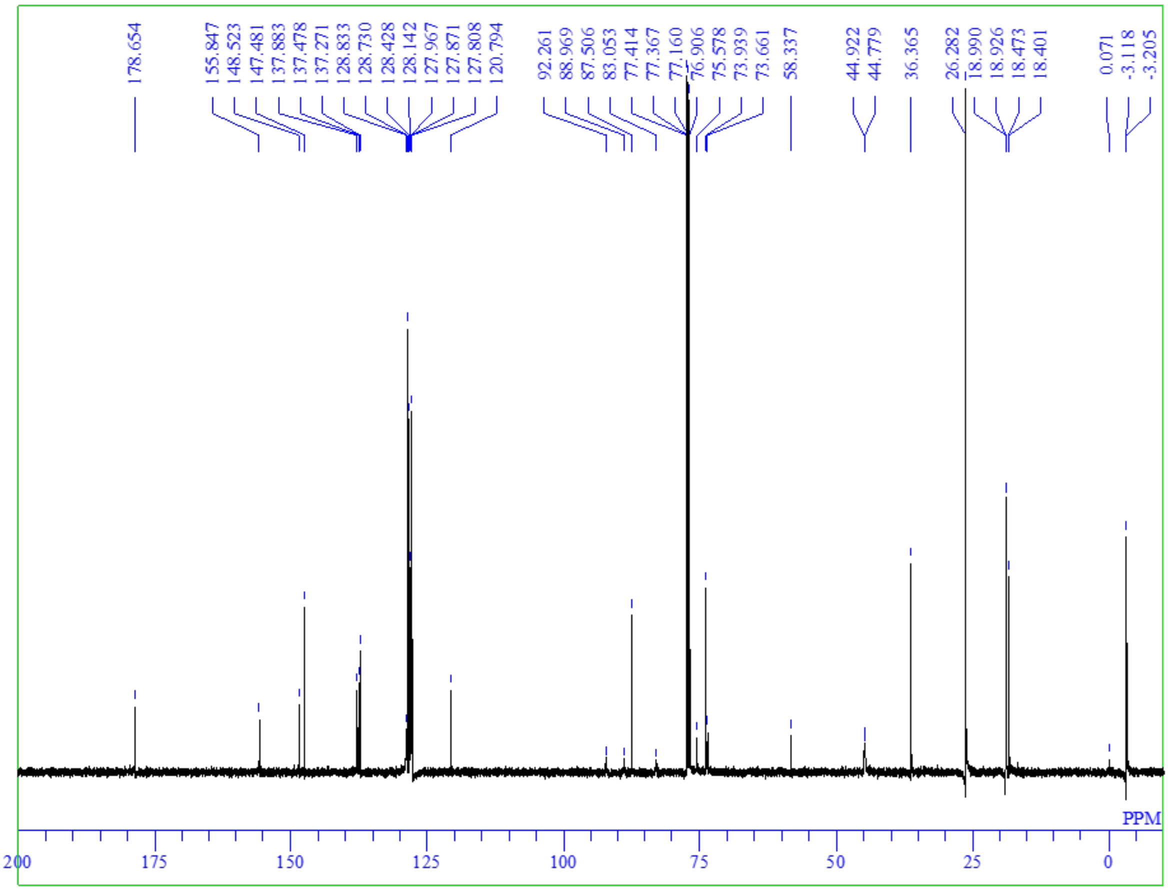

Compound **30** and **32** ($^{1}$H-NMR, $CDCl_3$, 400 MHz), ($^{13}$C NMR, $CDCl_3$, 125.8 MHz)

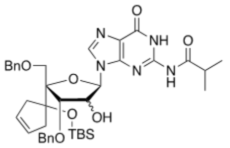


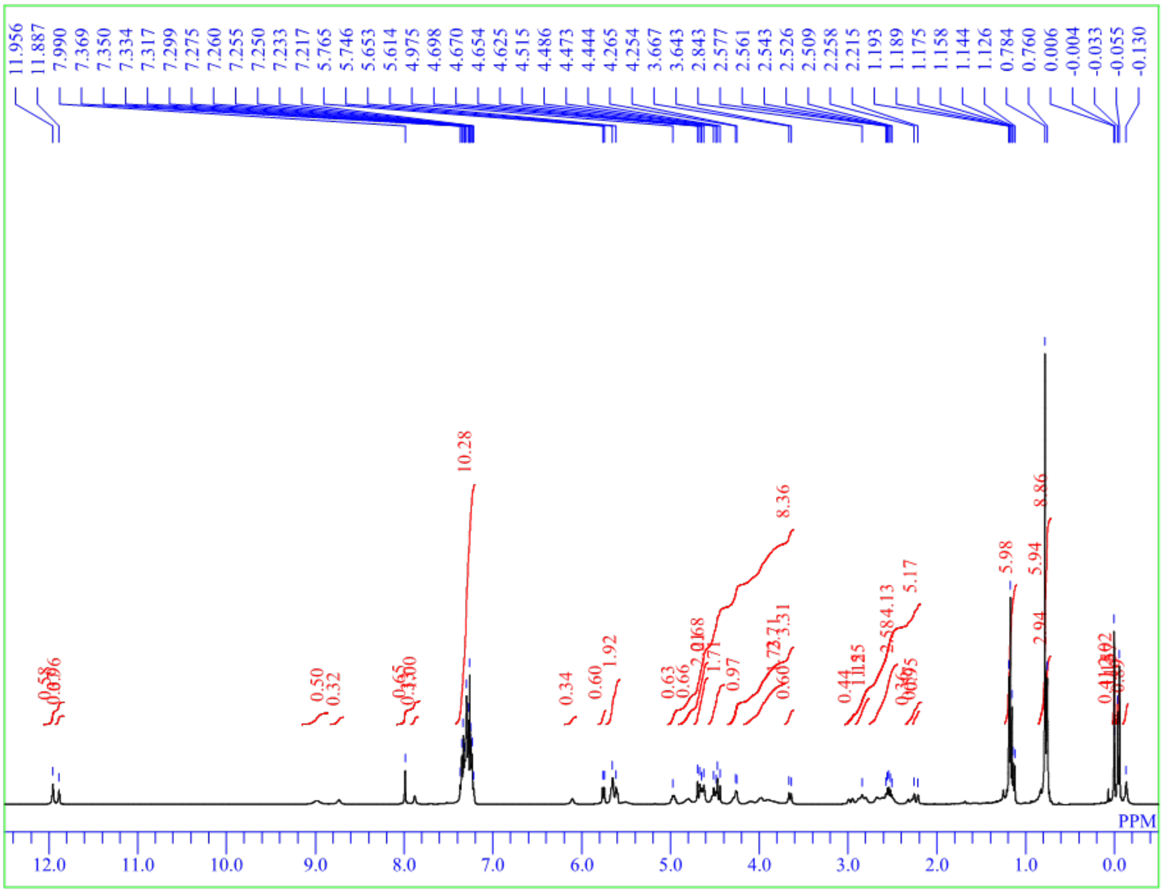


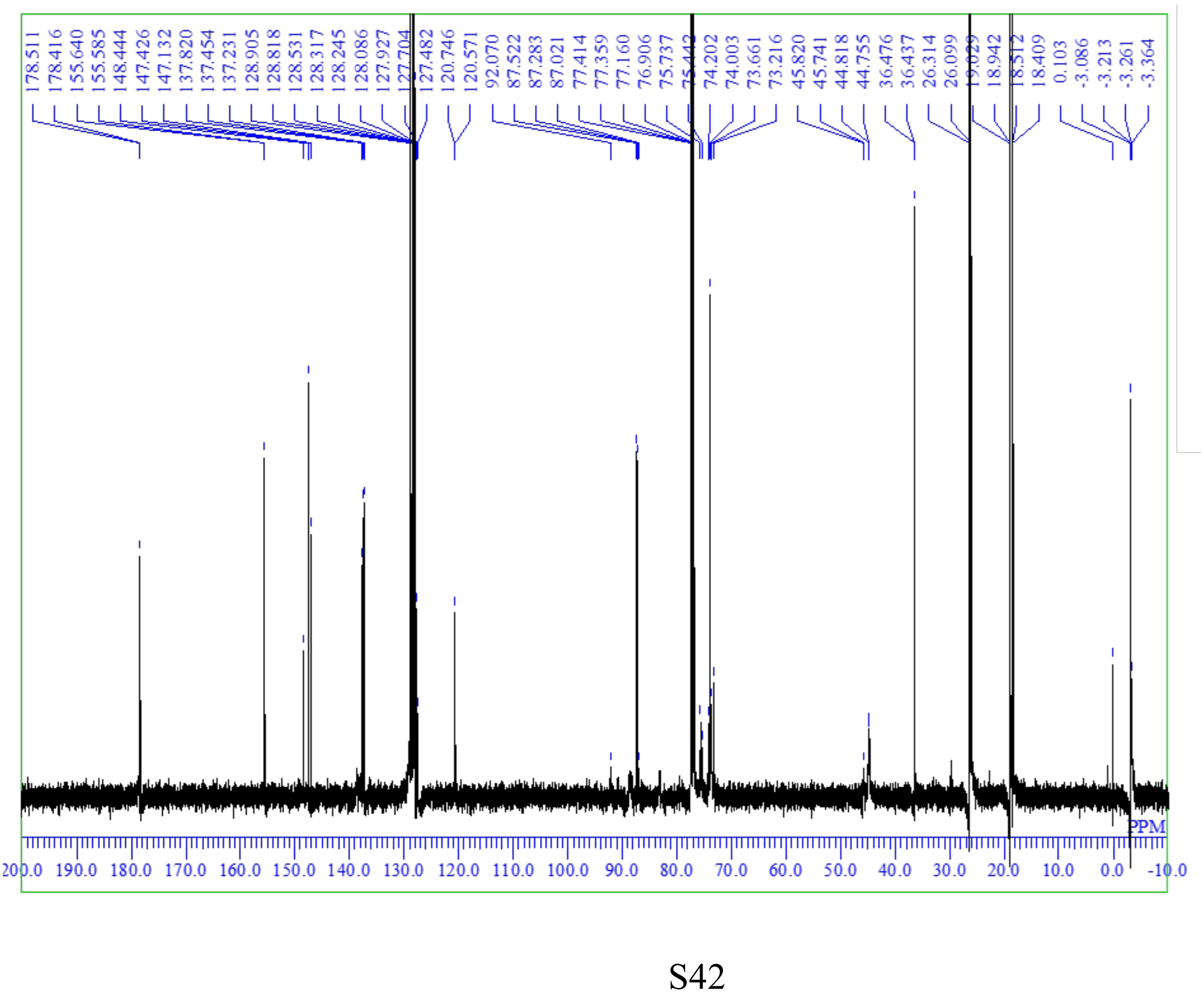

**ribo-methylate** ($^1$H-NMR, $CDCl_3$, 400 MHz), ($^{13}$C NMR, $CDCl_3$, 100.6 MHz)

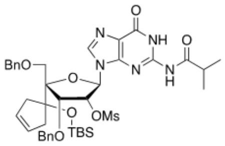


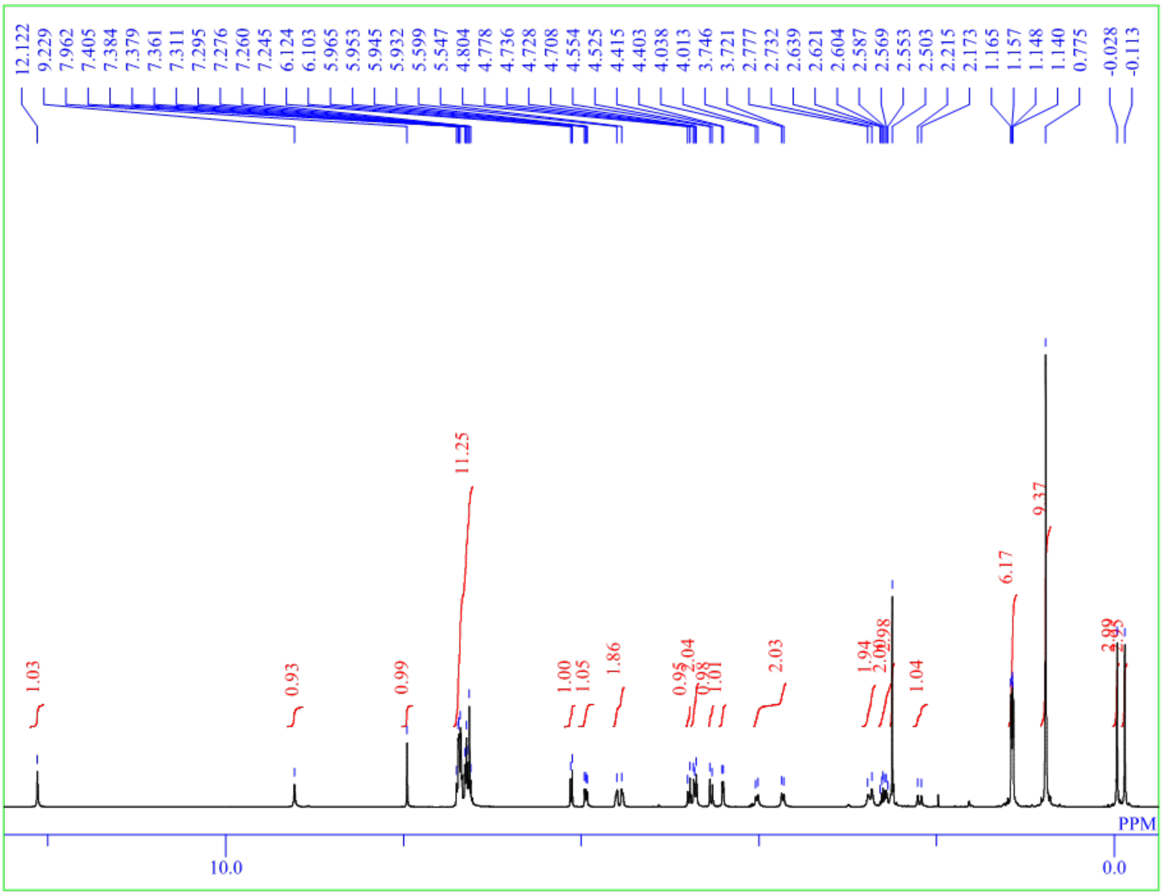

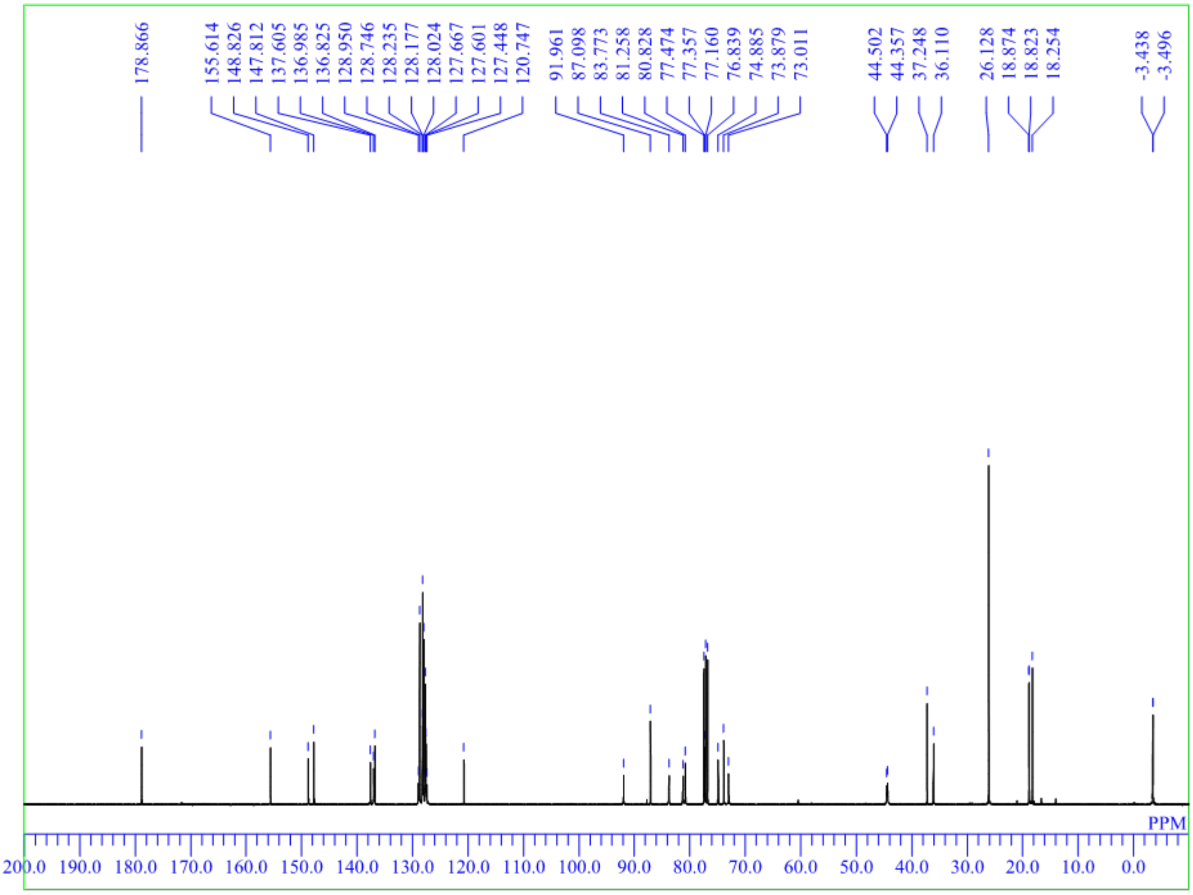

Compound **33** ($^{1}$H-NMR, $CDCl_3$, 400 MHz), ($^{13}$C NMR, $CDCl_3$, 100.5 MHz)

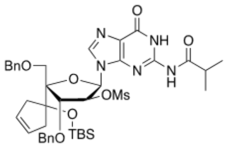

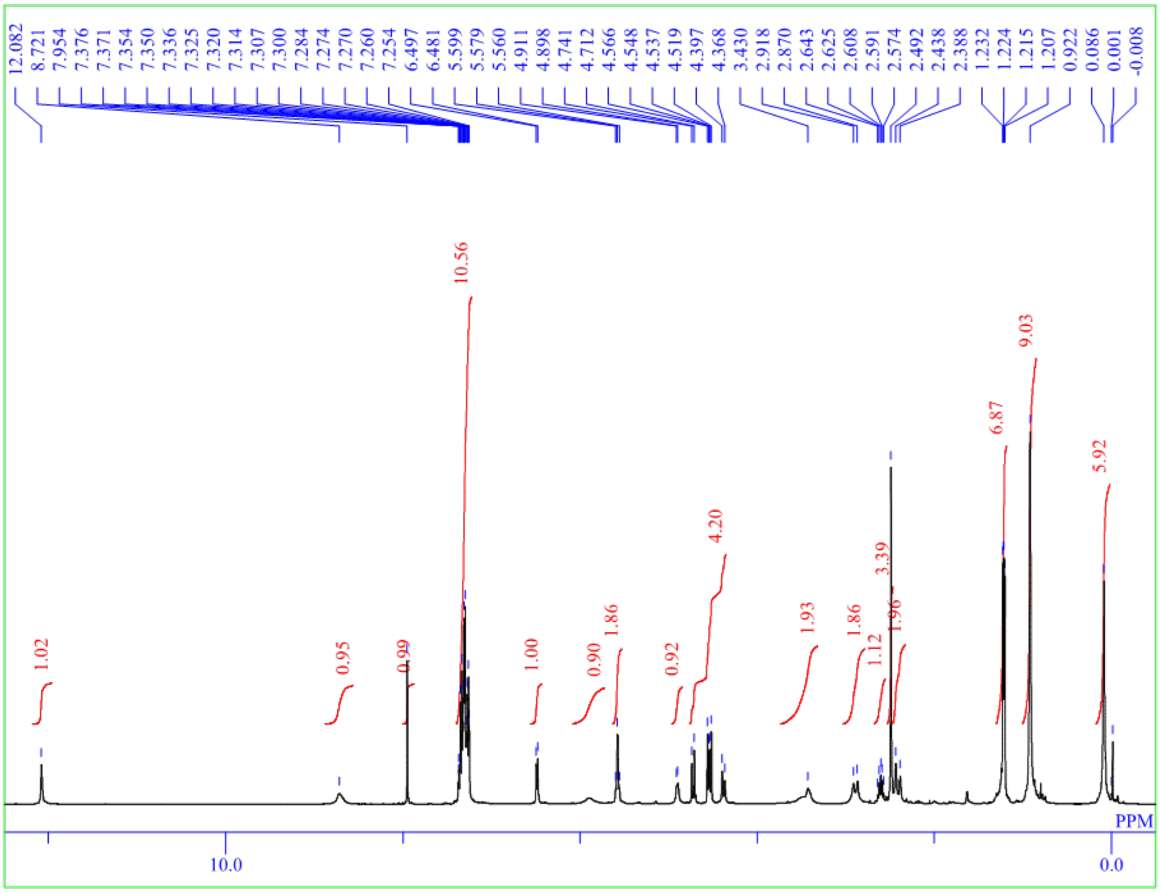

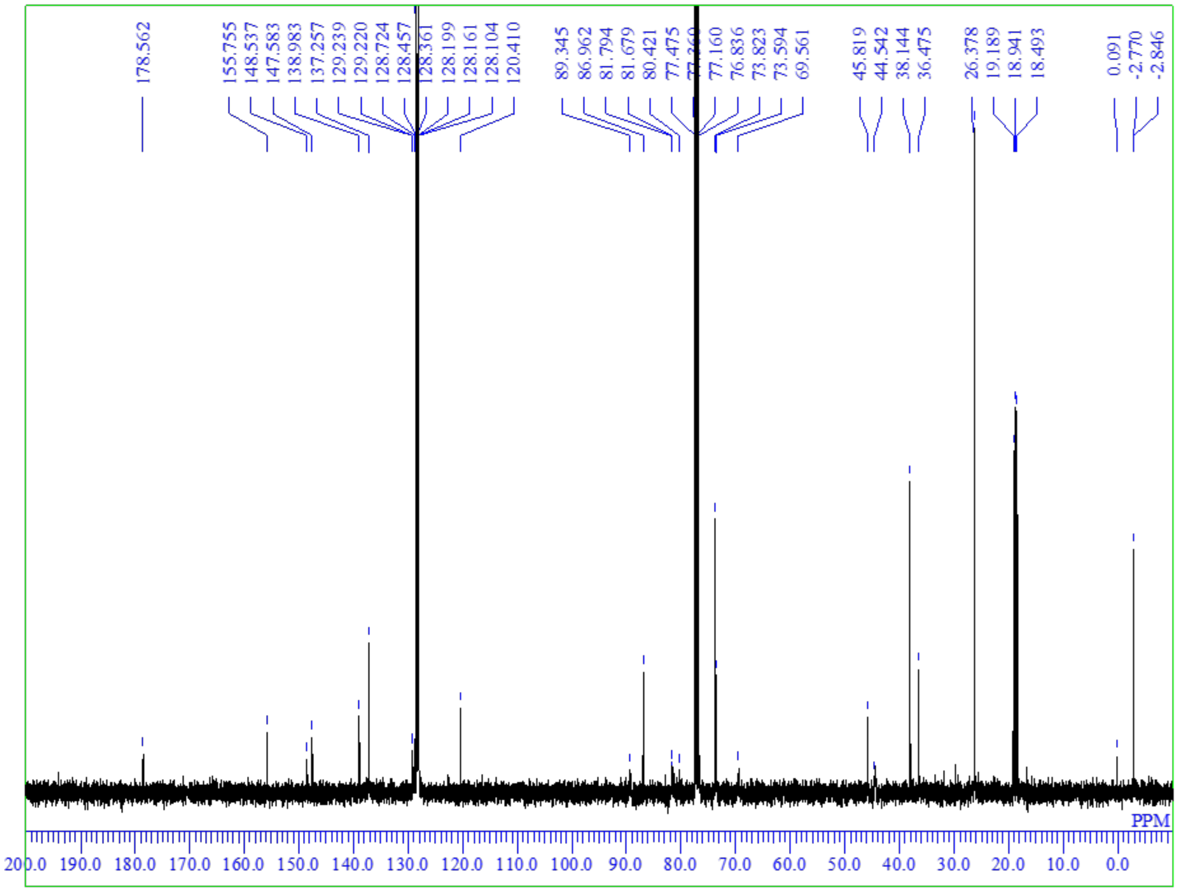

Compound **34** ($^1$H-NMR, $CDCl_3$, 400 MHz), ($^{13}$C NMR, $CDCl_3$, 100.6 MHz)

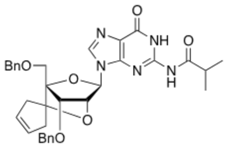

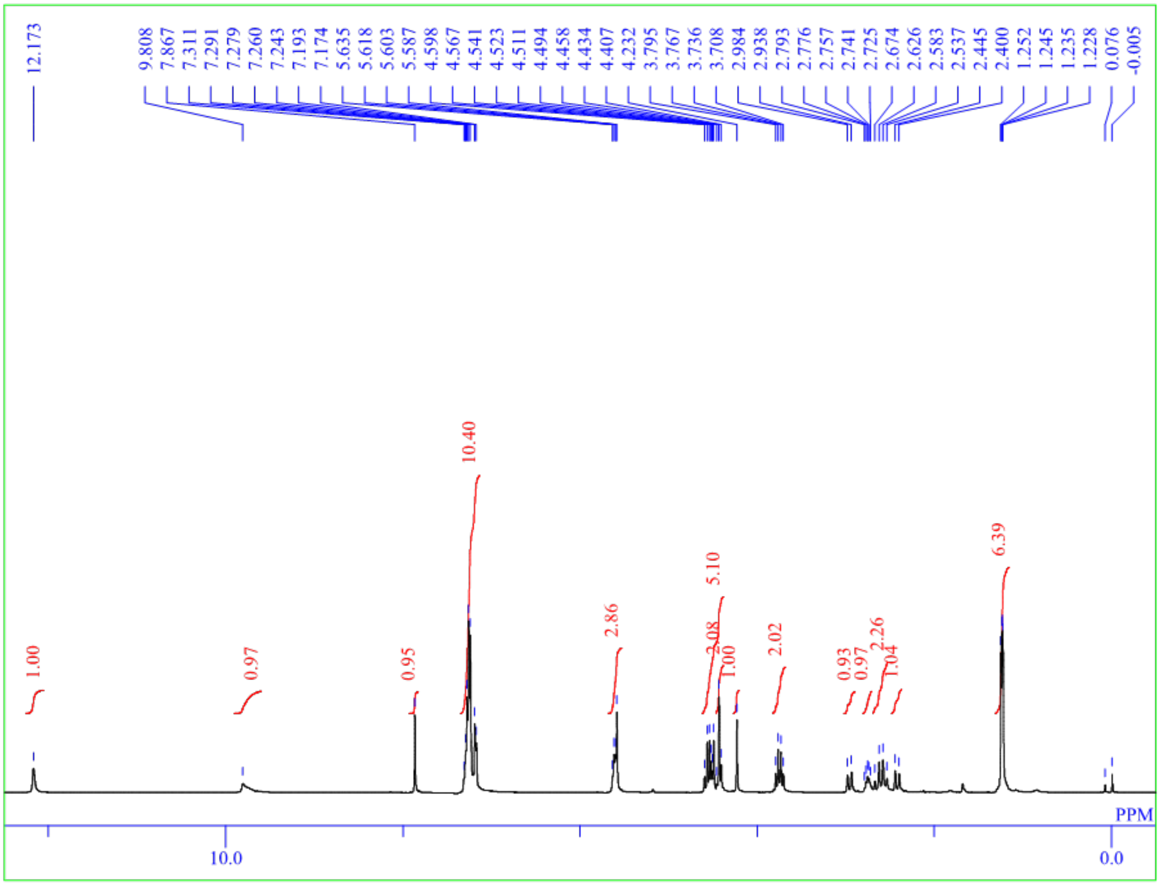

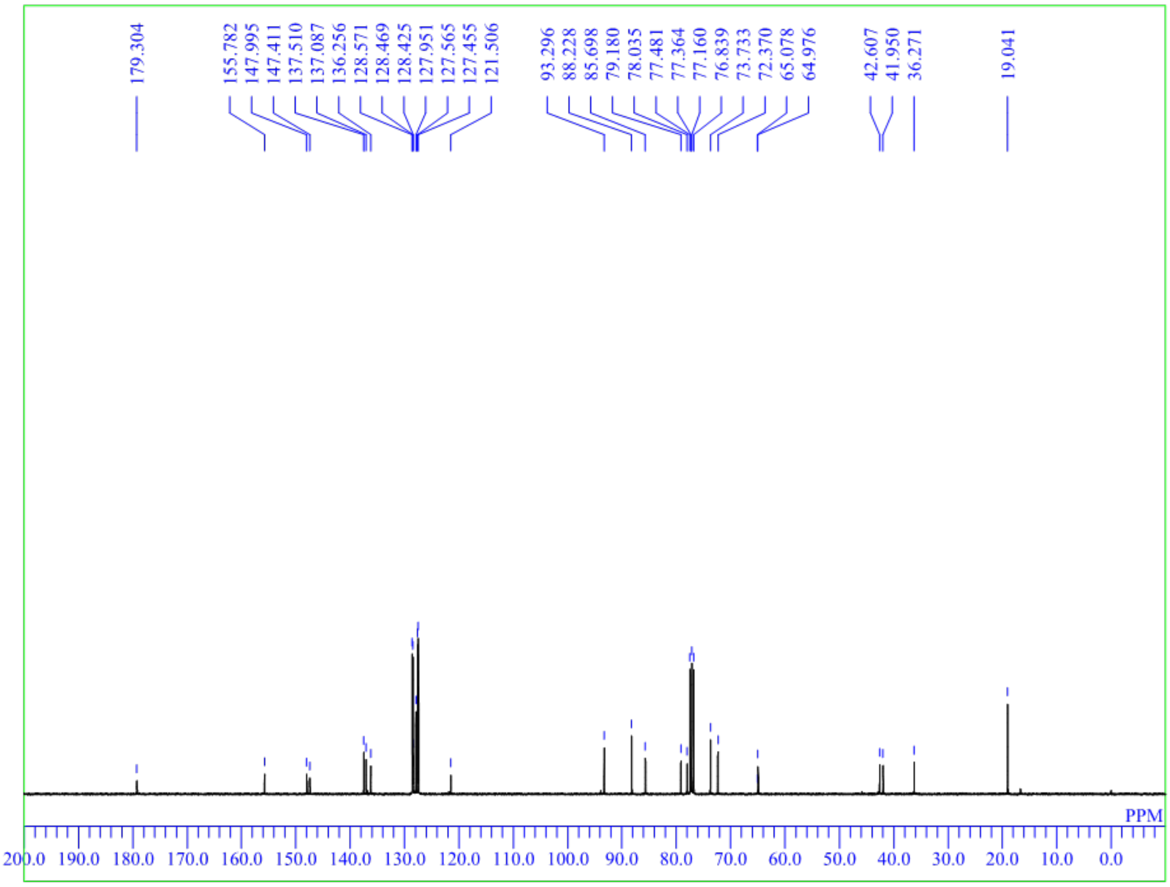

Compound **35** ($^1$H-NMR, $CD_3OD$, 300 MHz), ($^{13}$C NMR, $CD_3OD$, 75.6 MHz)

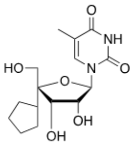


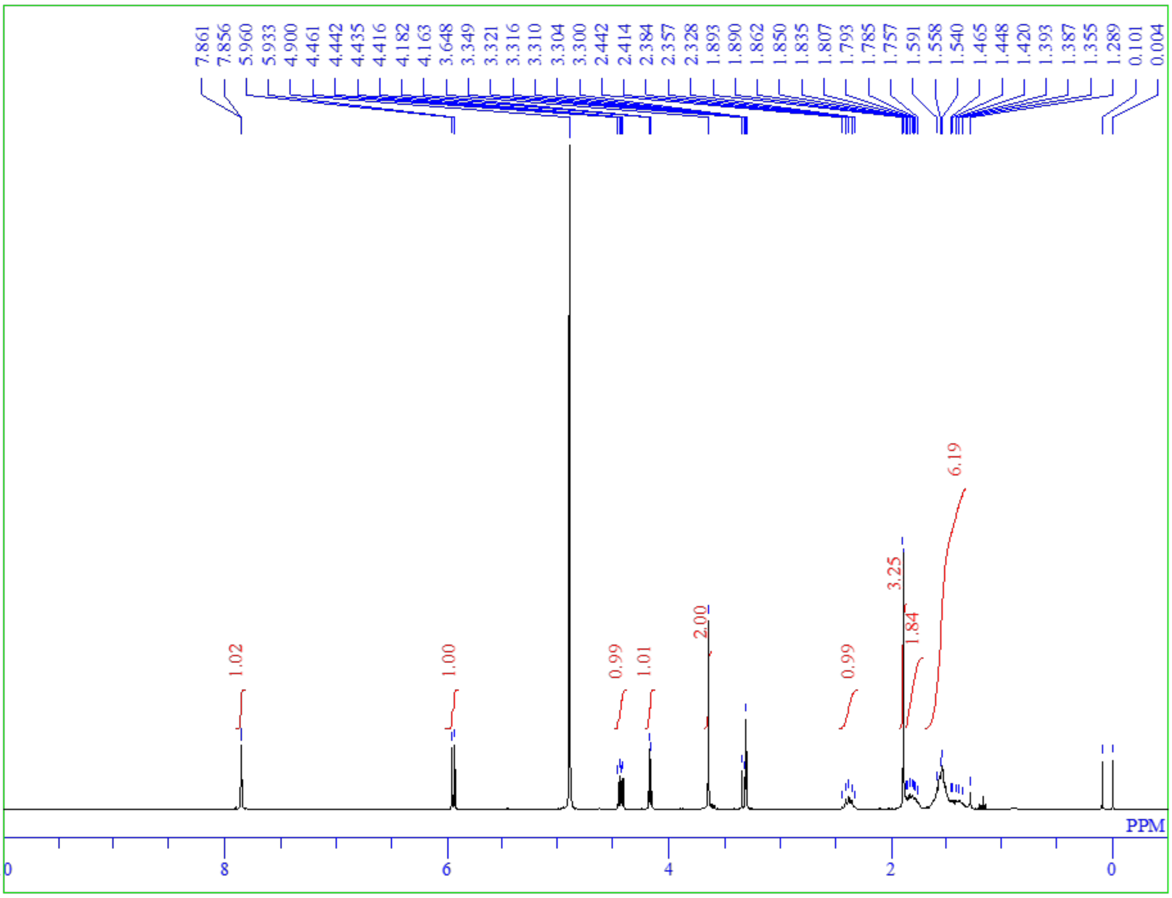


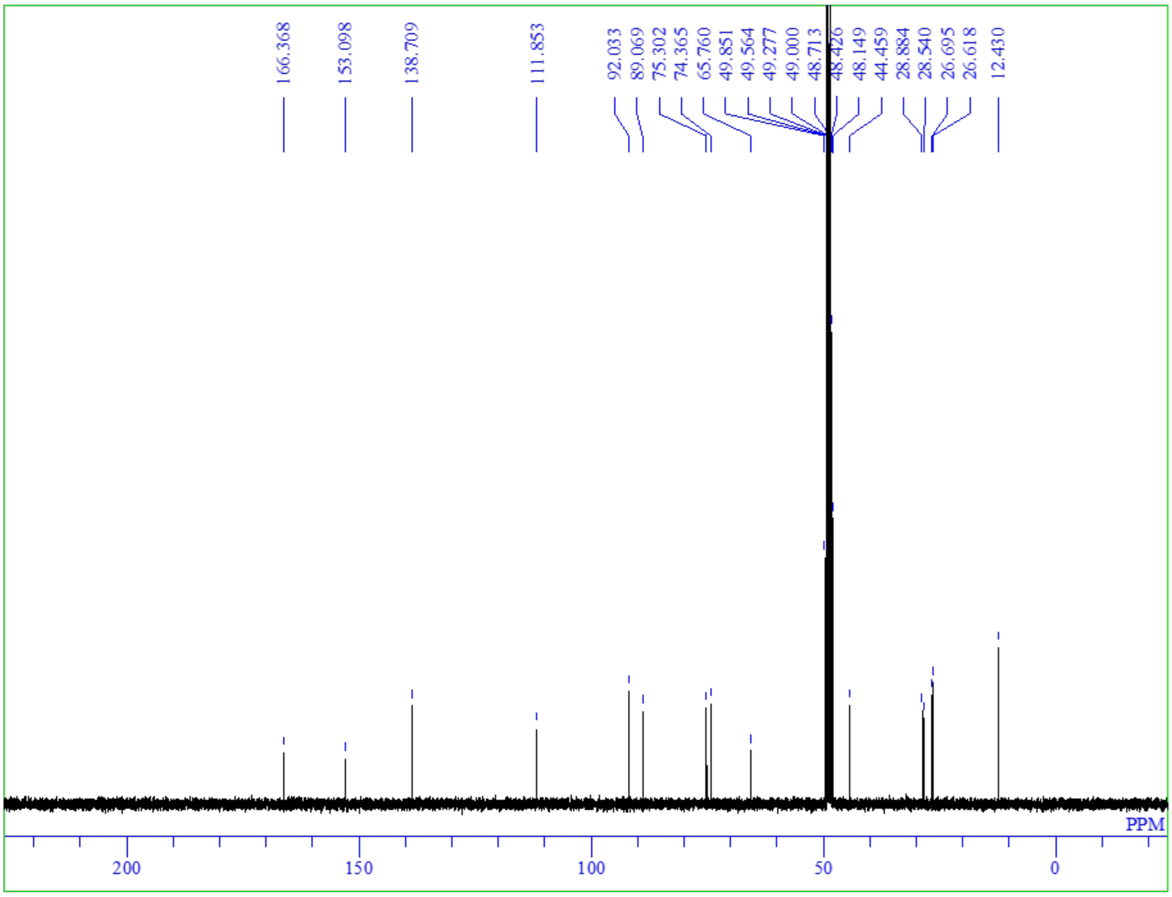

Supplement: Supplementary file 1 — Supporting Information [file CHEM-31-e02995-s001.pdf]
